# Synthesis, Antioxidant Activity, In Silico Evaluation, and Cytotoxicity of Novel Polyphenolic–Methyliden–Hydrazono–Thiazole Derivatives

**DOI:** 10.3390/molecules31203620

**Published:** 2026-10-11

**Authors:** Alexandra Cătălina Cornea, Gabriel Marc, Ionel Fizeșan, Ioana Ionuț, Cristina Moldovan, Anca Stana, Brîndușa Tiperciuc, Ionuț-Valentin Creștin, Adrian Pîrnău, Laurian Vlase, Ilioara Oniga, Ovidiu Oniga

**Affiliations:** 1 Department of Pharmaceutical Chemistry, “Iuliu Hațieganu” University of Medicine and Pharmacy, 41 Victor Babeș Street, 400012 Cluj-Napoca, Romaniaionut.ioana@umfcluj.ro (I.I.); stana.anca@umfcluj.ro (A.S.);; 2 Department of Organic Chemistry, “Iuliu Hațieganu” University of Medicine and Pharmacy, 41 Victor Babeș Street, 400012 Cluj-Napoca, Romania; 3 Department of Toxicology, Faculty of Pharmacy, “Iuliu Hațieganu” University of Medicine and Pharmacy, 8 Victor Babeș, 400012 Cluj-Napoca, Romania; ionel.fizesan@umfcluj.ro (I.F.);; 4 National Institute for Research and Development of Isotopic and Molecular Technologies, 67-103 Donath Street, 400293 Cluj-Napoca, Romania; adrian.pirnau@itim-cj.ro; 5 Department of Pharmaceutical Technology and Biopharmaceutics, “Iuliu Hațieganu” University of Medicine and Pharmacy, 41 Victor Babeș Street, 400012 Cluj-Napoca, Romania; 6 Department of Pharmacognosy, “Iuliu Hațieganu” University of Medicine and Pharmacy, 12 Ion Creangă Street, 400010 Cluj-Napoca, Romania; ioniga@umfcluj.ro

**Keywords:** antioxidant, phenol, thiazole, ROS, anticancer, DFT

## Abstract

Novel thiazolyl–polyphenolic derivatives were designed and synthesized to investigate the influence of aromatic substituents on antioxidant activity, molecular properties, and cytotoxicity. Eight derivatives (**E1**–**E8**) were synthesized by a Hantzsch reaction and their structures were confirmed by MS, FT-IR, and ^1^H/^13^C NMR spectroscopy. Antioxidant activity was evaluated using DPPH, ABTS, TAC, RP, FRAP, and CUPRAC assays. The experimental results were supported by DFT calculations and SwissADME prediction, while cytotoxicity was evaluated against A549 lung adenocarcinoma cells and normal BJ fibroblasts. The antioxidant activity depended on the nature of the aromatic substituent. Compound **E5** showed a superior antioxidant profile, presenting the highest DPPH radical scavenging activity and the strongest reducing power in the RP assay. The theoretical calculations supported these findings, showing favorable HOMO energy and low O–H bond dissociation energy values. Compound **E6** showed the highest activity in the CUPRAC assay and exhibited the strongest cytotoxic activity against A549 cells together with the highest selectivity index. All synthesized compounds complied with Lipinski’s rule of five. Overall, the results show that the electronic properties of the aromatic substituent strongly influence both the antioxidant and cytotoxic activities of this series. These compounds may represent useful scaffolds for further development of antioxidant and anticancer agents.

## 1. Introduction

Thiazole-containing compounds represent an important class of privileged scaffolds in medicinal chemistry, being incorporated into numerous clinically relevant agents, including the antineoplastic kinase inhibitor dasatinib, the antitumor microtubule stabilizer ixabepilone, and the antibacterial siderophore cephalosporin cefiderocol [1,2,3,4,5]. Their pharmacological relevance arises from the ability of the thiazole nucleus to modulate the electronic distribution, molecular polarity, and intermolecular interaction profile of bioactive molecules, thereby influencing both physicochemical properties and biological activity. Consequently, thiazole derivatives have been extensively investigated as promising candidates for the development of antioxidant, anti-inflammatory, antimicrobial, and anticancer agents [1,6,7,8].

Polyphenols constitute another major class of biologically active compounds recognized for their capacity to modulate cellular redox homeostasis through hydrogen atom transfer and electron transfer mechanisms. Beyond their well-established antioxidant properties, polyphenols have been shown to exert anti-inflammatory, antiproliferative, proapoptotic, cardioprotective, neuroprotective, and antimicrobial effects through the modulation of multiple signaling pathways involved in inflammation, cell survival, apoptosis, and tumor progression [9,10,11,12,13,14,15].

Among naturally occurring polyphenols, resveratrol has emerged as one of the most extensively investigated stilbene derivatives due to its abundance in plant-derived sources and its broad spectrum of biological activities, including antioxidant, anti-inflammatory, cardioprotective, neuroprotective, and anticancer effects mediated through the regulation of oxidative stress and multiple cellular signaling networks [16,17].

Oxidative stress, resulting from an imbalance between reactive oxygen species (ROS) generation and antioxidant defense mechanisms, is recognized as a key contributor to the pathogenesis of cancer, cardiovascular diseases, neurodegenerative disorders, chronic inflammatory conditions, and age-related pathologies. Although ROS are essential mediators of physiological signaling processes, their excessive accumulation promotes oxidative damage to lipids, proteins, and nucleic acids, ultimately contributing to disease initiation and progression [6,7,18,19,20,21]. Consequently, the identification of compounds capable of modulating redox homeostasis while simultaneously exerting antiproliferative effects represents an attractive strategy in contemporary drug discovery.

Despite the extensive literature describing the biological properties of both thiazole derivatives and polyphenolic compounds, hybrid molecular systems integrating these two pharmacophoric motifs remain comparatively underexplored. In our previous works, we investigated polyphenolic thiazole derivatives as antioxidant agents, some of them being N-substituted on the thiazole, while the present study therefore extends the previous research, providing a new structural variable for tuning the antioxidant and biological properties of this class of compounds. Furthermore, limited information is available regarding the influence of electronic modulation induced by electron-donating and electron-withdrawing substituents on the balance between antioxidant and antiproliferative activities within such hybrid structures. We hypothesized that the combination of a redox-active polyphenolic framework with a thiazole-based pharmacophore, coupled with systematic electronic modulation of the aryl substituent, could generate multifunctional molecules displaying distinct antioxidant and cytotoxic profiles. To test this hypothesis, a series of novel thiazolyl–polyphenolic derivatives was designed and synthesized, and the relationship between structural electronic effects, antioxidant behavior, predicted molecular properties, and cytotoxic activity was investigated.

## 2. Results

### 2.1. Synthetic Approach and Structural Characterization of Polyphenolic–Methyliden–Hydrazono–Thiazole Derivatives

**Scheme 1:**
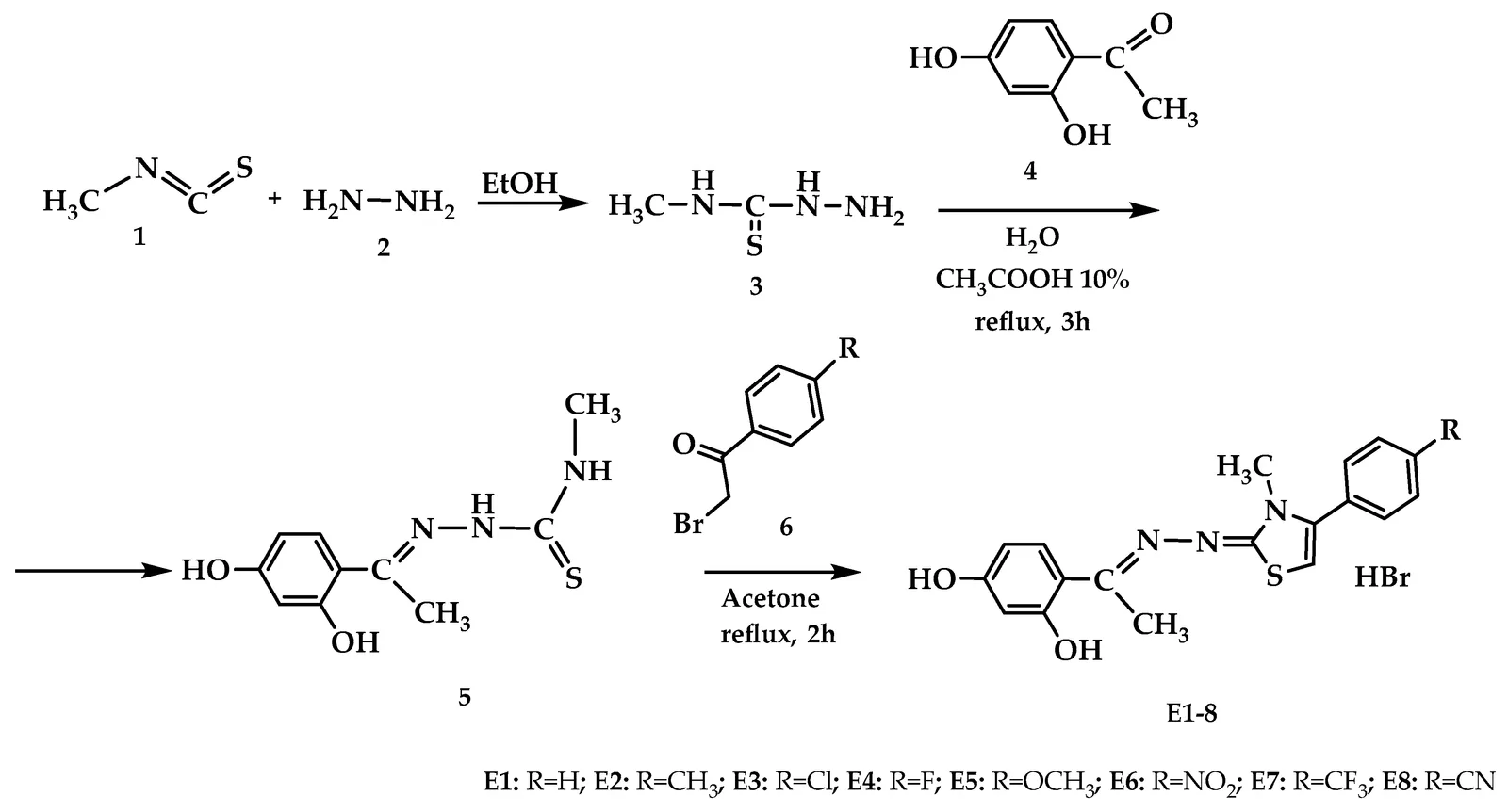
Synthetic route to thiazolyl–polyphenolic derivatives (**E1**–**E8**).

Compounds **E1**–**E8** were synthesized following the synthetic route outlined in Scheme 1 through a Hantzsch reaction. The thiazolyl–polyphenolic derivatives were obtained in moderate to good yields (56–77%). The structures of synthesized derivatives were confirmed by mass spectrometry, FT-IR, and ^1^H/^13^C NMR spectroscopy. The analytical data were in full agreement with the proposed molecular structures, while the spectroscopic features were consistent with the expected substitution patterns of the corresponding thiazole derivatives. Detailed structural characterization data together with the corresponding spectra are provided in the Supplementary Materials.

### 2.2. In Vitro Assessment of Antioxidant Activity

#### 2.2.1. Antiradical Assays

The antioxidant potential of compounds **E1**–**E8** was evaluated through DPPH^•^ and ABTS^•+^ radical scavenging assays. The IC_50_ values obtained are presented in Table 1 in comparison with Trolox as the reference antioxidant.

In the DPPH assay, compound **E5** exhibited the lowest IC_50_ value (92.32 μM) among the synthesized derivatives, whereas compound **E6** did not reach 50% inhibition within the tested concentration range. The markedly reduced DPPH scavenging activity of **E6** may be associated with the strong electron-withdrawing character of the nitro substituent. The –NO_2_ group decreases the electron density of the conjugated aromatic system and may consequently make electron and/or hydrogen-atom donation to DPPH• less favorable. Accordingly, **E6** did not reach 50% DPPH inhibition within the concentration range investigated. The remaining compounds displayed IC_50_ values ranging from 110.18 to 251.91 μM.

In the ABTS assay, all synthesized derivatives exhibited IC_50_ values between 10.06 and 14.62 μM. Compound **E4** showed the highest activity (IC_50_ = 10.06 μM), while compound **E3** displayed the lowest activity (IC_50_ = 14.62 μM). For comparison, Trolox exhibited an IC_50_ value of 19.63 μM.

#### 2.2.2. Electron Transfer Assays

The electron transfer capacity of compounds **E1**–**E8** was evaluated using the TAC, RP, FRAP, and CUPRAC assays. The results are presented in Table 2 as molar equivalents (Eq) relative to the corresponding reference standards.

In the TAC assay, the obtained values ranged from 1.28 to 1.42 ascorbic acid molar equivalents/mol of tested compound, with compound **E2** exhibiting the highest activity. The RP assay showed greater variability, with compound **E5** displaying the highest reducing power (1.30 ascorbic acid molar equivalents/mol of tested compound), whereas compound **E8** exhibited the lowest activity (0.74 ascorbic acid molar equivalents/mol of tested compound). In the FRAP assay, compound **E1** exhibited the highest ferric-reducing capacity (1.10 Trolox equivalents/mol of tested compound), followed by compounds **E4**, **E2** and **E5**. In contrast, the CUPRAC assay identified compound **E6** as the most active derivative (2.39 Trolox molar equivalents/mol of tested compound), while the remaining compounds showed values between 2.05 and 2.20 molar equivalents of Trolox/mol of the tested compound.

### 2.3. Prediction of Antioxidant Activity by In Silico Methods

#### 2.3.1. Theoretical Quantum Calculation

As a continuation of the experimental antioxidant evaluation, density functional theory (DFT) calculations were performed for compounds **E1**–**E8**. The calculated HOMO and LUMO energies, HOMO–LUMO energy gaps, and dipole moments in vacuum, nonpolar, polar, and aqueous environments are summarized in Table 3 and Table 4.

**Figure 1:**
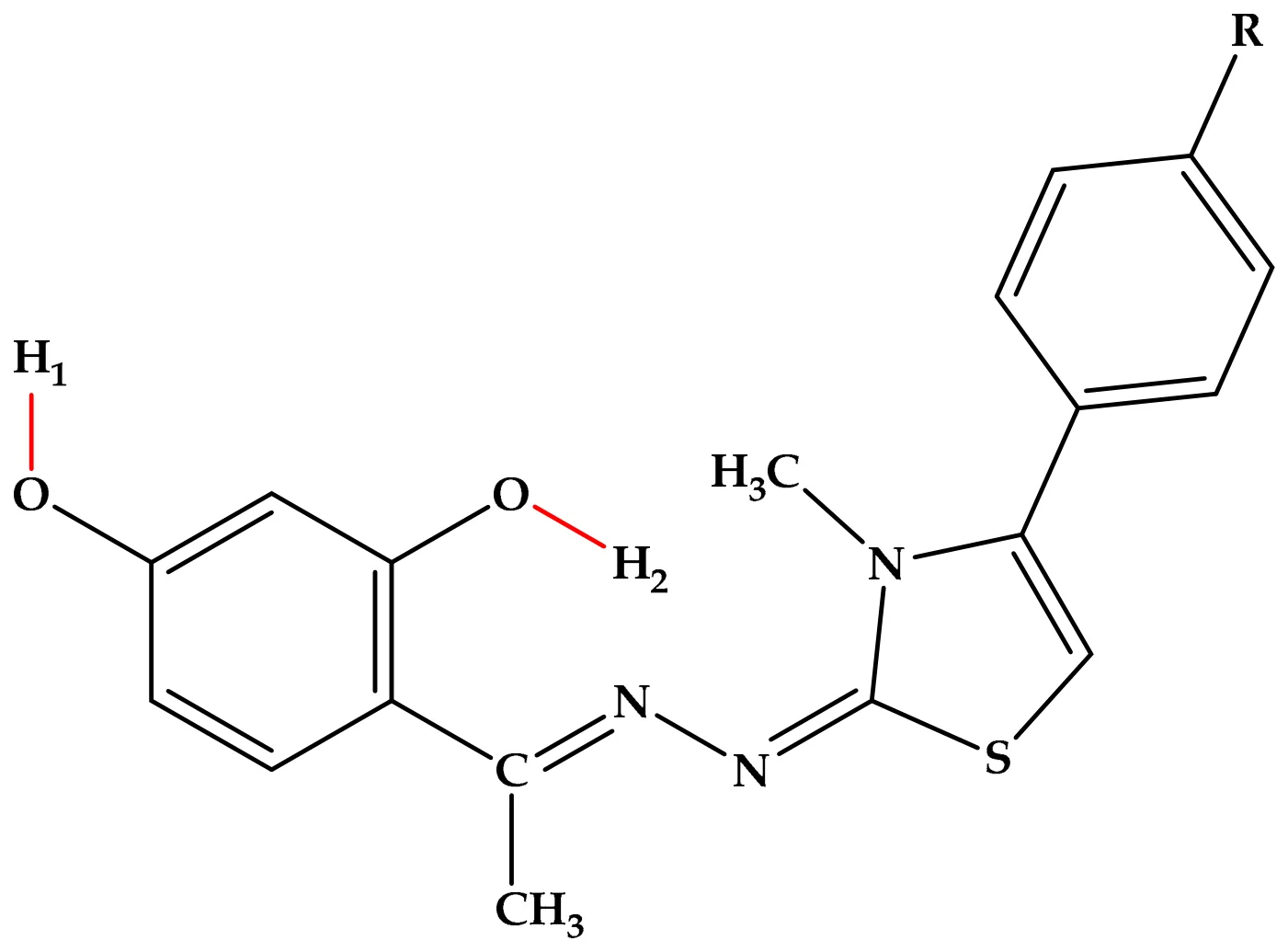
The general structure of the compounds and their potential hydrogen atom-donating sites (H_1_ and H_2_) capable of releasing hydrogen atoms to neutralize radicals.

In addition, the bond dissociation enthalpy (BDE) values of the phenolic O–H bonds, corresponding to the two potential hydrogen atom-donating sites (H1 and H2) shown in Figure 1, were calculated in the same media. The resulting BDE values are presented in Table 5.

#### 2.3.2. Prediction of Molecular Properties

The molecular properties of compounds **E1**–**E8** relevant to their pharmacokinetic behavior were predicted using the SwissADME platform. The calculated molecular descriptors, including molecular weight (MW), number of rotatable bonds, hydrogen bond acceptors (HBA), hydrogen bond donors (HBD), topological polar surface area (TPSA), MLogP, predicted water solubility, and compliance with Lipinski’s rule of five, are summarized in Table 6.

All compounds complied with Lipinski’s rule of five, as no violations were identified for any of the investigated derivatives. The molecular weights of the investigated compounds ranged from 339.41 to 407.41 g/mol, remaining below the Lipinski threshold of 500 g/mol. Similarly, all compounds exhibited suitable hydrogen bonding characteristics, with the number of hydrogen bond acceptors varying between 4 and 7 and the number of hydrogen bond donors remaining constant at 2 for the entire series.

The number of rotatable bonds ranged from 3 to 4, indicating relatively moderate conformational flexibility. Compounds **E5**, **E6**, and **E7** exhibited four rotatable bonds due to the presence of bulkier substituents, whereas the remaining derivatives possessed three rotatable bonds.

Most compounds displayed TPSA values around 98 Å^2^, which generally fall within the range associated with good intestinal absorption. However, compounds **E6** and **E8** exhibited markedly higher TPSA values 144.17 and 122.14 Å^2^, respectively, reflecting the presence of strongly polar nitro and cyan substituents.

Lipophilicity analysis showed moderate MLogP values for most compounds, ranging from 1.48 to 3.92, indicating a balanced hydrophilic–lipophilic profile. Compound **E7** exhibited the highest lipophilicity (MLogP = 5.66), likely due to the strongly hydrophobic trifluoromethyl substituent. On the other hand, compound **E3** displayed the lowest MLogP value (1.48), suggesting a more hydrophilic character associated with the chloro substituent and overall electronic distribution within the molecule.

The predicted water solubility values also varied considerably throughout the series. Compounds **E6** and **E8** had the highest aqueous solubility, whereas **E3** and **E7** exhibited the lowest solubility values. The observed solubility differences appear to correlate with both lipophilicity and molecular polarity. ADME predictions indicated high gastrointestinal absorption for all derivatives except **E6**, while none of the compounds were predicted to cross the blood–brain barrier.

### 2.4. Cytotoxicity Evaluation

**Figure 2:**
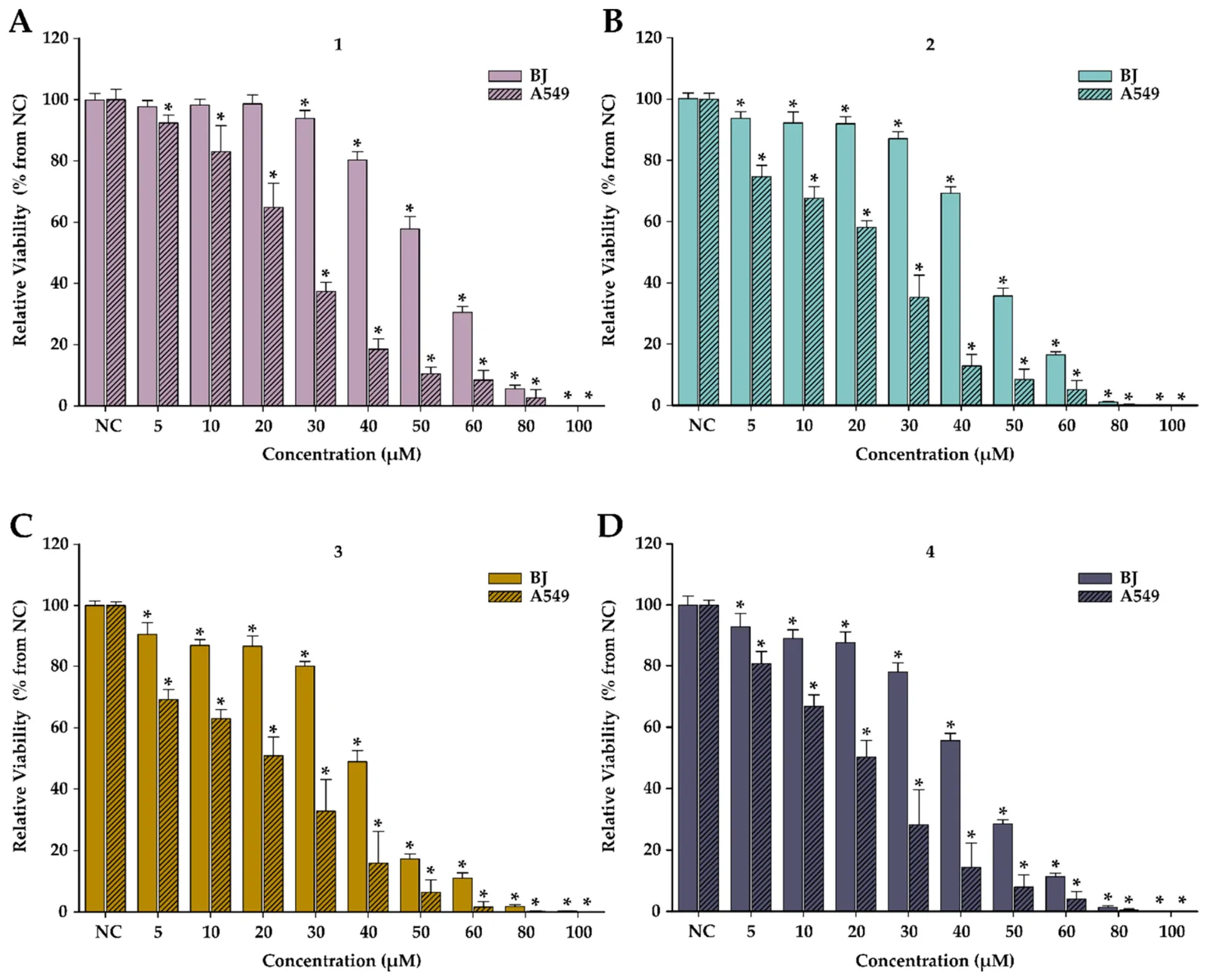
Cytotoxic effect of **E1** (**A**), **E2** (**B**), **E3** (**C**), **E4** (**D**) after 48 h exposure of BJ and A549 cells. The results are presented as relative means ± standard deviations of three biological replicates. Data were expressed as relative values compared to the vehicle negative control (NC) (100%). (*) indicate significant differences (*p* < 0.05) compared to NC.

**Figure 3:**
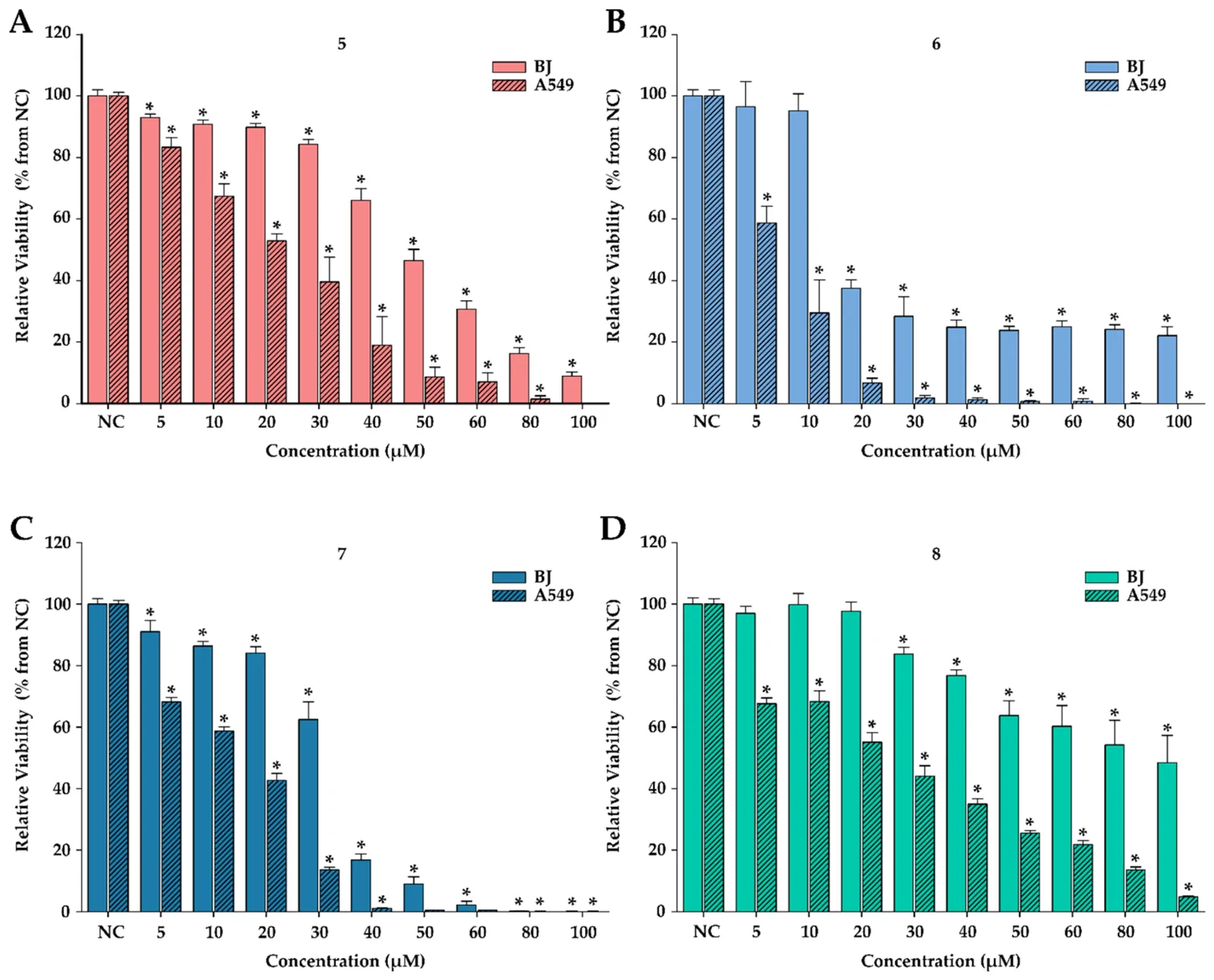
Cytotoxic effect of **E5** (**A**), **E6** (**B**), **E7** (**C**), **E8** (**D**) after 48 h exposure of BJ and A549 cells. The results are represented as relative means ± standard deviations of three biological replicates. Data were expressed as relative values compared to the vehicle negative control (NC) (100%). (*) indicate significant differences (*p* < 0.05) compared to NC.

The cytotoxic activity of compounds **E1**–**E8** was evaluated against human lung adenocarcinoma A549 cells and normal human fibroblast BJ cells following 48 h of exposure. Prior to cytotoxicity evaluation, the interference of the synthesized compounds with the resazurin-based assay was evaluated. No relevant intrinsic fluorescence was detected, while incubation of the compounds with resazurin or resorufin at the highest tested concentration did not significantly alter the analytical signal. These results indicate that neither intrinsic fluorescence nor direct redox interactions with the assay components measurably affected the fluorimetric measurements under the experimental conditions. Cell viability profiles at the tested concentrations are presented in Figure 2 and Figure 3, while the corresponding IC_50_ values and selectivity indices, calculated as the ratio IC_50_(BJ)/IC_50_(A549), are summarized in Table 7. Among the investigated derivatives, compound **E6** displayed the highest cytotoxic activity against A549 cells, with an IC_50_ value of 5.97 μM, followed by compound **E7** (13.99 μM). The remaining derivatives exhibited IC_50_ values between 20.78 and 27.62 μM. Gefitinib, used as the reference compound, showed an IC_50_ value of 15.93 μM under the same experimental conditions.

The highest selectivity index was also observed for compound **E6** (2.64), followed by **E7** (2.36), whereas the remaining derivatives showed selectivity indices ranging from 1.46 to 2.07. All compounds exhibited lower toxicity toward normal BJ fibroblasts, resulting in selectivity index values above 1 for the entire series. These findings indicate a certain degree of selectivity toward malignant cells. All synthesized compounds reduced the viability of both cell lines in a concentration-dependent manner.

## 3. Discussion

### 3.1. Synthetic Approach and Structural Characterization of Polyphenolic–Methyliden–Hydrazono–Thiazole Derivatives

All synthesized compounds were isolated as yellow-to-orange crystalline solids and stored in tightly sealed amber vials to minimize oxidation and ensure long-term stability. Their molecular structures were established by complementary spectroscopic techniques, including mass spectrometry (MS), FT-IR spectroscopy, and ^1^H/^13^C NMR spectroscopy. The corresponding spectra are provided in the Supplementary Materials (Figures S1–S32).

Mass spectrometry analysis revealed the expected molecular ion peaks for all synthesized compounds, **E1**–**E8** (Figures S25–S32). The MS/MS fragmentation patterns provided additional confirmation of the proposed structures. For all compounds, fragmentation of the precursor ion generated two characteristic product ions, consistent with cleavage of the N–N hydrazone bond. A common fragment ion was observed at approximately *m*/*z* 150 and was assigned to the 4-(1-iminoethyl)benzene-1,3-diol moiety. A second characteristic fragment, with *m*/*z* values ranging from 189.0479 to 259.0515 depending on the substituent, was assigned to the 3-methyl-4-phenylthiazol-2(3H)-imine-derived moiety. The ^1^H NMR spectra (Figures S9–S16) were fully consistent with the proposed thiazolyl–polyphenolic structures. The H-5 proton of the thiazole ring appeared as a characteristic singlet at δ 6.3–6.7 ppm, while aromatic proton resonances were observed within the expected δ 6.0–8.0 ppm region. The 2,4-dihydroxyphenyl hydrazone fragment exhibited the expected aromatic spin system (doublet of doublets at around 6.3 ppm (*J* = 2.5 and 8.5 Hz), accompanied by two additional aromatic signals located near 6.2 and 7.4 ppm). In the aliphatic region, all spectra showed a singlet at approximately 3.5 ppm, attributable to the N-methyl group of the thiazole nucleus. Compounds bearing methoxy or methyl substituents on the aromatic ring exhibited additional singlet resonances at approximately 3.3 and 2.3 ppm, respectively, corresponding to the OCH_3_ and CH_3_ groups.

The ^13^C NMR spectra (Supplementary Figures S17–S24) further confirmed the proposed structures, displaying the expected resonances for aromatic, thiazole, imine, and aliphatic carbon atoms. The aromatic carbon resonances were observed within the expected range of 98–166 ppm, including signals corresponding to both protonated and substituted aromatic carbons. Two characteristic aliphatic carbon signals, present in all derivatives, appeared at approximately 14 and 34 ppm and were assigned to the methyl groups incorporated within the molecular scaffold. The methoxy- and methyl-substituted derivatives showed the additional carbon signals characteristic of these substituents in the 21–55 ppm region, consistent with the presence of these functionalities.

The FT-IR spectra (Supplementary Figures S1–S8) exhibited the characteristic absorption bands expected for the synthesized compounds, including broad phenolic O–H stretching vibrations (3000–3200 cm^−1^), C=N stretching bands around 1600 cm^−1^, and C–N stretching bands near 1200 cm^−1^. Additional absorption bands corresponding to the methoxy, nitro, cyano, trifluoromethyl, chloro, and fluoro substituents were observed in the appropriate derivatives, further supporting their structural assignment.

RP-HPLC analysis showed a single predominant chromatographic peak for each compound and was added as supporting evidence of chromatographic purity.

The MS, FT-IR, and NMR data were in full agreement with the proposed molecular structures, confirming the successful synthesis of the new thiazolyl–polyphenolic derivatives.

### 3.2. In Vitro Assessment of Antioxidant Activity

Oxidative stress plays a central role in the onset and progression of numerous pathological conditions, including cancer, cardiovascular diseases, neurodegenerative disorders, and chronic inflammatory diseases. Consequently, the development of compounds capable of efficiently neutralizing reactive oxygen species through different antioxidant mechanisms remains an important objective in medicinal chemistry. To obtain a comprehensive assessment of the antioxidant potential of the synthesized thiazolyl–polyphenolic derivatives, complementary assays based on hydrogen atom transfer (HAT) and electron transfer (ET) mechanisms were employed. Because the antioxidant behavior of phenolic compounds depends on multiple reaction pathways, the combined use of DPPH, ABTS, TAC, RP, FRAP, and CUPRAC assays provides a more reliable evaluation than any individual method alone [22,23].

#### 3.2.1. Antiradical Assays

The activity of compounds **E1**–**E8** was evaluated using the DPPH^•^ and ABTS^•+^ radical scavenging assays and the results revealed noticeable differences between compounds, depending on the nature of the substituent attached to the aromatic ring. Overall, the investigated derivatives exhibited variable radical scavenging capacities, indicating that the electronic properties of the substituents significantly influence their antioxidant behavior.

Although both DPPH^•^ and ABTS^•+^ assays are widely employed to evaluate radical scavenging activity, they differ in their reaction mechanisms, sensitivity to steric effects, and solvent compatibility. Consequently, the use of both assays provides complementary information regarding the antioxidant behavior of the synthesized derivatives [23].

The DPPH˙ (2,2-diphenyl-1-picrylhydrazyl) radical scavenging assay, originally described by Brand-Williams et al., is based on the transfer of a hydrogen atom from the antioxidant under investigation to the stable violet DPPH˙ radical, resulting in the formation of a nonradical, stable yellow compound. The decrease in absorbance at 517 nm reflects the amount of radical neutralized [24].

As shown in Table 1, the investigated thiazolyl–polyphenolic derivatives exhibited distinct radical scavenging profiles depending on the nature of the aromatic substituent. None of the synthesized derivatives reached the radical scavenging activity of the reference antioxidants. The best compound **E5** exhibited an IC_50_ value approximately threefold higher than that of Trolox (Table 1). Considering the structural novelty of these thiazolyl–polyphenolic hybrids, this level of activity may be regarded as promising and suggests that further structural optimization could improve their antioxidant potential. In the DPPH assay, compounds bearing electron-donating substituents demonstrated superior activity, with the methoxy derivative **E5** exhibiting the lowest IC_50_ value among the synthesized compounds, followed by the methyl analogue **E2**. In contrast, derivatives containing strongly electron-withdrawing substituents, particularly **E7** (CF_3_), **E8** (CN), and **E6** (NO_2_), displayed markedly reduced activity.

The lowest activities were observed for **E7** (-CF_3_) and **E8** (-CN), suggesting that the presence of electron-withdrawing groups decreases the electron-donating ability of the molecules and reduces the stability of the resulting antioxidant radical intermediates. The cyano group exerts both strong inductive and mesomeric electron-withdrawing effects, which likely account for the markedly diminished activity of compound **E8**. Similarly, the trifluoromethyl substituent strongly decreases the electron density of the aromatic system, negatively affecting the hydrogen atom transfer process involved in DPPH radical neutralization. Compound **E6**, bearing a nitro substituent, did not provide a measurable DPPH activity under the experimental conditions. This behavior may be explained by the strong electron-withdrawing nature of the nitro group, which significantly reduces the ability of the molecule to donate electrons or hydrogen atoms. Additionally, nitro-containing aromatic systems may undergo secondary redox processes or exhibit slower reaction kinetics toward the DPPH radical, which could interfere with the assay response.

The atypical behavior of **E6** is likely multifactorial. In addition to the strong electron-withdrawing effect of the nitro group, which may reduce electron- and hydrogen-donating ability, its high polarity and ionization state may influence various parameters, such as solubility, which were not directly investigated; thus they should be regarded as plausible contributors.

Compared with the DPPH^•^ assay, the ABTS^•+^ assay produced a narrower range of IC_50_ values (Table 1), indicating that all synthesized compounds showed similar radical scavenging activity. All synthesized compounds exhibited lower IC_50_ values than Trolox in the ABTS•+ assay (Table 1), indicating a higher radical scavenging efficiency under these experimental conditions. This observation further supports the contribution of the common dihydroxylated aromatic scaffold to the electron transfer capacity of the investigated derivatives. In this assay, the influence of the aromatic substituent was less evident, suggesting that the antioxidant activity mainly depends on the common 2,4-dihydroxylated aromatic moiety present in all compounds. This structural fragment contains two phenolic hydroxyl groups that can donate hydrogen atoms and stabilize the resulting phenoxyl radicals through resonance.

The highest ABTS^•+^ activity was observed for compounds **E4**, **E5**, and **E7** (Table 1). Interestingly, although **E7** showed weak activity in the DPPH assay, it was among the most active compounds in the ABTS^•+^ assay. This difference can be explained by the different reaction mechanisms involved in the two methods. The DPPH^•^ assay mainly reflects the hydrogen atom transfer mechanism and is more affected by steric factors, whereas the ABTS^•+^ assay can involve both hydrogen atom transfer and electron transfer processes and is generally less influenced by solubility effects. Therefore, compounds containing electron-withdrawing substituents may still exhibit good activity in the ABTS^•+^ assay despite their lower hydrogen-donating ability.

#### 3.2.2. Electron Transfer Assays

The electron transfer capacity of compounds **E1**–**E8** was further evaluated using four complementary assays, namely TAC, RP, FRAP, and CUPRAC. Although all four methods are based on electron transfer reactions, they differ in their oxidizing systems and experimental conditions, providing complementary information on the reducing ability of the synthesized derivatives. The results obtained in these assays are summarized in Table 2.

Overall, all compounds showed moderate to good electron transfer activity, indicating that the polyphenolic scaffold retained its reducing ability after incorporation of the thiazole ring. However, noticeable differences were observed depending on both the nature of the aromatic substituent and the assay employed.

In the TAC assay, all compounds showed relatively similar activities, with values ranging from 1.28 to 1.42 ascorbic acid equivalents/mol of tested compound. Compounds **E2** and **E6** gave the highest values, followed by **E4**, **E7**, and **E8**. The small differences observed suggest that the common 2,4-dihydroxyphenyl moiety is mainly responsible for reducing activity measured by this assay. The enhanced activity of **E2** may be attributed to the electron-donating effect of the methyl substituent, which increases the electron density within the conjugated system and facilitates electron transfer processes. Compound **E6**, containing a strongly electron-withdrawing nitro group, also exhibited elevated activity, suggesting that additional resonance-assisted redox processes may contribute to the antioxidant response under TAC assay conditions. In addition, all synthesized compounds showed TAC values higher than ascorbic acid, indicating good total antioxidant capacity.

More pronounced differences were observed in the RP assay (Table 2). Compound **E5** exhibited the highest reducing power (1.30 Eq ascorbic acid/mol of the tested compound), followed by compounds **E2** and **E6.** The superior reducing capacity of **E5** can be associated with the strong electron-donating effect of the methoxy substituent, which enhances electron delocalization and promotes reduction processes. Likewise, the methyl substituent in **E2** positively contributes through its inductive electron-donating effect. In contrast, compound **E8**, bearing a cyano group, showed the lowest reducing power, reflecting the electron-withdrawing nature of this substituent.

The reducing power of **E5** exceeded the reference antioxidant, further highlighting the beneficial effect of methoxy substitution on electron transfer processes.

The FRAP assay revealed a different antioxidant profile (Table 2). In this method, compounds **E1** and **E4** exhibited the highest ferric reducing capacities, while compound **E6** displayed the lowest activity. Since the FRAP assay is performed under acidic conditions, the measured reducing capacity may be significantly influenced by ionization of the phenolic groups as well as by substituent-dependent electronic effects, which could explain the different ranking observed compared with the RP assay. The lower activity of **E6** suggests that the nitro substituent may negatively affect ferric ion reduction under acidic conditions despite its favourable performance in other electron transfer assays.

Among all investigated methods, the CUPRAC assay produced the highest overall antioxidant values, ranging from 2.05 to 2.39 Eq Trolox/mol of tested compound (Table 2). Compound **E6** exhibited the highest cupric ion reducing capacity, followed by **E7**, whereas compounds **E1**, **E3**, and **E5** displayed comparable activities. The superior activity of **E6** could be explained by the ability of the nitro substituent to stabilize charge-transfer intermediates formed during the reduction process. Unlike the FRAP assay, the CUPRAC method is performed under nearly physiological conditions and is less affected by steric and solubility limitations, allowing a broader evaluation of the reducing capacity of multifunctional phenolic compounds.

Taken together, the results obtained from the four electron transfer assays indicate that the antioxidant behavior of the synthesized derivatives is influenced by both the electronic properties of the aromatic substituents and the experimental conditions of each assay. While the common 2,4-dihydroxylated aromatic moiety provides the main redox-active framework, the aromatic substituent modulates the efficiency of electron transfer depending on the oxidizing system employed.

### 3.3. Prediction of Antioxidant Activity by In Silico Methods

#### 3.3.1. Theoretical Quantum Calculation

To better understand the experimental antioxidant results, density functional theory (DFT) calculations were performed for compounds **E1**–**E8** as neutral free bases. The calculated HOMO and LUMO energies, HOMO–LUMO energy gaps, dipole moments, and bond dissociation enthalpies (BDEs) are presented in Table 3, Table 4 and Table 5.

Frontier molecular orbitals are key parameters for predicting the chemical reactivity of a compound and were therefore investigated in this study. The highest occupied molecular orbital (HOMO) is related to the electron-donating ability of the molecule and its susceptibility to electrophilic attack, while the lowest unoccupied molecular orbital (LUMO) reflects the electron-accepting capacity and vulnerability to nucleophilic attack. Previous studies have indicated that the HOMO–LUMO energy gap may correlate with the antioxidant potential of compounds. Accordingly, the energies of the HOMO and LUMO orbitals, together with the HOMO–LUMO energy gap, were calculated, and the obtained values are summarized in Table 3 and Table 4.

The calculated HOMO energies ranged from −5.13 to −5.54 eV (Table 3). Compounds **E2** and **E5**, containing methyl and methoxy substituents, exhibited the highest HOMO energy values, whereas compounds bearing strong electron-withdrawing groups, particularly **E6** (-NO_2_) and **E8** (-CN), showed the lowest values. Compound **E7** (-CF_3_) displayed intermediate behavior, while the halogenated derivatives **E3** (-Cl) and **E4** (-F) showed HOMO energies close to those of the unsubstituted compound. These results are consistent with the experimental DPPH assay, where compounds **E2** and **E5** exhibited the highest radical scavenging activity, whereas **E6, E7**, and **E8** were considerably less active.

The HOMO–LUMO energy gaps also depended on the nature of the aromatic substituent (Table 3). Compounds **E1**–**E5** presented similar energy gaps (3.77–3.82 eV), indicating comparable electronic stability. The relatively high energy gaps indicate that these compounds maintain a balanced electronic structure despite the presence of different substituents. In contrast, compound **E6** exhibited the lowest HOMO–LUMO gap in all investigated media, while **E8** (CN) showed slightly higher values.

An important structural aspect contributing to the electronic behavior of all compounds is the presence of the aromatic moiety containing two hydroxyl groups. These hydroxyl groups can participate in resonance stabilization and facilitate electron delocalization throughout the conjugated hydrazone-thiazole system. The extended π-conjugation between the aromatic ring, azomethine linkage, and heterocyclic fragment contributes to the stabilization of the molecular electronic structure and may favour radical stabilization after electron or hydrogen atom transfer.

The solvent had only a minor influence on the calculated orbital energies, suggesting that the electronic properties of the synthesized compounds are mainly determined by their molecular structure rather than by the surrounding medium.

The calculated dipole moments (Table 4) provide additional information regarding molecular polarity, which may influence solubility and intermolecular interactions under biological conditions. As expected, the dipole moment increased from a vacuum to the aqueous medium for all compounds, reflecting the higher polarization of the molecules in polar environments. The highest dipole moments were observed for compounds **E2** and **E5**, while **E7** showed the lowest values. Compounds **E6** and **E8** exhibited intermediate polarity despite the presence of strongly electron-withdrawing substituents. Overall, these results indicate that both the aromatic substituent and the solvent polarity influence the electronic distribution within the synthesized molecules.

Among the calculated descriptors, the BDE values showed the best agreement with the experimental radical scavenging activity.

The calculated BDE values of the phenolic O–H bonds are summarized in Table 5. Since lower BDE values indicate easier hydrogen atom donation, this parameter is considered a useful descriptor of antioxidant activity based on the hydrogen atom transfer mechanism. For all investigated compounds, the O–H_1_ bond showed consistently lower BDE values than the O–H_2_ bond, indicating that O–H_1_ represents the preferred hydrogen-donating site. The difference of approximately 7–9 kcal/mol between O–H_1_ and O–H_2_ indicates a clear preference for radical stabilization through the O–H_1_ position.

The obtained BDE values also show a noticeable solvent effect. A gradual decrease in BDE values was observed from vacuum to aqueous medium for all compounds, indicating that polar solvents favour hydrogen atom transfer by stabilizing the generated phenoxyl radicals. Among the investigated derivatives, compound **E5** exhibited the lowest BDE values in all media (79.31 kcal/mol in vacuum and 75.14 kcal/mol in water), in agreement with its superior DPPH radical scavenging activity. In contrast, compounds **E6 (NO2)**, **E7 (CF3)**, and **E8 (CN)** showed higher BDE values, consistent with their weaker antioxidant activity observed experimentally.

Overall, the DFT calculations showed good agreement with the experimental antioxidant results. Compounds with higher HOMO energies and lower BDE values generally exhibited stronger radical scavenging activity, whereas derivatives containing strong electron-withdrawing substituents presented lower electron-donating ability and less favourable hydrogen atom transfer. For the studied compounds, the calculated BDE values should be regarded as indicators of the relative propensity of the O–H bonds in the parent molecules to undergo the initial hydrogen-atom abstraction rather than as quantitative contributions of individual hydroxyl groups to the overall antioxidant activity, since subsequent H-abstraction steps involve radical intermediates with altered electronic structures. These findings support the important role of the electronic nature of the aromatic substituent in modulating the antioxidant properties of the synthesized thiazolyl–polyphenolic derivatives.

#### 3.3.2. Prediction of Molecular Properties

The physicochemical properties of compounds **E1**–**E8** were evaluated using the SwissADME platform to estimate their drug-likeness and potential pharmacokinetic behavior. The calculated molecular descriptors are summarized in Table 6.

All synthesized compounds complied with Lipinski’s rule of five, with no violations observed, suggesting general compliance with conventional drug-likeness criteria. Their molecular weights remained below 500 g/mol, while the number of hydrogen bond donors and acceptors were within the recommended limits for orally active compounds.

The calculated TPSA values ranged from approximately 98 Å^2^ for most derivatives to 144.17 Å^2^ for compound **E6** (Table 6). Increased TPSA values are commonly associated with enhanced polarity and hydrogen bonding capacity but may negatively influence passive membrane permeability.

Although compounds **E6** and **E8** exhibited higher polarity due to the presence of nitro and cyano substituents, their TPSA values remained close to the range generally considered compatible with intestinal absorption.

Most compounds displayed moderate lipophilicity, with MLogP values between 1.48 and 3.92, indicating a balanced hydrophilic–lipophilic profile. Compound **E7** showed the highest lipophilicity (MLogP = 5.66), reflecting the presence of the trifluoromethyl group, whereas **E3** exhibited the lowest value. The predicted aqueous solubility generally followed the same trend, with the more lipophilic derivatives showing lower solubility.

None of the compounds were predicted to cross the blood–brain barrier, suggesting limited central nervous system exposure. From the perspective of peripheral antitumor activity, this may not necessarily represent a disadvantage, but it confirms that the current scaffold is unlikely to be suitable for indications requiring significant CNS penetration. Overall, these results show that fulfillment of Lipinski’s criteria alone does not guarantee an optimal pharmacokinetic profile. Future structural optimization should therefore aim to balance polarity, lipophilicity, aqueous solubility, and membrane permeability in order to improve the oral drug-like properties of this series.

Overall, the calculated molecular descriptors indicate that the synthesized thiazolyl–polyphenolic derivatives possess favourable physicochemical properties that support their further investigation as potential bioactive compounds.

### 3.4. Cytotoxicity Evaluation

The cytotoxic activity of compounds **E1**–**E8** was evaluated against the human lung adenocarcinoma cell line A549 and the normal human fibroblast cell line BJ following 48 h exposure. The obtained IC_50_ values and selectivity indices (SI) are presented in Table 7. Gefitinib was used as the reference anticancer agent chosen from the European Society for Medical Oncology (ESMO) guidelines, which recommend this tyrosine kinase inhibitor as a first-line treatment for patients with EGFR-mutated non-small cell lung cancer (NSCLC). The selection was further justified by previous reports demonstrating that structurally related 4-substituted thiazole derivatives exert anticancer effects through EGFR inhibition, the same molecular mechanism underlying the therapeutic activity of gefitinib. In the present study, gefitinib was included solely as a reference compound for evaluating the cytotoxic activity, since it has no established intrinsic antioxidant or redox-related effects [25,26,27].

All investigated compounds demonstrated cytotoxic activity against A549 cells, with IC_50_ values ranging from 5.97 to 27.62 μM. At the same time, the compounds exhibited lower toxicity toward normal BJ fibroblasts, resulting in selectivity index values above 1 for the entire series. These findings indicate a certain degree of selectivity toward malignant cells.

Among the tested derivatives, compound **E6** exhibited the strongest antiproliferative activity against A549 cells, with an IC_50_ value of 5.97 μM, higher than the reference drug gefitinib which had an IC_50_ value of 15.93 μM. Moreover, **E6** also displayed the highest selectivity index (SI = 2.64), indicating superior selectivity toward cancer cells compared to normal fibroblasts. Compound **E7** also demonstrated notable cytotoxic activity, with an IC_50_ value of 13.99 μM and a selectivity index of 2.36, surpassing gefitinib in terms of selectivity.

The remarkable activity observed for **E6** and **E7** suggests that strongly electron-withdrawing substituents, such as nitro and trifluoromethyl groups, may positively influence the antiproliferative properties of the investigated derivatives. These substituents can significantly modify the electronic distribution, lipophilicity, and cellular permeability of the molecules, potentially enhancing their interaction with intracellular biological targets. In particular, the nitro group present in **E6** may participate in intracellular redox processes capable of generating reactive intermediates or inducing oxidative stress selectively in tumor cells.

Compounds **E1**, **E3**, and **E8** demonstrated moderate cytotoxic effects against A549 cells, with IC_50_ values between approximately 25 and 28 μM. Their selectivity indices ranged from 1.46 to 2.07, indicating selectivity toward tumor cells.

Overall, the obtained results indicate that compounds **E6** and **E7** represent the most promising derivatives within the investigated series, combining potent antiproliferative activity with favourable selectivity toward cancer cells. These findings support further investigation of their molecular mechanisms of action and potential therapeutic applications.

### 3.5. Limitations of the Study

The antioxidant activity was evaluated using in vitro chemical assays, which provide useful comparative information but cannot fully reproduce the complexity of cellular redox processes. Although the cytotoxicity results identified compounds **E6** and **E7** as the most promising derivatives, the antiproliferative evaluation was limited to a single cancer cell line (A549) and one normal fibroblast cell line (BJ), and the underlying molecular mechanisms of cytotoxicity were not experimentally investigated. In addition, the DFT and ADME analyses provide theoretical support for the observed structure–activity relationships, but they cannot substitute for experimental pharmacokinetic, permeability, metabolic stability, and in vivo studies. The calculated BDE values describe the initial hydrogen-atom abstraction from the parent molecules and do not quantify the sequential contribution of individual hydroxyl groups to the overall antioxidant response. Moreover, the present series includes only eight compounds; therefore, the proposed relationships between substituent electronic effects and biological activity should be regarded as preliminary structure–activity information.

## 4. Materials and Methods

### 4.1. Materials: Chemicals, Reagents and Equipment

All reagents and solvents used for the synthesis, purification, structural characterization, and biological evaluation of compounds **E1**–**E8** were of analytical grade and were used without further purification unless otherwise stated. Methyl isothiocyanate, hydrazine monohydrate (98%), 2′,4′-dihydroxyacetophenone, and the corresponding α-haloketones employed for the synthesis of the target compounds were purchased from Sigma-Aldrich (St. Louis, MO, USA), Merck (Merck KGaA, Darmstadt, Germany), Alfa Aesar (Thermo Fisher Scientific, Waltham, MA, USA), or TCI (Tokyo Chemical Industry UK Ltd., Oxford, UK). Dimethyl sulfoxide (DMSO, ≥99%) used for the preparation of stock solutions was obtained from Merck (Merck KGaA, Darmstadt, Germany).

The synthesized compounds were characterized by melting point determination, high-resolution mass spectrometry (HRMS), Fourier-transform infrared spectroscopy (FT-IR), and nuclear magnetic resonance (NMR) spectroscopy. Melting points were determined using an MPM-H1 melting point apparatus (Schorpp Gerätetechnik, Überlingen, Germany) employing the capillary tube method and are reported uncorrected.

The purity of the final compounds **E1**–**E8** was assessed by RP-HPLC on a Shimadzu Nexera X3 HPLC system (Shimadzu Corporation, Kyoto, Japan) equipped with a Luna C18 analytical column (150 × 4.6 mm i.d., 5 μm particle size, 100 Å pore size, Phenomenex, Danaher, Washington, DC, USA) at 40 °C, with isocratic elution using water/acetonitrile (25:75, *v*/*v*) at a flow rate of 0.7 mL/min and UV detection at 254 nm using a SPD-M40 detector (Shimadzu Corporation, Kyoto, Japan).

Mass spectra were recorded in positive ionization mode using a Shimadzu QTOF 9030 mass spectrometer, where data acquisition and data processing were made using LabSolutions software v5.71 SP2 (Shimadzu Corporation, Kyoto, Japan). FT-IR spectra were acquired on an FT/IR-6100 spectrometer (Jasco, Cremella, Italy) using KBr pellets prepared under vacuum.

^1^H and ^13^C NMR spectra were recorded on a Bruker Avance spectrometer (Bruker, Karlsruhe, Germany). Chemical shifts (δ) are reported in parts per million (ppm) relative to the residual solvent signal of DMSO-*d*_6_, while tetramethylsilane (TMS) was used for calibration. The obtained spectra were consistent with the proposed structures of the synthesized compounds. Signal multiplicities are designated as follows: s (singlet), d (doublet), dd (doublet of doublets), t (triplet), and m (multiplet). The abbreviations Ar, Bzd, and Th refer to the aromatic substituent at position 4 of the thiazole ring, the benzylidene fragment, and the thiazole ring, respectively.

For antioxidant assays, absorbance measurements were performed using a Specord 210 PLUS UV–Vis spectrophotometer (Analytik Jena AG, Jena, Germany) equipped with disposable low-volume 10 mm optical pathlength cuvettes.

Density functional theory (DFT) calculations were carried out on a workstation equipped with an Intel^®^ Core™ i7-12700KF processor operating under Windows 10 (Microsoft Corporation, Redmond, WA, USA).

### 4.2. Synthetic Approach and Structural Characterization of Polyphenolic–Methyliden–Hydrazono–Thiazole Derivatives

The target thiazolyl–polyphenolic derivatives **E1**–**E8** were synthesized according to the synthetic pathway outlined in Scheme 1. The synthesis commenced with the preparation of 4-methyl-3-thiosemicarbazide **(3)** through the reaction of methyl isothiocyanate **(1)** with hydrazine monohydrate **(2)** in ethanol. Then 45 mmol of methyl isothiocyanate **(1)** was dissolved in 10 mL of 96% ethanol, and 45 mmol of 98% hydrazine monohydrate **(2)** was added dropwise under cold conditions with continuous stirring. The reaction, which proceeded rapidly, was maintained for 0.5 h to ensure completion. The resulting precipitate of 4-methyl-3-thiosemicarbazide **(3)** was collected by vacuum filtration, purified by washing with 96% ethanol, and stored for subsequent use. The reaction progress was monitored by thin-layer chromatography (TLC) using an ethyl acetate/heptane (1:1, *v*/*v*) solvent system, and the yield was 81% [28,29].

The key thiosemicarbazone intermediate **(5)** was subsequently prepared by condensation of 4-methyl-3-thiosemicarbazide **(3)** with 2′,4′-dihydroxyacetophenone **(4)** in aqueous acetic acid under reflux. Then 20 mmol of 4-methyl-3-thiosemicarbazide **(3)**, dissolved in 10 mL of the 10% acetic acid solution, was treated with 20 mmol of 2′,4′-dihydroxyacetophenone **(4)** and refluxed with continuous stirring for 3 h. Following completion of the reaction the resulting precipitate was isolated by hot filtration and dried with anhydrous MgSO_4_ for 3 h in vacuum, prior to further use [28,29,30].

Cyclization of intermediate **(5)** with the appropriate α-haloketones **(6)** in refluxing acetone afforded the desired thiazole derivatives, **E1**–**E8**. For this step, 2 mmol of the dried thiosemicarbazone **(5)** was dissolved in 3 mL of acetone, and 2 mmol of various alpha-haloketones **(6)** was added. The resulting mixtures were refluxed for 2 h. Reaction progress was monitored by TLC, and the final products were isolated by hot filtration in order to minimize contamination with impurities that become insoluble upon cooling and purified by washing with fresh acetone. The synthetic sequence proceeded through a Hantzsch-type thiazole ring formation, yielding the corresponding thiazolyl–polyphenolic derivatives in moderate to good yields (56–77%) [8,30].

All target compounds were isolated as hydrobromide salts, which exhibited improved stability during storage and adequate solubility in the media employed for the antioxidant and cytotoxicity studies. The structures of the synthesized compounds were established by MS, FT-IR, and ^1^H/^13^C NMR spectroscopy. The obtained analytical and spectroscopic data were fully consistent with the proposed molecular structures. Intermediates **(3)** and **(5)** were previously reported in the literature [31,32]. Final compounds E1-E8 share structural similarities with ones reported by Yamazaki [33] but are original and not reported in the literature.

*4-((1E)-1-((3-methyl-4-phenylthiazol-2(3H)-ylidene)hydrazineylidene)ethyl)benzene-1,3-diol hydrobromide* (**E1**): yellow solid; mp = 235 °C; yield = 61%; FT-IR (KBr), νmax (cm^−1^): 1275 (N-CH_3_), 1620 (C=N), 3100, 3148 (phenolic OH); HRMS: *m*/*z* = 340.1114 (M_free neutral base_+1); ^1^H NMR (DMSO-*d*_6_, 500 MHz) δ: 2.47 (*s*, 3H, Bzd-CH_3_), 3.34 (*s*, 3H, -CH_3_), 6.29 (*d*, 1H, Bzd, *J* = 2 Hz), 6.34 (*dd*, 1H, Bzd, *J*_1_ = 2.5 Hz, *J*_2_ = 8.5 Hz), 6.45 (*s*, 1H, Th), 7.41 (*d*, 1H, Bzd, *J* = 8.5 Hz), 7.50–7.53 (*m*, 5H, Ar); ^13^C NMR (DMSO-*d*_6_, 125 MHz) δ: 14.4 (-CH_3_), 34.0 (-CH_3_), 98.9 (Th-C_5_), 103.2 (Bzd), 107.4 (Bzd), 112.4 (Bzd), 129.2 (Ar), 129.3 (Ar), 130.0 (Bzd), 130.8 (Ar), 141.5 (Th-C_4_), 160.2 (Bzd), 160.7 (Th-C_2_), 161.0 (Bzd-OH), 166.2 (Bzd-OH).

*4-((1E)-1-((3-methyl-4-(p-tolyl)thiazol-2(3H)-ylidene)hydrazineylidene)ethyl)benzene-1,3-diol hydrobromide* (**E2**): yellow solid; mp = 256 °C; yield = 64%; FT-IR (KBr), νmax (cm^−1^): 1278 (N-CH_3_), 1626 (C=N), 3125, 3143 (phenolic OH); HRMS: *m*/*z* = 354.1273 (M_free neutral base_+1); ^1^H NMR (DMSO-*d*_6_, 500 MHz) δ: 2.37 (*s*, 3H, Bzd-CH_3_), 2.48 (*s*, 3H, Ar-CH_3_), 3.33 (s, 3H, -CH_3_), 6.28 (*d*, 1H, Bzd, *J* = 2.5 Hz), 6.34 (*dd*, 1H, Bzd, *J*_1_ = 2.5 Hz, *J*_2_ = 8.5 Hz), 6.38 (*s*, 1H, Th), 7.32 (*d*, 2H, Ar, *J* = 8 Hz), 7.41 (*d*, 3H, Bzd+Ar, *J* = 8.5 Hz); ^13^C NMR (DMSO-*d*_6_, 125 MHz) δ: 14.3 (-CH_3_), 21.3 (-CH_3_), 33.9 (-CH_3_), 98.2 (Th-C_5_), 103.2 (Bzd), 107.4 (Bzd), 112.5 (Bzd), 127.9 (Ar), 129.1 (Ar), 129.8 (Ar), 129.9 (Bzd), 139.4 (Ar), 141.6 (Th-C_4_), 160.1 (Bzd), 160.5 (Th-C_2_), 161.0 (Bzd-OH), 166.1 (Bzd-OH).

*4-((1E)-1-((4-(4-chlorophenyl)-3-methylthiazol-2(3H)-ylidene)hydrazineylidene)ethyl)benzene-1,3-diol hydrobromide* (**E3**): yellow solid; mp = 264 °C; yield = 56%; FT-IR (KBr), νmax (cm^−1^): 817 (C-Cl), 1278 (N-CH_3_), 1626 (C=N), 3073, 3143 (phenolic OH); HRMS: *m*/*z* = 374.0729 (M_free neutral base_+1); ^1^H NMR (DMSO-*d*_6_, 500 MHz) δ: 2.48 (*s*, 3H, Bzd-CH_3_), 3.33 (*s*, 3H, -CH_3_), 6.28 (*d*, 1H, Bzd, *J* = 2.5 Hz), 6.34 (*dd*, 1H, Bzd, *J*_1_ = 2 Hz, *J*_2_ = 9 Hz), 6.49 (*s*, 1H, Th), 7.41 (*d*, 1H, Bzd, *J* = 9 Hz), 7.55–7.60 (m, 4H, Ar); ^13^C NMR (DMSO-*d*_6_, 125 MHz) δ: 14.3 (-CH_3_), 33.9 (-CH_3_), 99.4 (Th-C_5_), 103.2 (Bzd), 107.4 (Bzd), 112.5 (Bzd), 129.3 (Ar), 129.7 (Ar), 130.0 (Bzd), 131.0 (Ar), 134.5 (Ar), 140.3 (Th-C_4_), 160.1 (Bzd), 160.8 (Th-C_2_), 161.0 (Bzd-OH), 166.0 (Bzd-OH).

*4-((1E)-1-((4-(4-fluorophenyl)-3-methylthiazol-2(3H)-ylidene)hydrazineylidene)ethyl)benzene-1,3-diol hydrobromide* (**E4**): yellow solid; mp = 268 °C; yield = 70%; FT-IR (KBr), νmax (cm^−1^): 1226 (C-F), 1275 (N-CH_3_), 1623 (C=N), 3125, 3143 (phenolic OH); HRMS: *m*/*z* = 358.1024 (M_free neutral base_+1); ^1^H NMR (DMSO-*d*_6_, 500 MHz) δ: 2.48 (*s*, 3H, Bzd-CH_3_), 3.32 (*s*, 3H, -CH_3_), 6.28 (*d*, 1H, Bzd, *J* = 2.5 Hz), 6.34 (*dd*, 1H, Bzd, *J*_1_ = 2.5 Hz, *J*_2_ = 8.5 Hz), 6.45 (*s*, 1H, Th), 7.36 (*t*, 2H, Ar, *J* = 8.5 Hz), 7.41 (*d*, 1H, Bzd, *J* = 8.5 Hz), 7.58–7.61 (*m*, 2H, Ar); ^13^C NMR (DMSO-*d*_6_, 125 MHz) δ: 14.3 (-CH_3_), 33.9 (-CH_3_), 98.9 (Th-C_5_), 103.2 (Bzd), 107.4 (Bzd), 112.5 (Bzd), 116.3 (Ar, C-F *ortho*, *J* = 21.87 Hz), 127.3 (Ar, C-F *para*, *J* = 3.5 Hz), 130.0 (Bzd), 131.7 (Ar, C-F *meta*, *J* = 8.75 Hz), 140.4 (Th-C_4_), 160.1 (Bzd), 160.7 (Th-C_2_), 161.0 (Bzd-OH), 161.1 (Ar, C-F *ipso*, *J* = 236.25 Hz), 166.0 (Bzd-OH).

*4-((1E)-1-((4-(4-methoxyphenyl)-3-methylthiazol-2(3H)-ylidene)hydrazineylidene)ethyl)benzene-1,3-diol hydrobromide* (**E5**): yellow solid; mp = 275 °C; yield = 66%; FT-IR (KBr), νmax (cm^−1^): 1070, 1104 (-OCH_3_), 1278 (N-CH_3_), 1624 (C=N), 3130, 3164 (phenolic OH); HRMS: *m*/*z* = 370.1225 (M_free neutral base_+1); ^1^H NMR (DMSO-*d*_6_, 500 MHz) δ: 2.47 (*s*, 3H, Bzd-CH_3_), 3.32 (*s*, 3H, -CH_3_), 3.82 (*s*, 3H, -OCH_3_), 6.28 (*d*, 1H, Bzd, *J* = 2.5 Hz), 6.36 (*m*, 2H, Th+Bzd), 7.06 (*d*, 2H, Ar, *J* = 9 Hz), 7.41 (*d*, 1H, Bzd, *J* = 8.5 Hz), 7.45 (*d*, 2H, Ar, *J* = 8.5 Hz); ^13^C NMR (DMSO-*d*_6_, 125 MHz) δ: 14.3 (-CH_3_), 33.9 (-CH_3_), 55.8 (-OCH_3_), 97.8 (Th-C_5_), 103.2 (Bzd), 107.4 (Bzd), 112.5 (Bzd), 114.7 (Ar), 123.0 (Ar), 129.9 (Ar), 130.7 (Bzd), 141.4 (Th-C_4_), 160.1 (Bzd), 160.4 (Th-C_2_), 160.5 (Ar), 161.0 (Bzd-OH), 166.1 (Bzd-OH).

*4-((1E)-1-((3-methyl-4-(4-nitrophenyl)thiazol-2(3H)-ylidene)hydrazineylidene)ethyl)benzene-1,3-diol hydrobromide* (**E6**): orange solid; mp = 270 °C; yield = 77%; FT-IR (KBr), νmax (cm^−1^): 1274 (N-CH_3_), 1345, 1514 (-NO_2_), 1609 (C=N), 3080, 3094 (phenolic OH); HRMS: *m*/*z* = 385.0971 (M_free neutral base_+1); ^1^H NMR (DMSO-*d*_6_, 500 MHz) δ: 2.49 (*s*, 3H, Bzd-CH_3_), 3.38 (*s*, 3H, -CH_3_), 6.28 (*d*, 1H, Bzd, *J* = 2.5 Hz), 6.35 (*dd*, 1H, Bzd, *J*_1_ = 2.5 Hz, *J*_2_ = 8.5 Hz), 6.71 (s, 1H, Th), 7.42 (*d*, 1H, Bzd, *J* = 9 Hz), 7.84 (*d*, 2H, Ar, *J* = 9 Hz), 8.33 (*d*, 2H, Ar, *J* = 8.5 Hz); ^13^C NMR (DMSO-*d*_6_, 125 MHz) δ: 14.4 (-CH_3_), 34.3 (-CH_3_), 101.8 (Th-C_5_), 103.2 (Bzd), 107.4 (Bzd), 112.4 (Bzd), 124.4 (Ar), 130.1 (Ar), 130.3 (Bzd), 137.0 (Ar), 139.7 (Th-C_4_), 148.0 (Ar), 160.3 (Bzd), 161.0 (Th-C_2_), 161.2 (Bzd-OH), 165.9 (Bzd-OH).

*4-((1E)-1-((3-methyl-4-(4-(trifluoromethyl)phenyl)thiazol-2(3H)-ylidene)hydrazineylidene)ethyl)benzene-1,3-diol hydrobromide* (**E7**): yellow solid; mp = 255 °C; yield = 63%; FT-IR (KBr), νmax (cm^−1^): 1162, 1327 (-CF_3_), 1277 (N-CH_3_), 1626 (C=N), 3067, 3146 (phenolic OH); HRMS: *m*/*z* = 408.0997 (M_free neutral base_+1); ^1^H NMR (DMSO-*d*_6_, 500 MHz) δ: 2.49 (*s*, 3H, Bzd-CH_3_), 3.36 (*s*, 3H, -CH_3_), 6.28 (*d*, 1H, Bzd, *J* = 2.5 Hz), 6.35 (*dd*, 1H, Bzd, *J*_1_ = 2.5 Hz, *J*_2_ = 9 Hz), 6.61 (*s*, 1H, Th), 7.42 (*d*, 1H, Bzd, *J* = 8.5 Hz), 7.81 (*d*, 2H, Ar, *J* = 8.5 Hz), 7.88 (*d*, 2H, Ar, *J* = 8.5 Hz); ^13^C NMR (DMSO-*d*_6_, 125 MHz) δ: 14.4 (-CH_3_), 34.1 (-CH_3_), 100.6 (Th-C_5_), 103.2 (Bzd), 107.4 (Bzd), 112.4 (Bzd), 123.4 (Ar), 126.2 (Ar), 130.04 (Ar), 130.09 (Bzd), 134.8 (Ar), 140.7 (Th-C_4_), 160.2 (Bzd), 161.05 (Th-C_2_), 161.06 (Bzd-OH), 166.0 (Bzd-OH).

*4-(2-(((E)-1-(2,4-dihydroxyphenyl)ethylidene)hydrazineylidene)-3-methyl-2,3-dihydrothiazol-4-yl)benzonitrile hydrobromide* (**E8**): yellow solid; mp = 262 °C; yield = 76%; FT-IR (KBr), νmax (cm^−1^): 1275 (N-CH_3_), 1606 (C=N), 2221 (-CN), 3072, 3100 (phenolic OH); HRMS: *m*/*z* = 365.1075 (M_free neutral base_+1); ^1^H NMR (DMSO-*d*_6_, 500 MHz) δ: 2.48 (*s*, 3H, Bzd-CH_3_), 3.36 (*s*, 3H, -CH_3_), 6.28 (*d*, 1H, Bzd, *J* = 2.5 Hz), 6.34 (*dd*, 1H, Bzd, *J*_1_ = 2.5 Hz, *J*_2_ = 9 Hz), 6.64 (*s*, 1H, Th), 7.42 (*d*, 1H, Bzd, *J* = 9 Hz), 7.60 (*d*, 2H, Ar, *J* = 8 Hz), 7.99 (*d*, 2H, Ar, *J* = 8 Hz); ^13^C NMR (DMSO-*d*_6_, 125 MHz) δ: 14.3 (-CH_3_), 34.2 (-CH_3_), 101.2 (Th-C_5_), 103.2 (Bzd), 107.4 (Bzd), 112.2 (Bzd), 112.4 (Ar), 118.9 (-CN), 129.9 (Ar), 130.0 (Bzd), 133.1 (Ar), 135.2 (Ar), 140.0 (Th-C_4_), 160.2 (Bzd), 161.0 (Th-C_2_), 161.1 (Bzd-OH), 165.9 (Bzd-OH).

### 4.3. In Vitro Assessment of Antioxidant Activity

Stock solutions (2 mM) which were used for all antioxidant assays were prepared by dissolving the reference antioxidants (Trolox and ascorbic acid) as well as compounds **E1**–**E8** in DMSO. These were further diluted with DMSO to obtain extra working solutions of 0.2 mM. The absorption spectra of all tested compounds were recorded in the 400–800 nm range, confirming the absence of absorption peaks near the experimental wavelengths. All assay measurements were performed against blank samples of DMSO. For activity evaluation, IC_50_ values were determined for compounds **E1**–**E8** in the antiradical assays (DPPH and ABTS) using Equation **(1)**. For the electron transfer assays (TAC, RP, FRAP, and CUPRAC), the activity of each compound was expressed as the number of mole equivalents relative to the reference standard, as calculated using Equation **(2)**. All assays were performed in triplicate.

(1) $$\text{radical scavenging }\;(\%)=\frac{\text{control absorbance}-\text{sample absorbance}}{\text{control absorbance}}\times 100$$

(2) $$\text{equivalents of control}=\frac{\text{sample absorbance}}{\text{control absorbance}}$$

#### 4.3.1. Antiradical Assays

For the DPPH assay, aliquots between 20–200 µL were taken from the 2 mM stock solutions of each compound, and the volumes were adjusted with DMSO to 200 µL. These were mixed with 1 mL of the DPPH reagent and incubated in the dark for 30 min, following established protocols [8,24,34].

The ABTS^•+^ decolorization assay, as reported by Re et al. and previously described by our group, was also employed. The stability of the ABTS^•+^ reagent was verified by monitoring absorbance at 734 nm over one hour, confirming a consistent value of approximately 0.7. The reagent was prepared in 0.1 M potassium phosphate buffer (pH 7.4) and activated overnight with MnO_2_ [30,35,36].

#### 4.3.2. Electron Transfer Assays

The total antioxidant capacity (TAC) assay was carried out according to the procedure described in our previous work, which was based on established literature methods. Briefly, 10 mL of the reagent solution (0.6 M H_2_SO_4_, 28 mM Na_3_PO_4_, and 4 mM (NH_4_)_6_Mo_7_O_24_) was combined with 1000 µL of each test compound or a standard solution (prepared from 2 mM stock solutions). The mixtures were incubated in sealed glass tubes at 95 °C for 90 min, then cooled to room temperature. Subsequently, 1000 µL of each sample was diluted with an equal volume of water, and absorbance was recorded at 695 nm [8,30,37].

For the reducing power (RP) assay, 1000 μL of each test compound or standard solution (prepared from 0.2 mM stock solutions) was mixed with 400 μL of phosphate buffer (0.2 M, pH 6.6) and 400 μL of 1% (*w*/*v*) K_3_[Fe(CN)_6_]. The mixtures were incubated in sealed tubes at 50 °C for 20 min, cooled to room temperature, and subsequently treated with 500 μL of 10% (*w*/*w*) trichloroacetic acid. After a further 30 min incubation at room temperature, 250 μL of each reaction mixture was combined with 140 μL of 0.1% (*w*/*v*) FeCl_3_ and 1000 μL of distilled water, and absorbance was recorded at 695 nm [8,30,36].

The ferric-reducing antioxidant potential (FRAP) assay relies on electron transfer from the tested antioxidant to Fe^3+^, reducing it to Fe^2+^. The generated Fe^2+^ ions subsequently form a blue-colored complex with 2,4,6-tripyridyl-s-triazine, which displays maximum absorbance at 593 nm. Here, 500 μL of each compound or reference solution (0.2 mM) was mixed with 600 μL of the FRAP reagent and 1200 μL of acetate buffer (0.3 M, pH 3.6), following previously described methods. The mixtures were thoroughly homogenized in the dark, and their absorbance was then recorded spectrophotometrically [36,38].

The CUPRAC (cupric-reducing antioxidant capacity) assay was employed to evaluate the electron-donating ability of the tested compounds. The procedure was adapted from the methods of Alam et al., Özyürek et al., and Apak et al. Briefly, 250 μL of 10 mM CuCl_2_, 1 mL of 1 M ammonium acetate buffer, and 250 μL of 7.5 mM neocuproine in ethanol were combined with 125 μL of each test or reference solution (0.2 mM). The mixtures were incubated with shaking for 30 min in the dark, and absorbance was subsequently measured at 450 nm [36,39,40].

### 4.4. Prediction of Antioxidant Activity by In Silico Methods

#### 4.4.1. Theoretical Quantum Calculation

Two principal mechanisms were considered to describe the potential antioxidant activities of compounds **E1**–**E8** as neutral free bases. The first is the electron transfer mechanism, assessed through frontier molecular orbital analysis, focusing on the energy levels of the highest occupied molecular orbital (HOMO) and the lowest unoccupied molecular orbital (LUMO). The second involves hydrogen atom transfer (HAT) through homolytic O–H bond cleavage, generating a phenoxy radical and a hydrogen atom (Ar–OH → Ar–O^•^ + H^•^). According to the literature, the HAT pathway is generally favoured by phenolic molecules, particularly in reactions with external radicals such as DPPH. To evaluate this process, the O–H bond dissociation energy (BDE) was determined using the relation: BDE = H(ArO^•^) + H(H^•^) − H(ArOH) [41,42,43,44,45].

Computational studies of **E1**–**E8** were performed with Spartan24 (Wavefunction, CA, USA). The neutral free bases structures and the corresponding phenoxy radicals generated by homolytic O–H bond cleavage were fully optimized at the B3LYP/6-311+G(d,p) level of theory using the default convergence criteria implemented in Spartan24. Vibrational frequency calculations were performed at the same level of theory to verify that the optimized structures corresponded to true minima, as confirmed by the absence of imaginary frequencies, and to obtain the thermochemical corrections required for BDE calculations. The BDE values were derived from the calculated enthalpies of the optimized parent molecules, the corresponding phenoxy radicals, and the hydrogen atom. Solvent effects were evaluated using the polarizable continuum solvation model in vacuum, in a polar solvent (ε = 37.22), in a nonpolar solvent (ε = 7.43), and in water (ε = 78.3). Geometry optimizations and frequency calculations were performed independently in each medium. For **E1**–**E8**, the HOMO and LUMO and derived descriptors, such as the HOMO–LUMO energy gap, were subsequently calculated [43,46,47].

#### 4.4.2. Prediction of Molecular Properties

To predict the pharmacokinetic profiles of the compounds as neutral free bases, key molecular properties were analyzed using SwissADME. This evaluation encompassed the calculation of the topological polar surface area (TPSA), the calculated octanol–water partition coefficient (MLogP), water solubility, the assessment of each compound’s compliance with Lipinski’s rule of five, gastrointestinal absorption and blood–brain barrier permeability [48,49,50,51].

### 4.5. Cytotoxicity Evaluation

**Cell culture/cell lines**

Human lung adenocarcinoma A549 cells and normal human fibroblast BJ cells were obtained from the American Type Culture Collection (ATCC, Manassas, VA, USA). A549 cells were cultured in high-glucose Dulbecco’s Modified Eagle Medium (DMEM), whereas BJ fibroblasts were maintained in low-glucose DMEM. Both culture media were supplemented with 10% fetal bovine serum (FBS). Cells were maintained in T75 culture flasks at 37 °C in a humidified atmosphere containing 5% CO_2_. Maintenance consisted in media change every 2 days and when the confluence of the flask reached approximately 85% the cells were detached using trypsin–EDTA solution and either subcultured or further used in the experiments [30,52,53].

**Cell viability**

A549 cells were seeded in 96-well plates at a density of 7 × 10^3^ cells/well, while BJ cells were seeded at 4 × 10^3^ cells/well. Following overnight attachment, cells were exposed to compounds **E1**–**E8** at concentrations ranging from 5 to 100 μM for 48 h. Cell viability was assessed using the resazurin reduction assay. After treatment, the culture medium was replaced with a 200 μM resazurin solution and incubated for 3 h. Fluorescence resulting from the conversion of resazurin to resorufin by metabolically active cells was measured using a Synergy 2 Multi-Mode microplate reader at excitation and emission wavelengths of 550 and 600 nm, respectively. The optical (intrinsic fluorescence) and biochemical (direct reduction of resazurin to resorufin/oxidation of resorufin to non-fluorescent compounds) interferences of the synthesized compounds were evaluated in cell-free conditions prior to performing the cytotoxicity assays. Experiments were performed in three independent biological replicates, each containing at least three technical replicates. Cell viability was expressed as a percentage relative to vehicle-treated control cells exposed to a culture medium containing 0.4% DMSO [53]. The IC_50_ values were calculated based on the dose–cellular viability curve ratio obtained by fitting the experimental data with a 4-parameter logistic curve (SigmaPlot 11).

## 5. Conclusions

A series of novel thiazolyl–polyphenolic derivatives (**E1**–**E8**) was synthesized and structurally confirmed by MS, NMR, and FT-IR analyses.

The antioxidant evaluation showed that the biological activity strongly depends on the electronic nature of the aromatic substituent. Compounds containing electron-donating groups, especially the methoxy derivative **E5**, showed the best antioxidant activity in most of the experimental assays. The DFT calculations were in good agreement with the experimental results, indicating that higher HOMO energies and lower O–H bond dissociation energies are associated with improved antioxidant activity.

SwissADME prediction showed that all synthesized compounds satisfy Lipinski’s rule of five and present suitable molecular properties for further development. Nevertheless, some limitations within the series were identified. In particular, the CF_3_-substituted derivative E7 exhibited the highest lipophilicity (MLogP = 5.66) and the lowest aqueous solubility (0.35 µg/mL), indicating that increased hydrophobicity may adversely affect its physicochemical profile despite its favorable antiproliferative activity. In contrast, the cyano derivative E8 showed a more favorable physicochemical profile, including moderate lipophilicity (MLogP = 2.74) and an aqueous solubility of 2.46 µg/mL, but displayed a comparatively weak combined antioxidant and cytotoxic profile. These observations indicate that compliance with Lipinski’s criteria alone does not ensure an optimal balance between solubility and biological activity.

The cytotoxicity study identified compound **E6** as the most active derivative against A549 lung adenocarcinoma cells, with higher activity than gefitinib and the highest selectivity toward cancer cells. Compound **E7** also showed good antiproliferative activity and favorable selectivity.

Overall, the experimental and computational results consistently demonstrate that changing the aromatic substituent modifies both the antioxidant and cytotoxic properties of these molecules. Electron-donating groups improve antioxidant activity, whereas strong electron-withdrawing groups favor antiproliferative activity. However, the results also highlight the need to balance biological potency with physicochemical properties, particularly lipophilicity and aqueous solubility. Further structural optimization should therefore focus on identifying substitution patterns that combine antioxidant and/or antiproliferative activity with an improved overall physicochemical profile. These findings provide a useful basis for the further optimization of this class of thiazolyl–polyphenolic derivatives.

Based on the observed structure–activity relationships, a possible strategy for further optimization would be the introduction of two substituents with complementary but moderate electronic effects on the aromatic ring, for example a weak electron-donating group together with a weak electron-withdrawing group. Such a design may help balance the electronic requirements associated with antioxidant and antiproliferative activities while avoiding the unfavorable physicochemical effects observed for strongly lipophilic or strongly electron-withdrawing substituents.

## Figures and Tables

**Table 1:** The half-maximal inhibitory concentrations (IC_50_) identified for compounds **E1**–**E8** using the DPPH^•^ and ABTS^•+^ assays ±SD (µM).

Compound	DPPH^•^	ABTS^•+^
**E1**	147.46 ± 7.35	14.19 ± 0.13
**E2**	110.18 ± 5.46	14.20 ± 0.23
**E3**	168.63 ± 7.47	14.62 ± 0.24
**E4**	152.68 ± 5.58	10.06 ± 0.39
**E5**	92.32 ± 5.94	10.53 ± 0.41
**E6**	*	10.94 ± 0.30
**E7**	218.22 ± 7.97	10.62 ± 0.26
**E8**	251.91 ± 6.83	12.73 ± 0.32
**Trolox**	33.45 ± 1.64	19.63 ± 0.81

* IC_50_ higher than the highest tested concentration.

**Table 2:** Electron transfer capacities of compounds **E1**–**E8** expressed as means of molar ascorbic acid and/or Trolox equivalents/mol of the tested compound ± SD.

Compound	TAC	RP	FRAP	CUPRAC
	Eq Ascorbic Acid	Eq Ascorbic Acid	Eq Trolox	Eq Trolox
**E1**	1.28 ± 0.07	0.85 ± 0.05	1.10 ± 0.02	2.12 ± 0.03
**E2**	1.42 ± 0.05	1.11 ± 0.07	1.03 ± 0.04	2.06 ± 0.10
**E3**	1.28 ± 0.05	0.81 ± 0.03	0.93 ± 0.03	2.12 ± 0.05
**E4**	1.36 ± 0.04	0.76 ± 0.01	1.08 ± 0.01	2.05 ± 0.05
**E5**	1.32 ± 0.06	1.30 ± 0.04	1.02 ± 0.01	2.13 ± 0.04
**E6**	1.41 ± 0.04	1.10 ± 0.10	0.82 ± 0.03	2.39 ± 0.08
**E7**	1.35 ± 0.09	0.89 ± 0.08	0.88 ± 0.01	2.20 ± 0.03
**E8**	1.36 ± 0.07	0.74 ± 0.06	0.92 ± 0.04	2.08 ± 0.01

**Table 3:** The energy level of the frontier molecular orbitals HOMO, LUMO and the HOMO-LUMO gap in vacuum, nonpolar, polar environments and water for compounds **E1**–**E8** (eV).

Compound	Vacuum			Nonpolar			Polar			Water		
	HOMO	LUMO	Gap	HOMO	LUMO	Gap	HOMO	LUMO	Gap	HOMO	LUMO	Gap
**E1**	−5.20	−1.39	3.81	−5.26	−1.45	3.81	−5.27	−1.47	3.80	−5.28	−1.47	3.81
**E2**	−5.15	−1.34	3.81	−5.23	−1.44	3.79	−5.25	−1.46	3.79	−5.25	−1.46	3.79
**E3**	−5.30	−1.60	3.70	−5.30	−1.47	3.83	−5.30	−1.48	3.82	−5.30	−1.48	3.82
**E4**	−5.28	−1.46	3.82	−5.28	−1.47	3.81	−5.29	−1.47	3.82	−5.29	−1.47	3.82
**E5**	−5.13	−1.34	3.79	−5.22	−1.44	3.78	−5.23	−1.46	3.77	−5.24	−1.47	3.77
**E6**	−5.54	−3.15	2.39	−5.42	−3.15	2.27	−5.40	−3.16	2.24	−5.40	−3.16	2.24
**E7**	−5.41	−1.98	3.43	−5.35	−1.73	3.62	−5.34	−1.69	3.65	−5.34	−1.68	3.66
**E8**	−5.49	−2.37	3.12	−5.38	−2.14	3.24	−5.37	−2.11	3.26	−5.37	−2.10	3.27

**Table 4:** The dipole moment of compounds **E1**–**E8** in vacuum, nonpolar, polar environments and water (D).

Compound	Vacuum	Nonpolar	Polar	Water
**E1**	4.25	5.53	5.73	5.77
**E2**	4.87	6.23	6.45	6.48
**E3**	2.60	3.57	3.78	3.80
**E4**	2.76	3.77	3.99	4.01
**E5**	4.65	5.73	5.90	5.93
**E6**	3.49	4.50	4.69	4.73
**E7**	2.15	3.03	3.20	3.22
**E8**	3.09	3.91	4.05	4.06

**Table 5:** The BDE for the phenolic O-H bonds from compounds **E1**–**E8** in different environments (kcal/mol).

Compound	Vacuum		Nonpolar		Polar		Water	
	O-H_1_	O-H_2_	O-H_1_	O-H_2_	O-H_1_	O-H_2_	O-H_1_	O-H_2_
**E1**	79.68	87.07	76.52	83.95	75.54	83.05	75.40	82.93
**E2**	79.50	86.74	76.42	83.72	75.44	83.01	75.28	82.88
**E3**	80.08	87.38	76.83	84.20	75.86	83.14	75.70	83.13
**E4**	79.98	87.33	76.70	84.16	75.72	83.15	75.55	83.02
**E5**	79.31	86.98	76.23	83.48	75.29	82.72	75.14	82.59
**E6**	81.10	87.98	77.82	84.93	76.77	84.24	76.62	84.13
**E7**	80.55	87.72	77.19	84.53	76.15	83.59	75.99	83.47
**E8**	80.82	87.88	77.48	84.74	76.36	83.81	76.21	83.70

**Table 6:** Physicochemical properties of compounds **E1**–**E8** (reported as neutral free bases) affecting pharmacokinetics.

Compound	MW *	Rotatable Bonds	HBA	HBD	TPSA (Å^2^)	MLogP	Water Solubility (µg/mL)	Lipinski Violations	GI Absorption	BBB Permeability
**E1**	339.41	3	4	2	98.35	2.81	1.14	0	High	No
**E2**	353.44	3	4	2	98.35	3.14	1.06	0	High	No
**E3**	373.86	3	4	2	98.35	1.48	0.56	0	High	No
**E4**	357.40	3	5	2	98.35	2.97	1.47	0	High	No
**E5**	369.44	4	5	2	107.58	3.08	1.88	0	High	No
**E6**	384.41	4	6	2	144.17	3.92	2.02	0	Low	No
**E7**	407.41	4	7	2	98.35	5.66	0.35	0	High	No
**E8**	364.42	3	5	2	122.14	2.74	2.46	0	High	No

MW = “molecular weight”; HBA = “hydrogen bond acceptors”; HBD = “hydrogen bond donors”; TPSA = “topological polar surface area”; GI = “gastrointestinal”; BBB = “blood–brain barrier”; * = reported as neutral free bases.

**Table 7:** IC_50_ values determined after 48 h exposure of A549 and BJ cells to compounds **E1**–**E8** and the corresponding selectivity indices (SI = IC_50_BJ/IC_50_A549).

Compound	IC_50_ (µM)		Selectivity Index
	A549	BJ	
**E1**	25.73	53.26	2.07
**E2**	26.30	46.76	1.77
**E3**	27.62	40.54	1.46
**E4**	20.78	43.11	2.07
**E5**	26.14	48.35	1.85
**E6**	5.97	15.78	2.64
**E7**	13.99	33.01	2.36
**E8**	27.36	41.66	1.52
**Gefitinib**	15.93	25.50	1.60

## Data Availability

Data is contained within the article or Supplementary Materials.

## Supplementary Materials

The following supporting information can be downloaded at https://www.mdpi.com/article/10.3390/molecules31203620/s1, Figures S1–S8: The IR spectra for compounds **E1**–**E8**; Figures S9–S16: The ^1^H NMR spectra for compounds **E1**–**E8**; Figures S17–S24: The ^13^C NMR spectra for compounds **E1**–**E8**; Figures S25–S32: The HRMS for compounds **E1**–**E8**; Figures S33–S40: The RP-HPLC chromatograms of compounds **E1**–**E8** recorded at 254 nm; Table S1: The depiction of HOMO and LUMO for the compounds **E1**–**E8**; Table S2: The electrostatic potential maps of compounds **E1**–**E8**.
