# Supplementary material for: Synthesis, Antioxidant Activity, In Silico Evaluation, and Cytotoxicity of Novel Polyphenolic–Methyliden–Hydrazono–Thiazole Derivatives
Source: Molecules. 2026 Oct 11;31(20):3620. doi: 10.3390/molecules31203620 (PMC13707546; doi:10.3390/molecules31203620)
Supplement: Supplementary file 1 [file molecules-31-03620-s001.zip › molecules-4515304-supplementary.pdf]

molecules

MDPI

Supplementary Materials

Synthesis, Antioxidant Activity, In Silico Evaluation, and Cytotoxicity of Novel Polyphenolic–Methyliden–Hydrazono–Thiazole Derivatives

Alexandra Cătălina Cornea [1], Gabriel Marc [2,*], Ionel Fizeșan [3], Ioana Ionuț [1], Cristina Moldovan [1], Anca Stana [1], Brîndușa Tiperciuc [1], Ionuț-Valentin Creștin [3], Adrian Pîrnău [4], Laurian Vlase [5], Ilioara Oniga [6] and Ovidiu Oniga [1]

1. Figures

1.1. The IR spectra of the compounds E1-E8

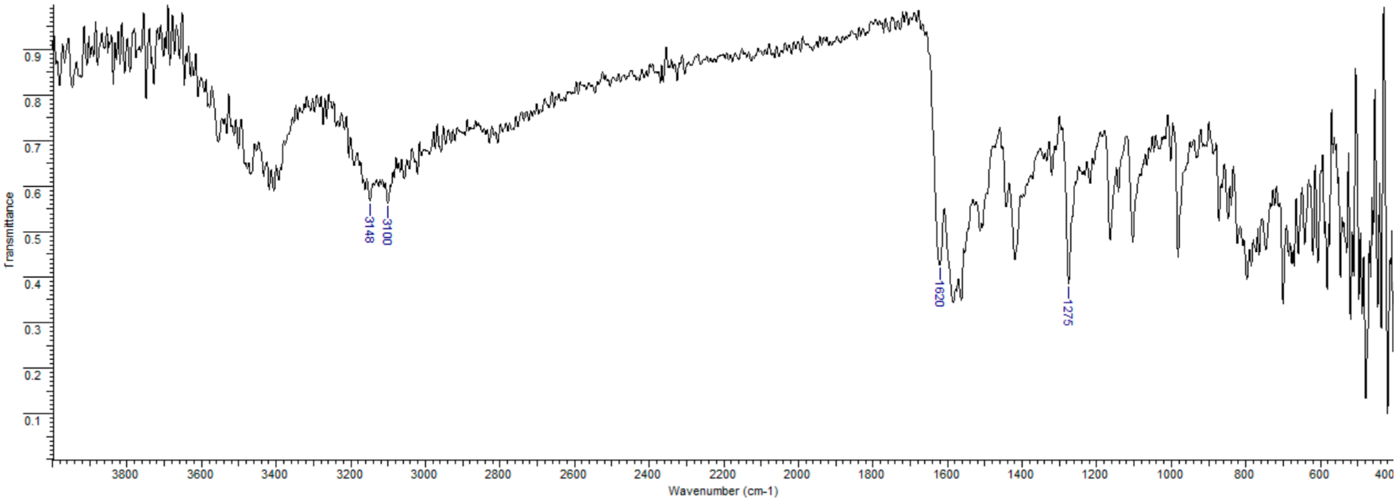

Figure S1. The IR spectrum of the compound **E1**

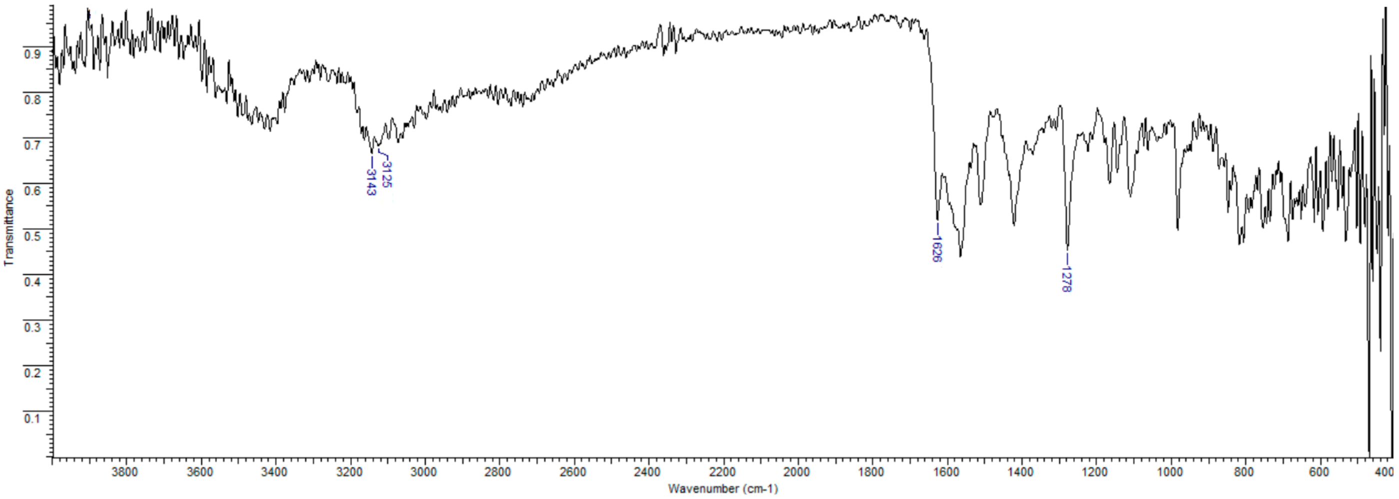

Figure S2. The IR spectrum of the compound **E2**

 https://doi.org/10.3390/molecules31203620

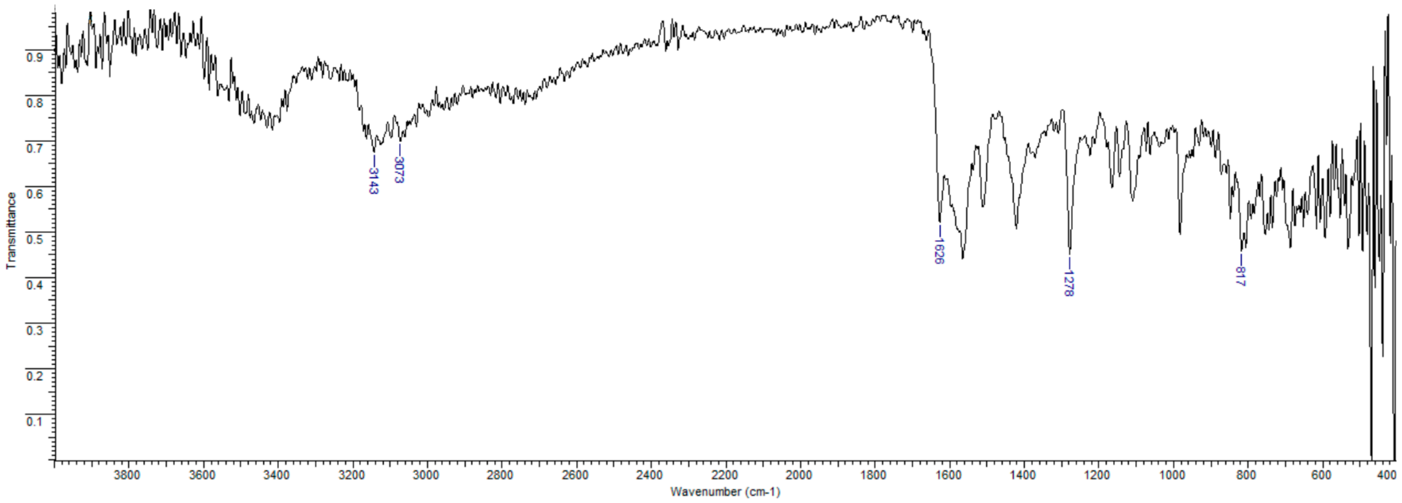

Figure S3. The IR spectrum of the compound **E3**

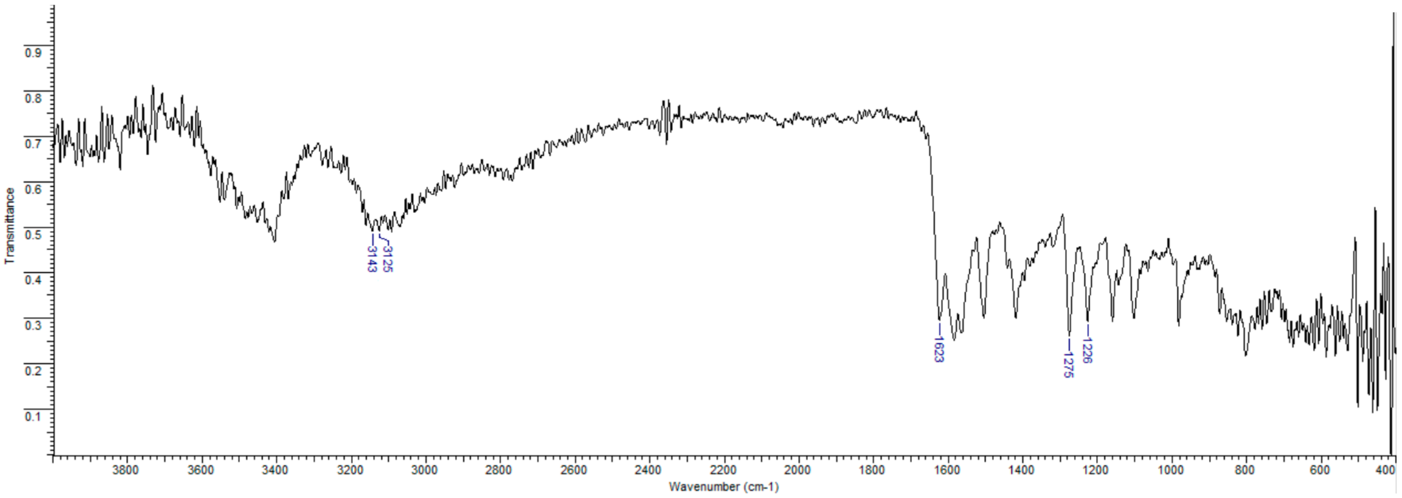

Figure S4. The IR spectrum of the compound **E4**

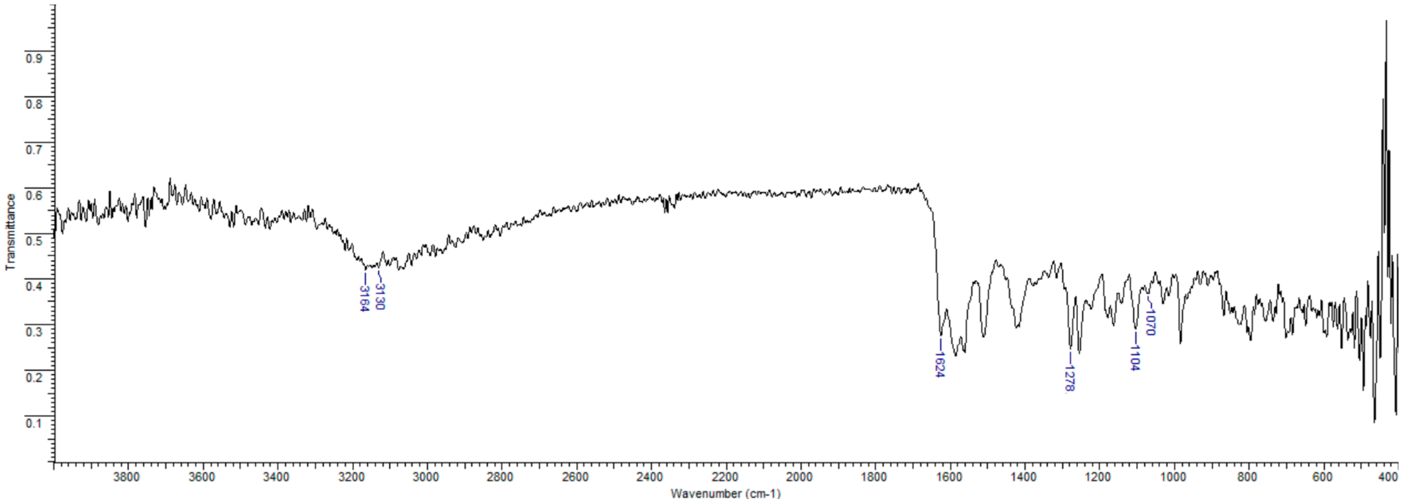

Figure S5. The IR spectrum of the compound **E5**

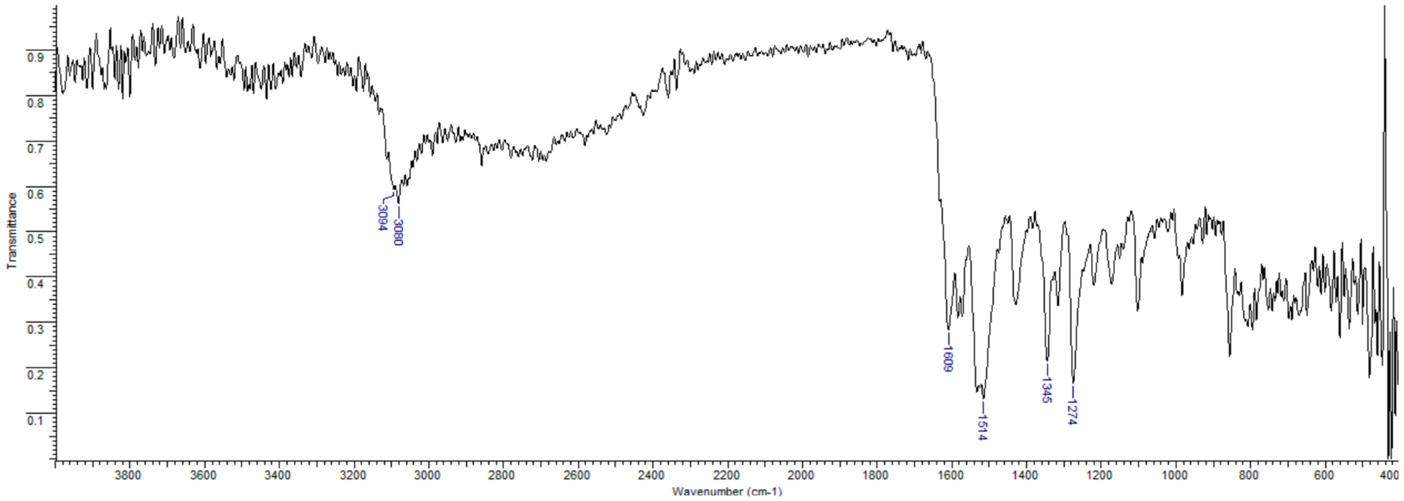

Figure S6. The IR spectrum of the compound **E6**

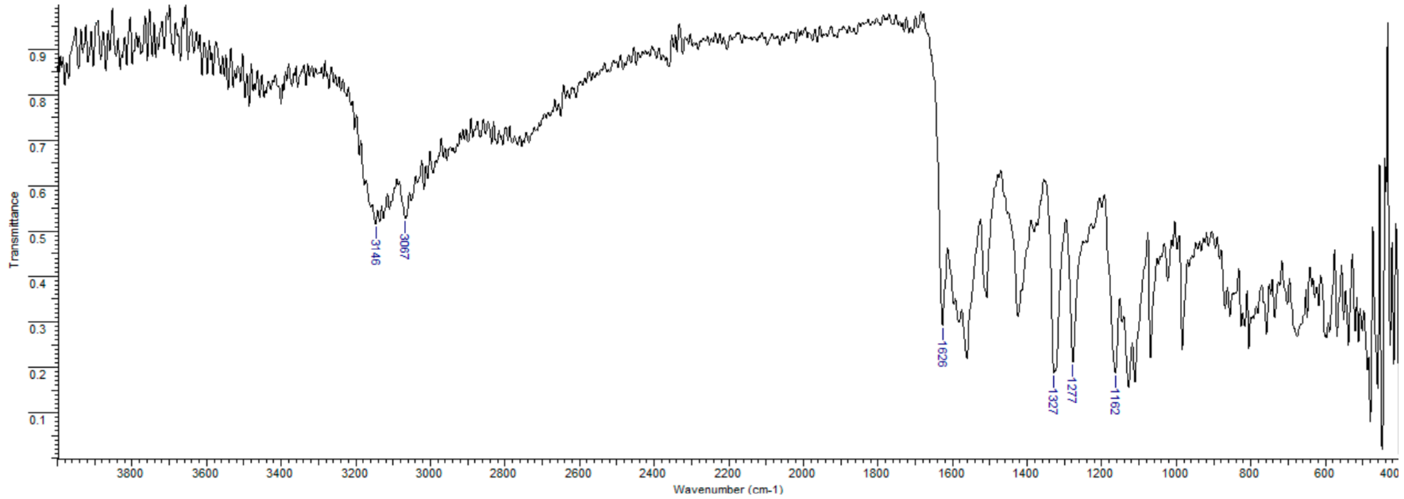

Figure S7. The IR spectrum of the compound **E7**

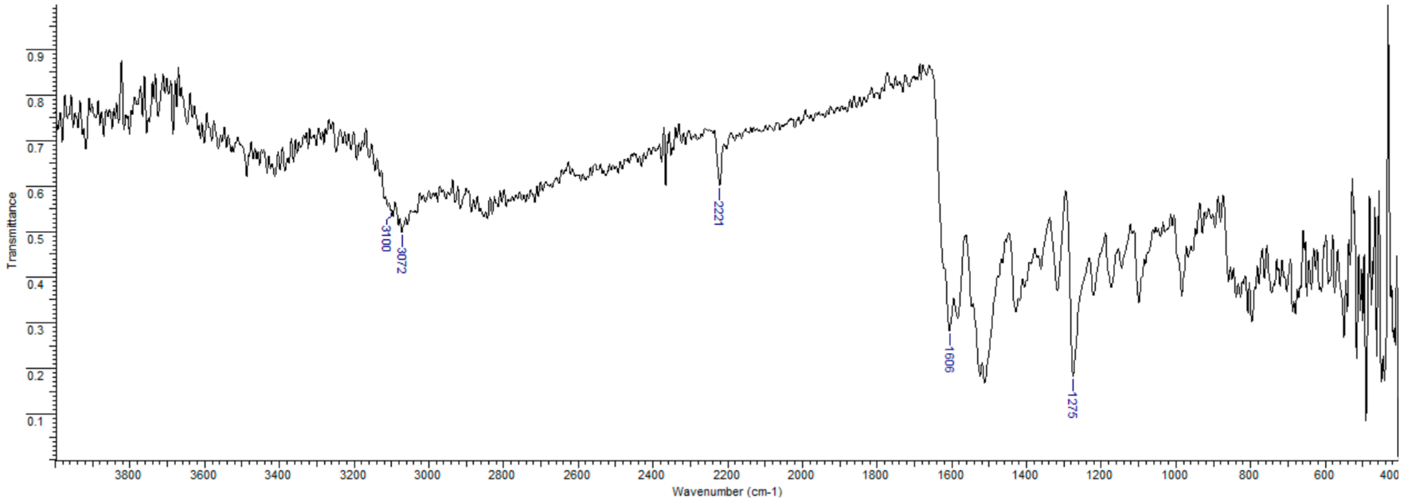

Figure S8. The IR spectrum of the compound **E8**

1.2 The [1]*H-NMR spectra of the compounds E1-E8*

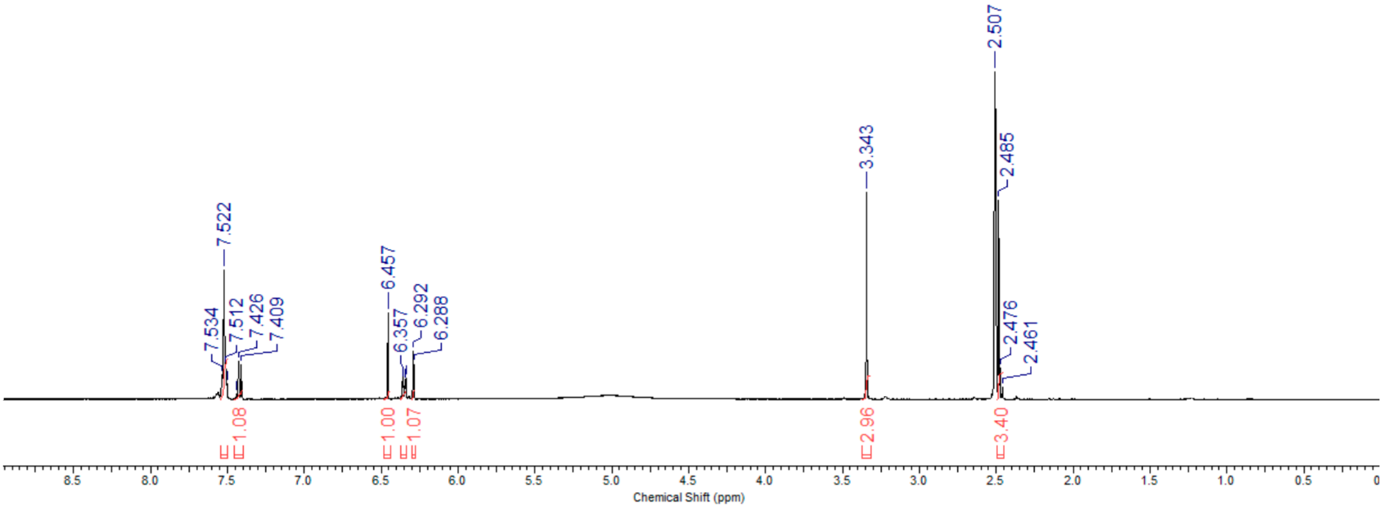

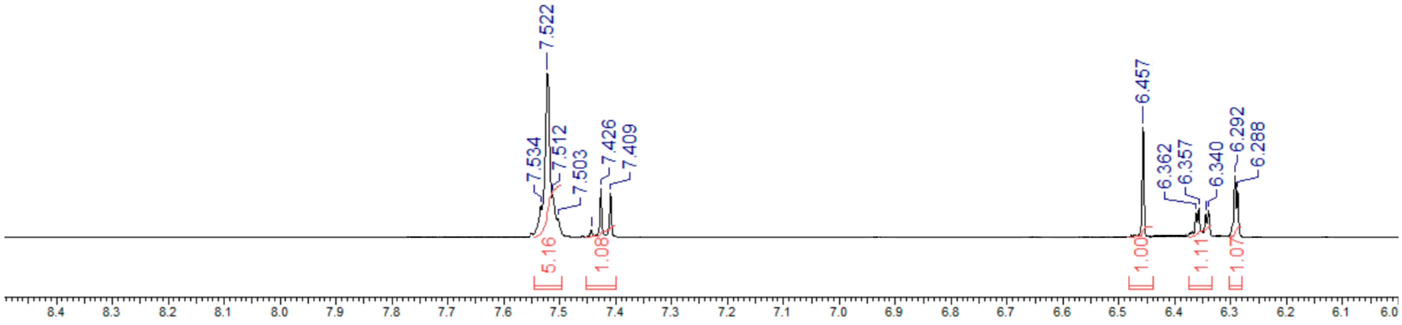

Figure S9. The ^{1}H-NMR spectrum of the compound **E1** (above) and zoom into aromatic area (below)

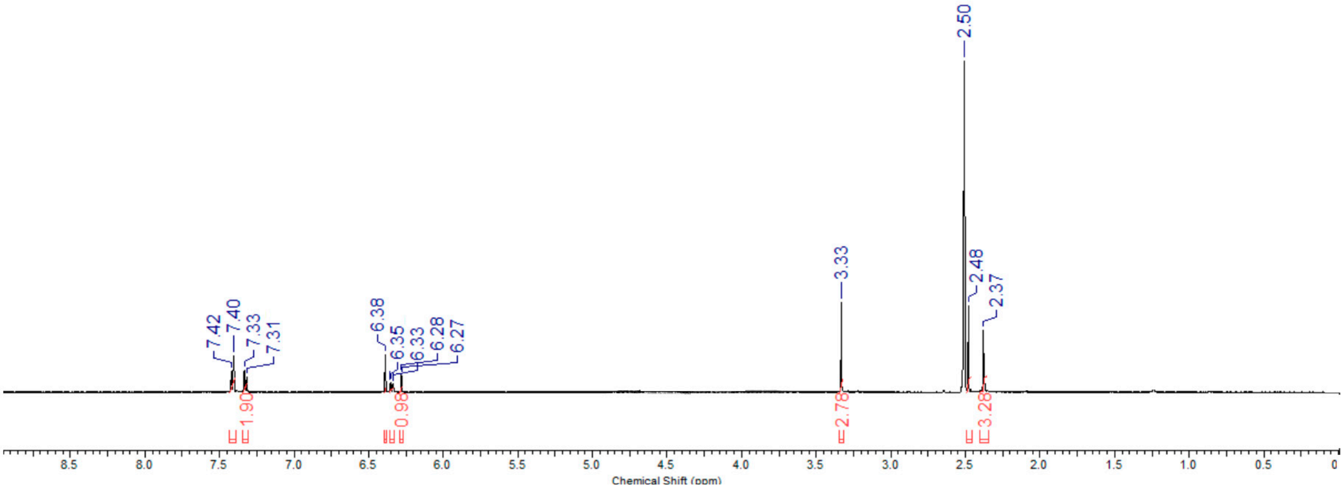

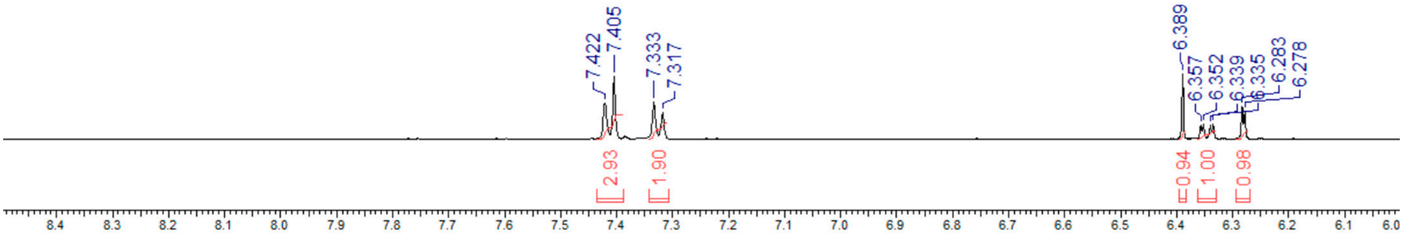

Figure S10. The ¹H-NMR spectrum of the compound **E2** (above) and zoom into aromatic area (below)

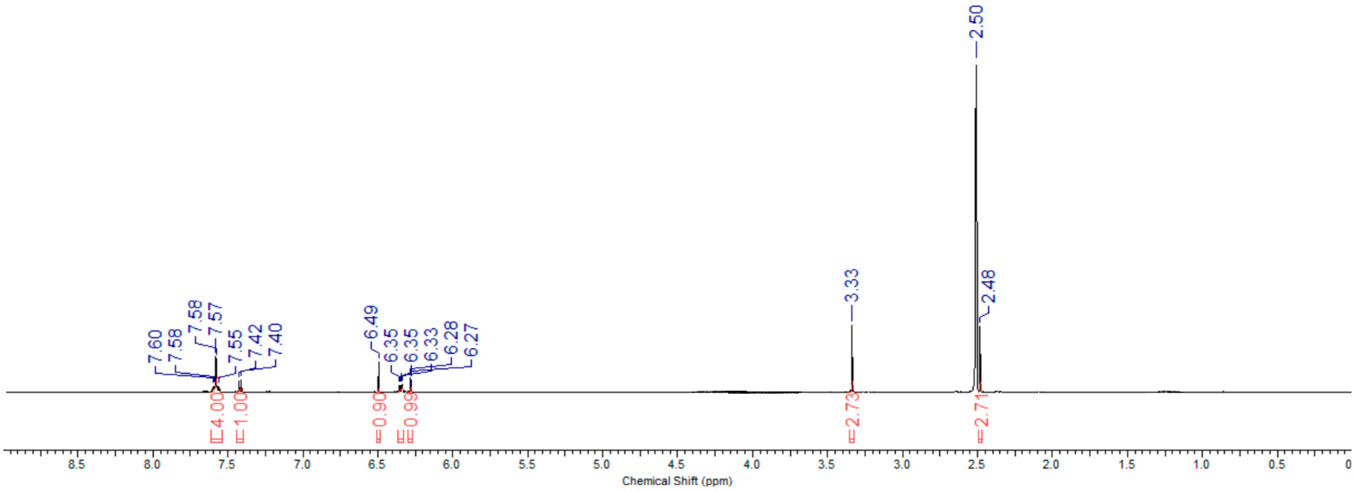

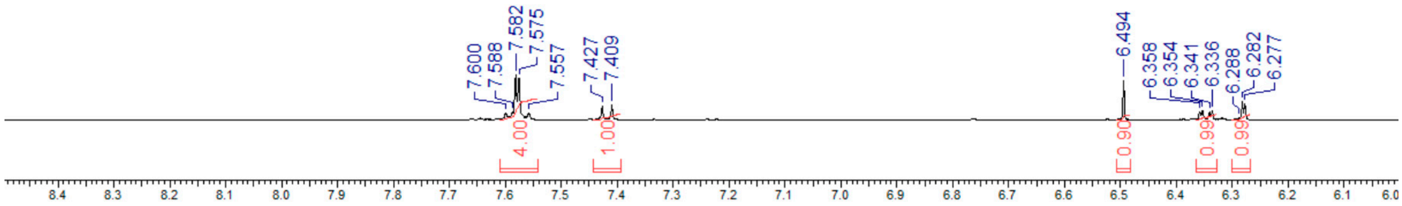

Figure S11. The ¹H-NMR spectrum of the compound **E3** (above) and zoom into aromatic area (below)

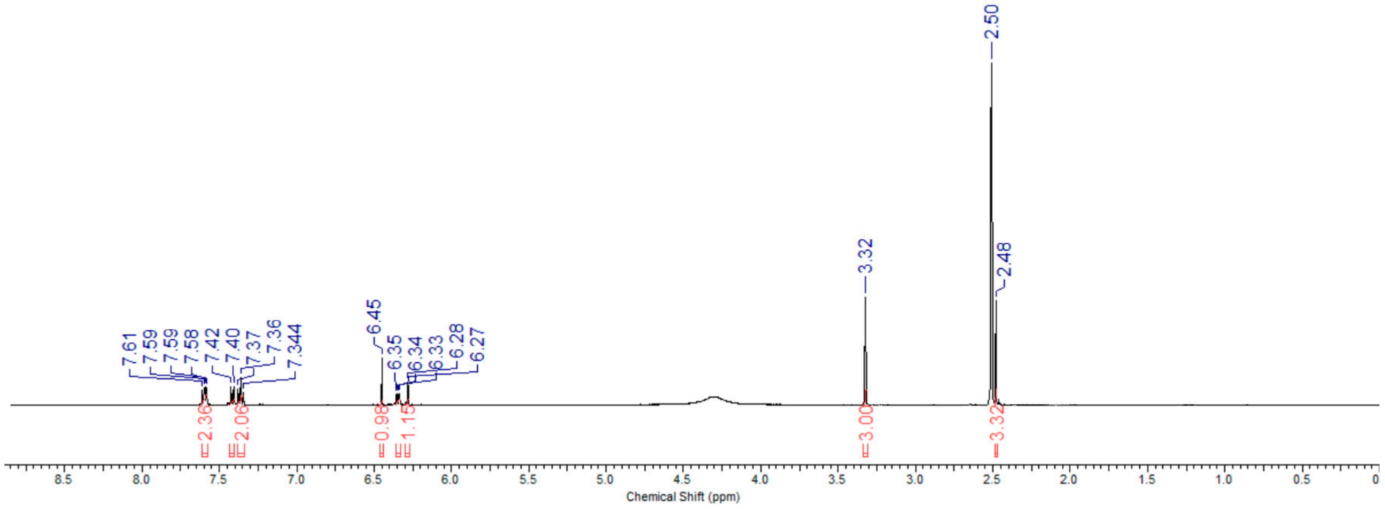

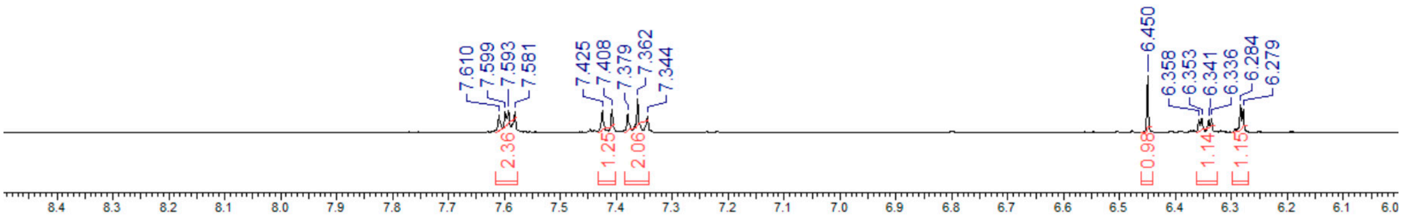

Figure S12. The ^{1}H-NMR spectrum of the compound **E4** (above) and zoom into aromatic area (below)

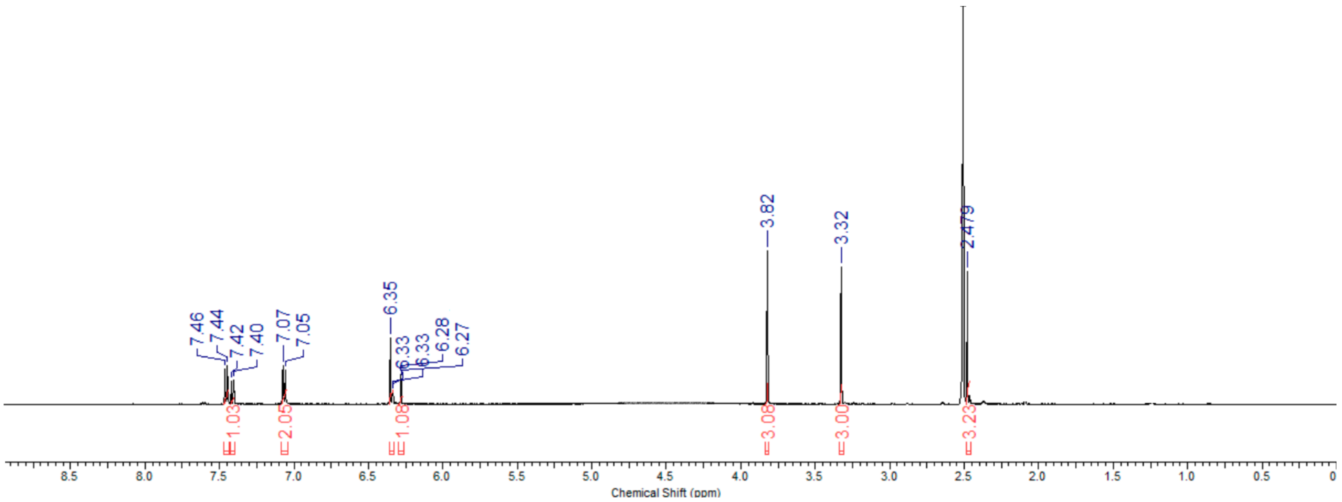

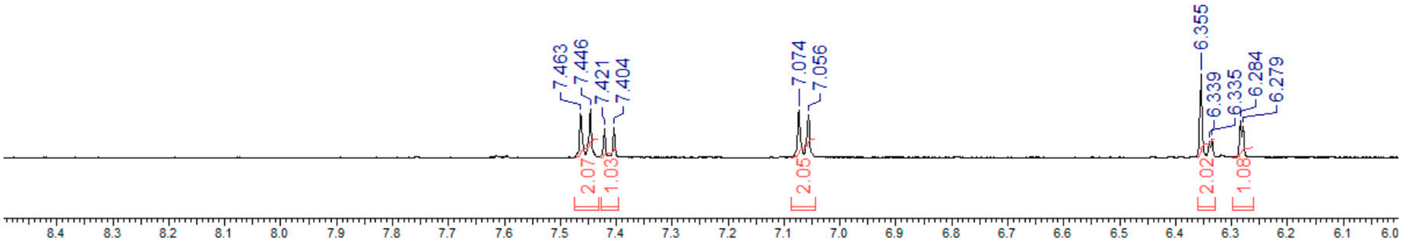

Figure S13. The ^{1}H-NMR spectrum of the compound **E5** (above) and zoom into aromatic area (below)

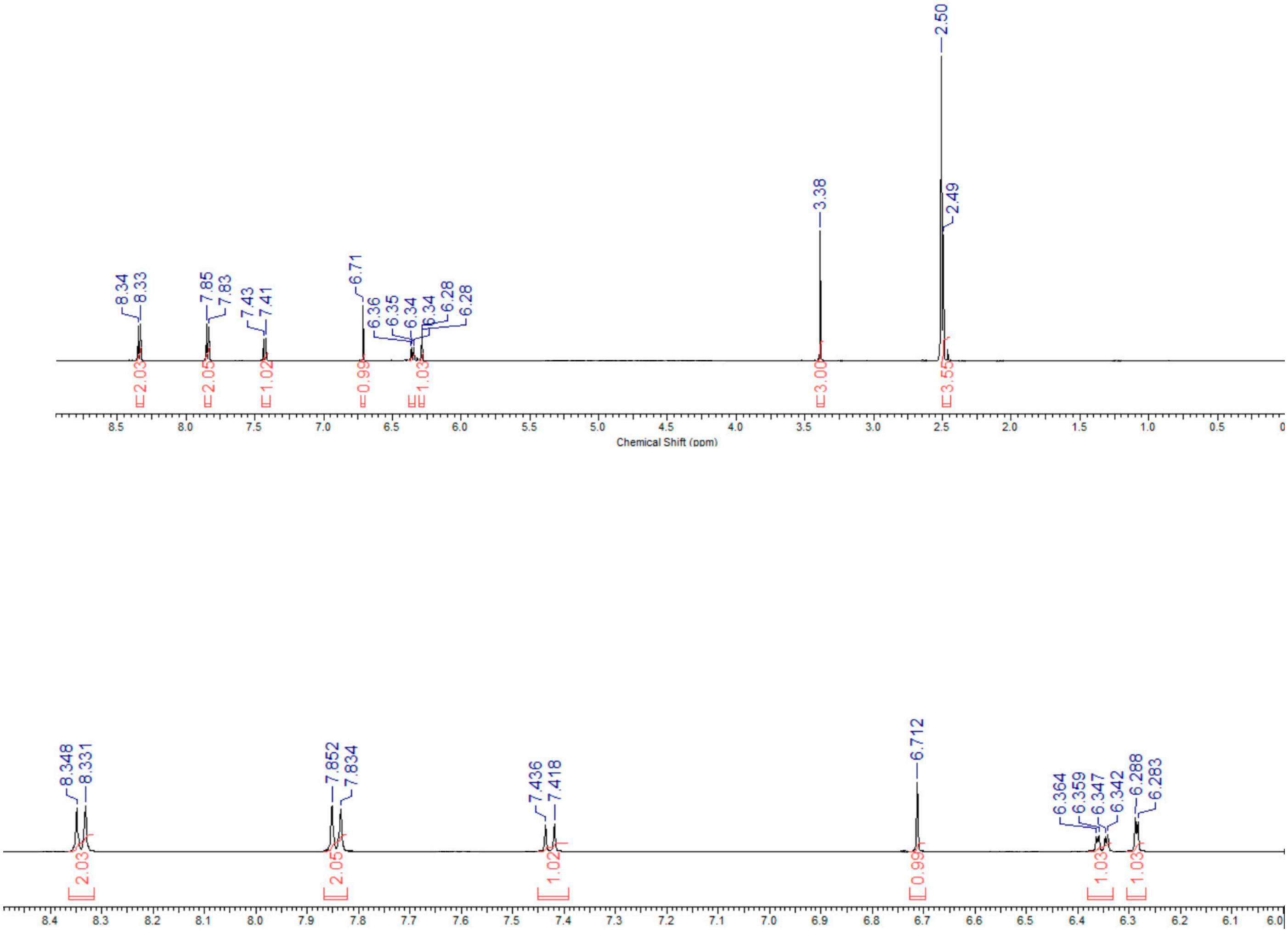

Figure S14. The ^{1}H-NMR spectrum of the compound **E6** (above) and zoom into aromatic area (below)

https://doi.org/10.3390/molecules31203620

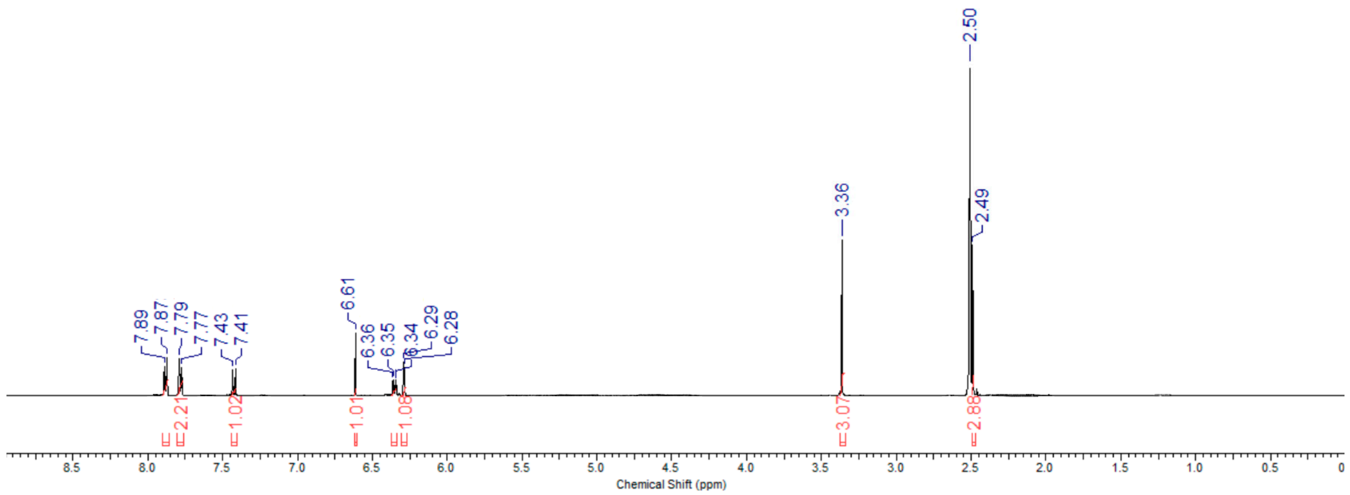

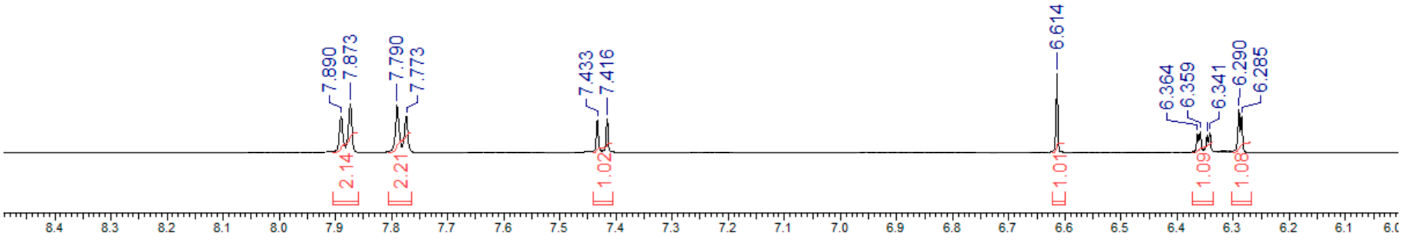

Figure S15. The ^{1}H-NMR spectrum of the compound **E7** (above) and zoom into aromatic area (below)

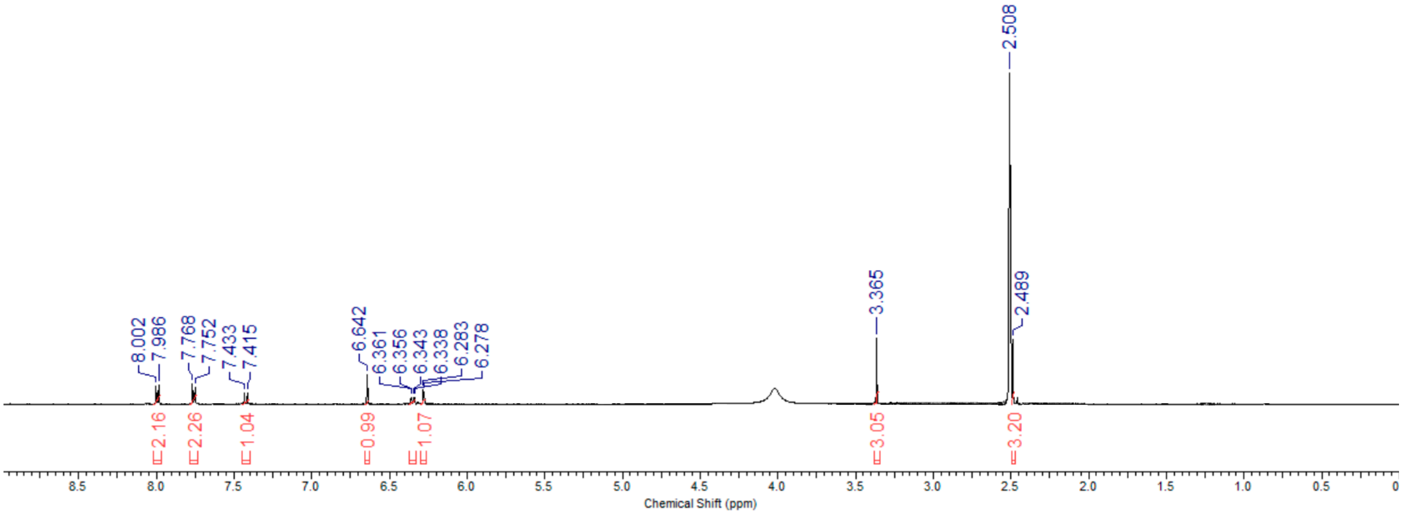

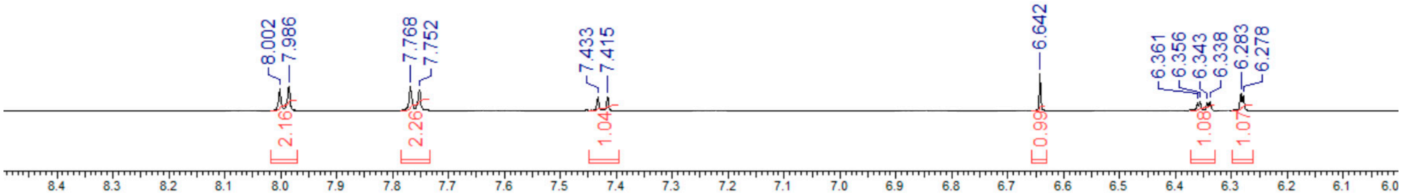

Figure S16. The ¹H-NMR spectrum if the compound **E8** (above) and zoom into aromatic area (below)

*1.3 The ¹³C-NMR spectra of the compounds **E1-E8***

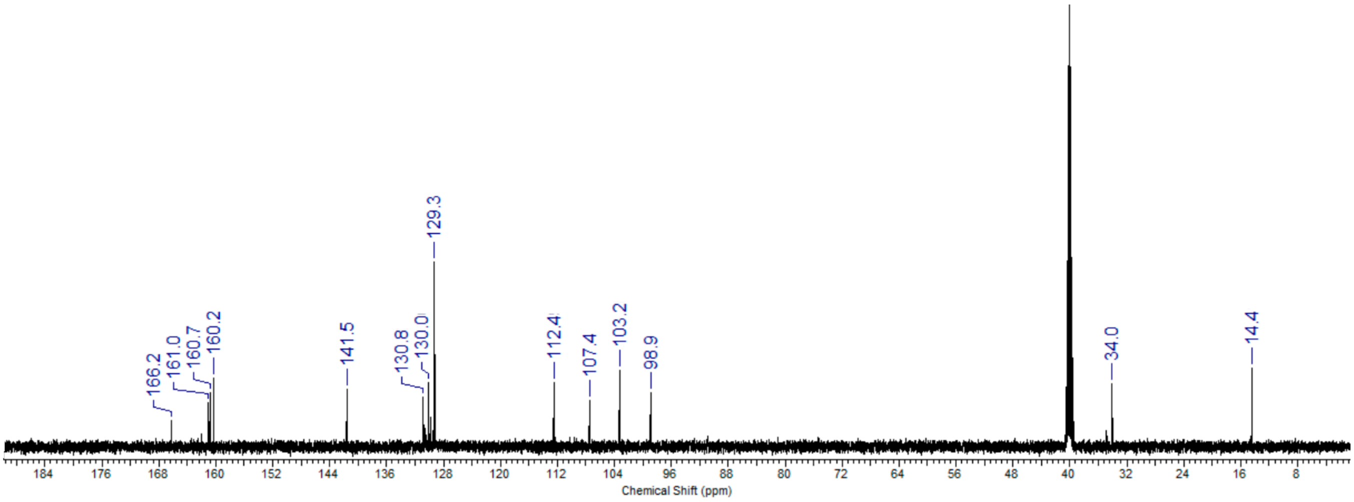

Figure S17. The ¹³C-NMR spectrum of the compound **E1**

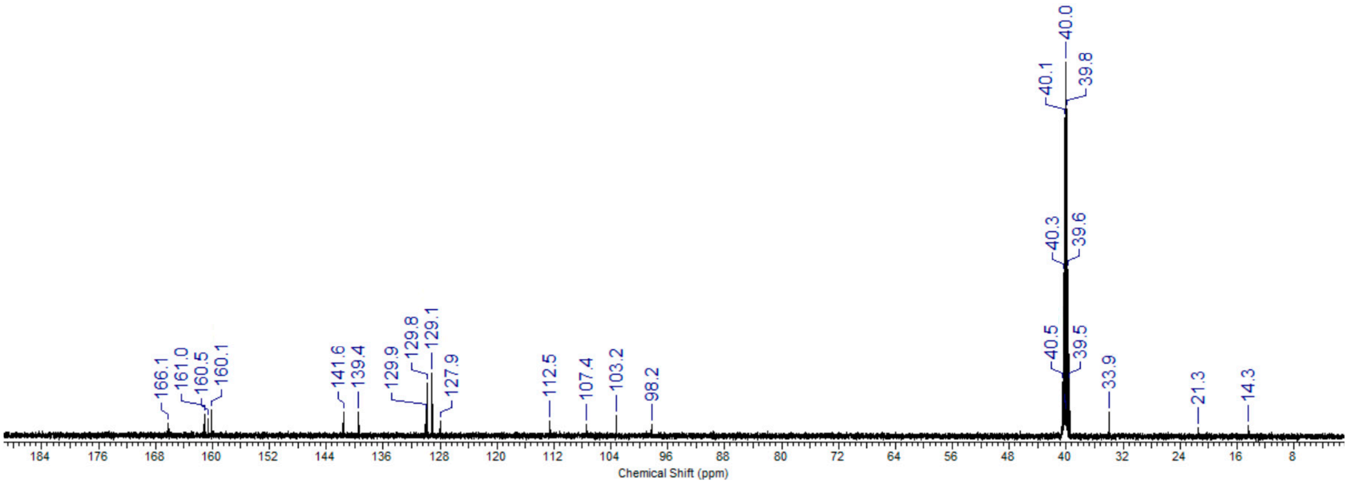

Figure S18. The ¹³C-NMR spectrum of the compound **E2**

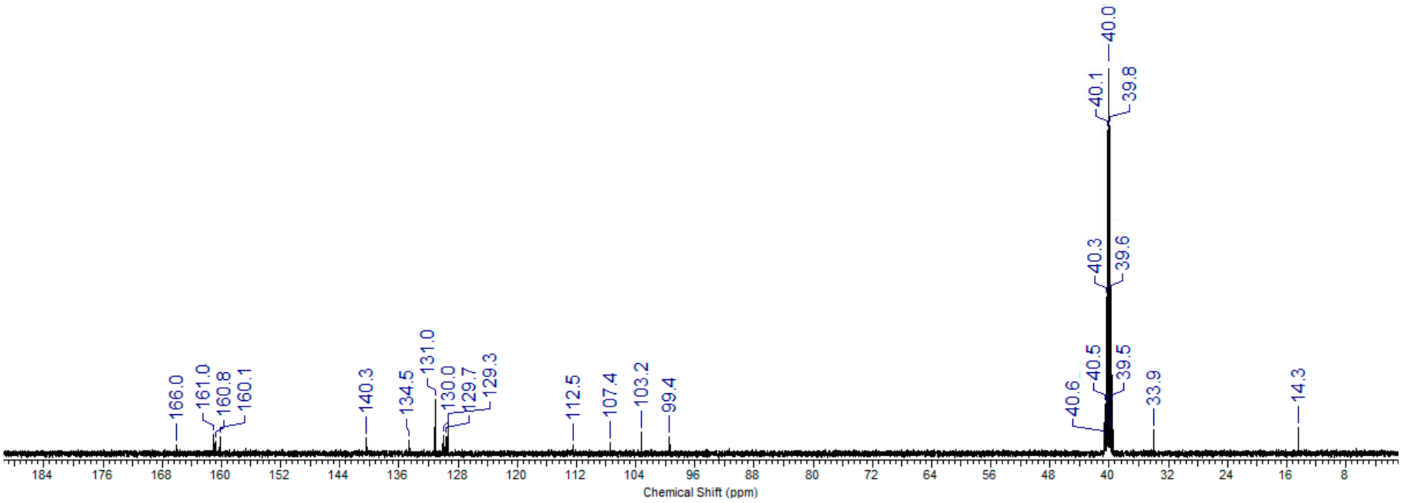

Figure S19. The ^{13}C-NMR spectrum of the compound **E3**

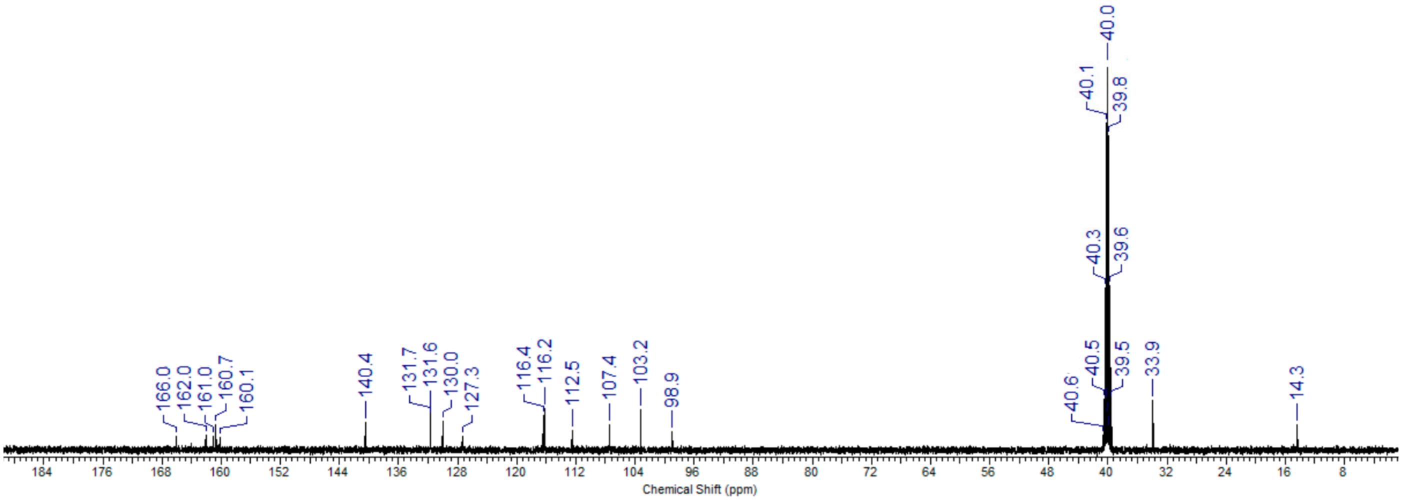

Figure S20. The ^{13}C-NMR spectrum of the compound **E4**

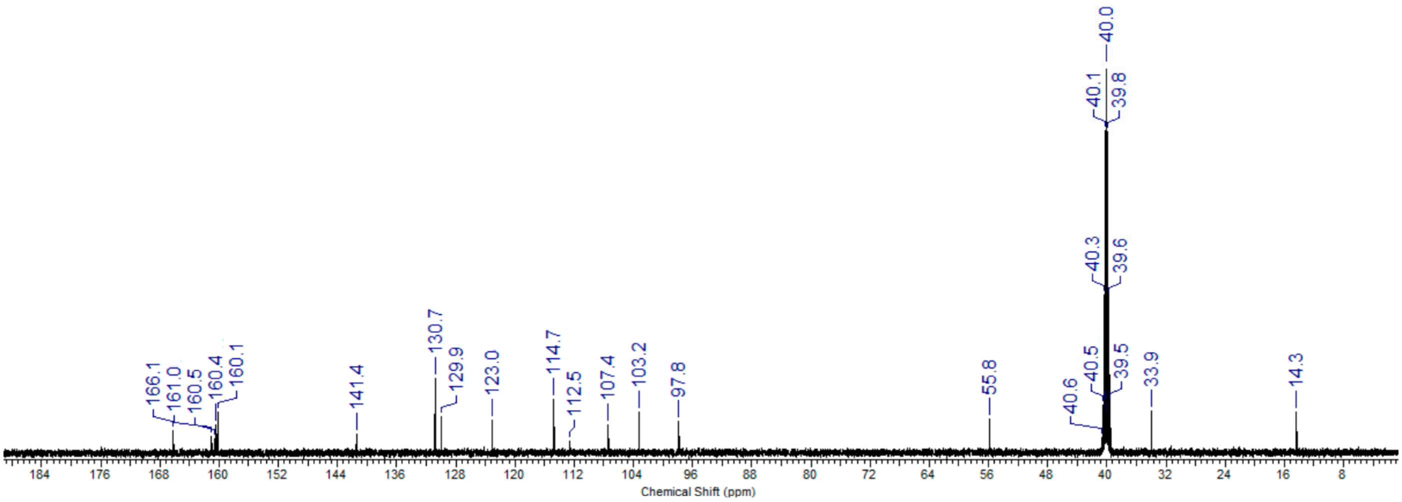

Figure S21. The ^{13}C-NMR spectrum of the compound **E5**

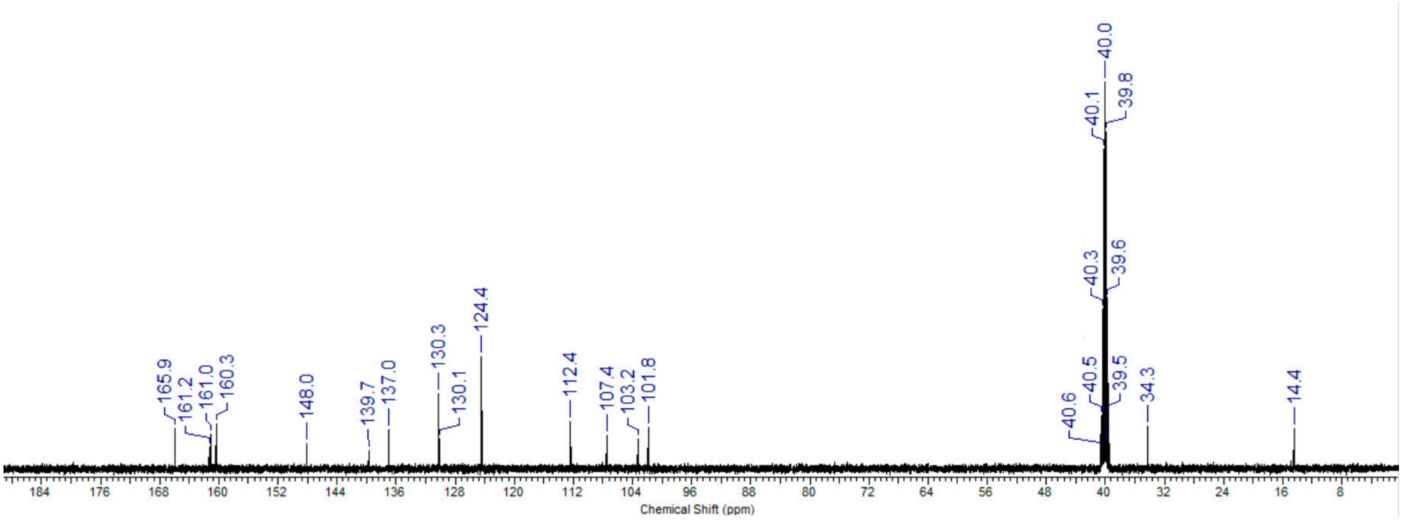

Figure S22. The ^{13}C-NMR spectrum of the compound **E6**

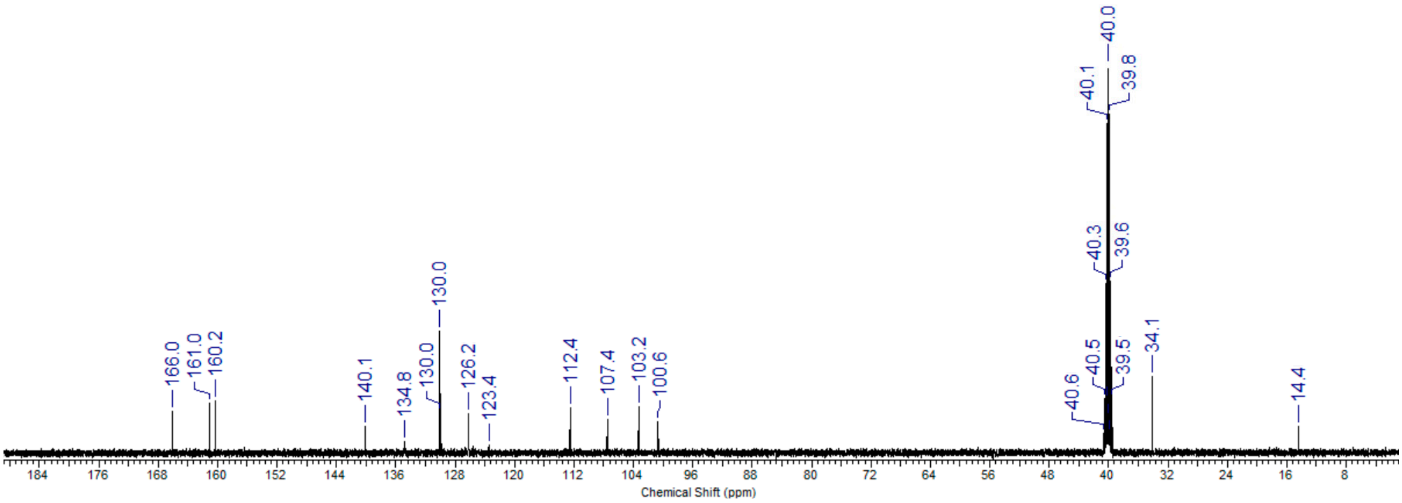

Figure S23. The ^{13}C-NMR spectrum of the compound **E7**

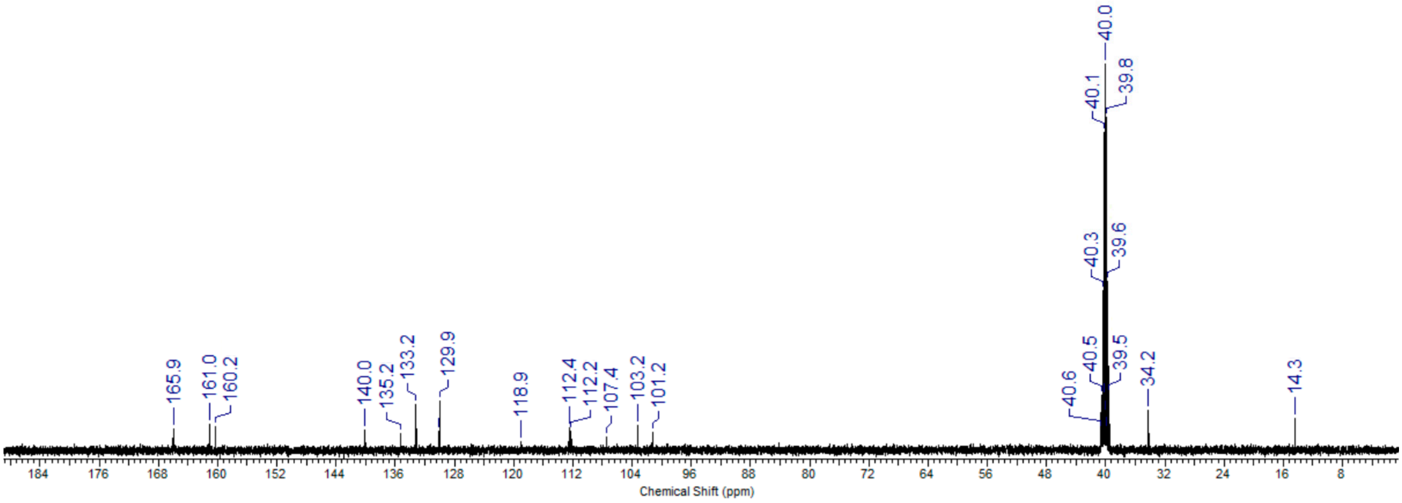

Figure S24. The ^{13}C-NMR spectrum of the compound **E8**

1.4 The HRMS of the compounds ***E1-E8***

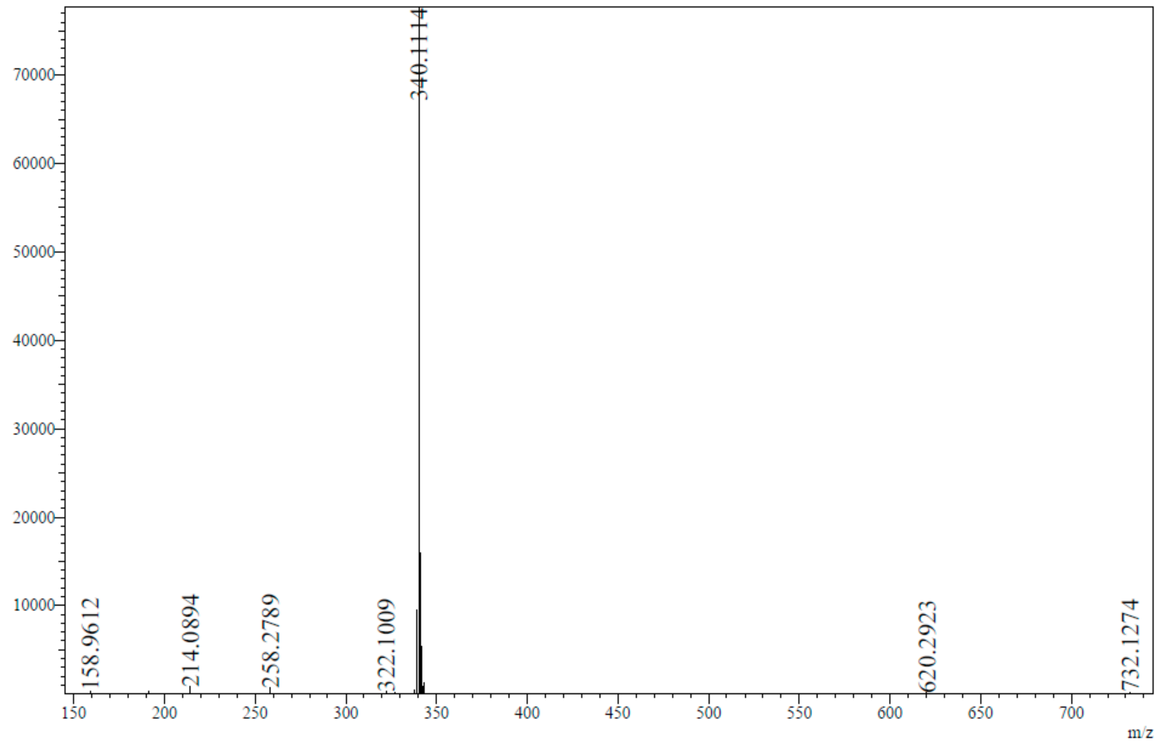

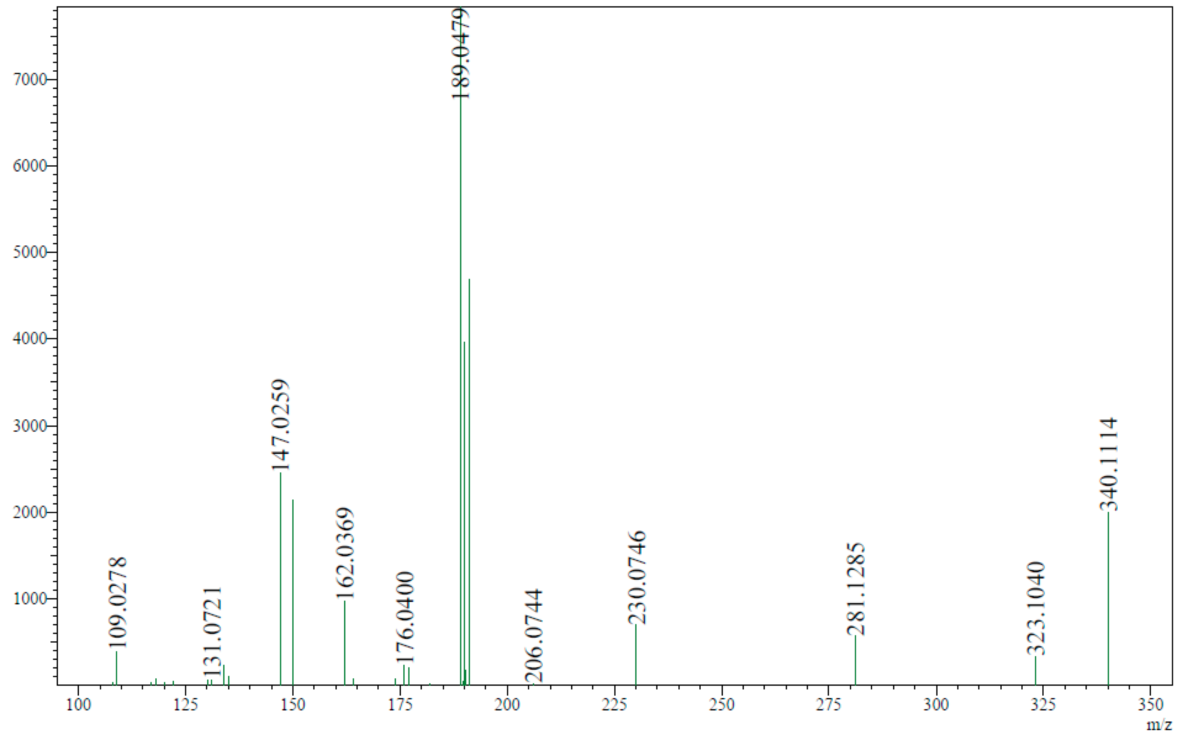

Figure S25. The HRMS spectrum of the compound **E1** (above) and MS/MS fragmentation spectrum of the corresponding molecular ion (below)

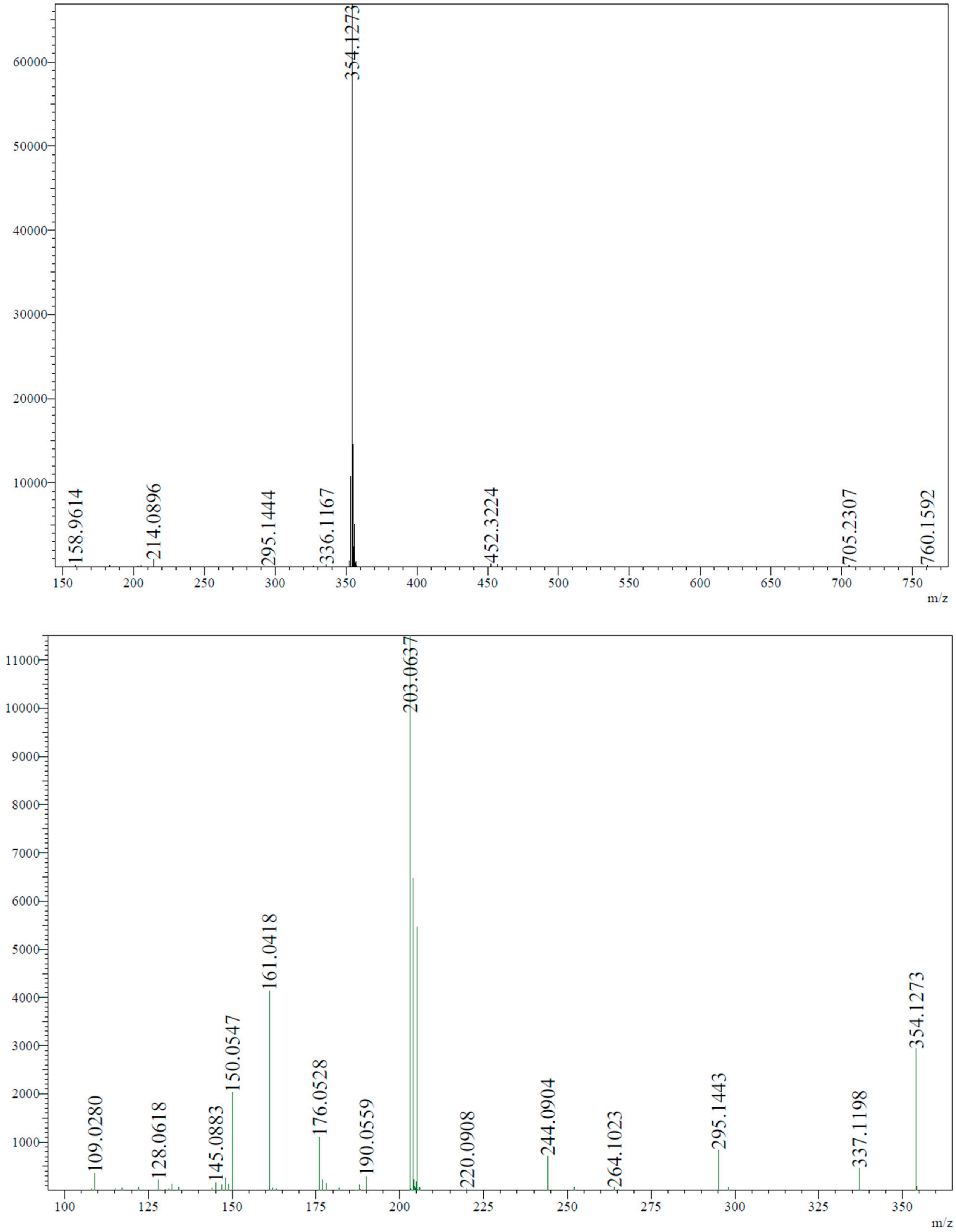

Figure S26. The HRMS spectrum of the compound **E2** (above) and MS/MS fragmentation spectrum of the corresponding molecular ion (below)

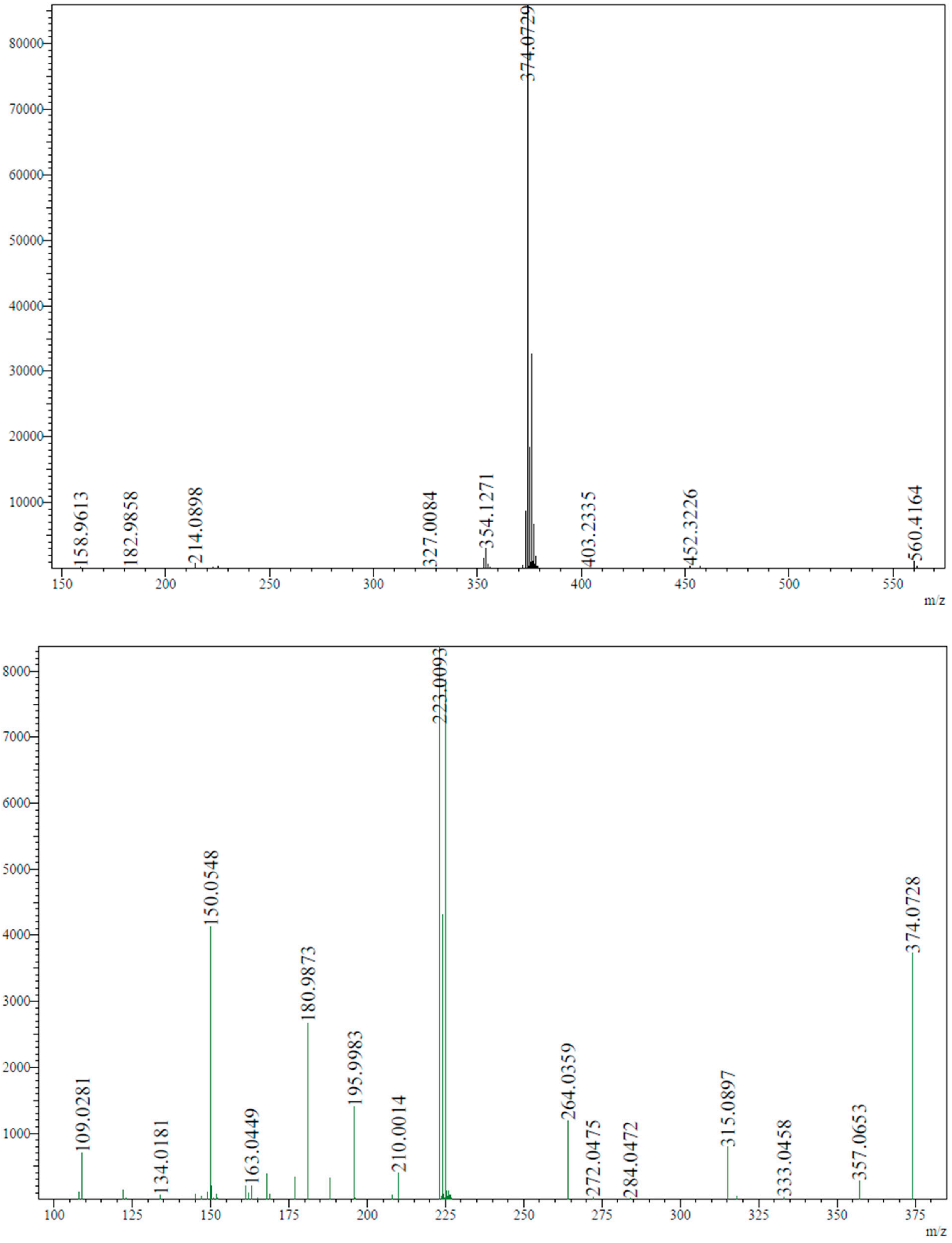

Figure S27. The HRMS spectrum of the compound **E3** (above) and MS/MS fragmentation spectrum of the corresponding molecular ion (below)

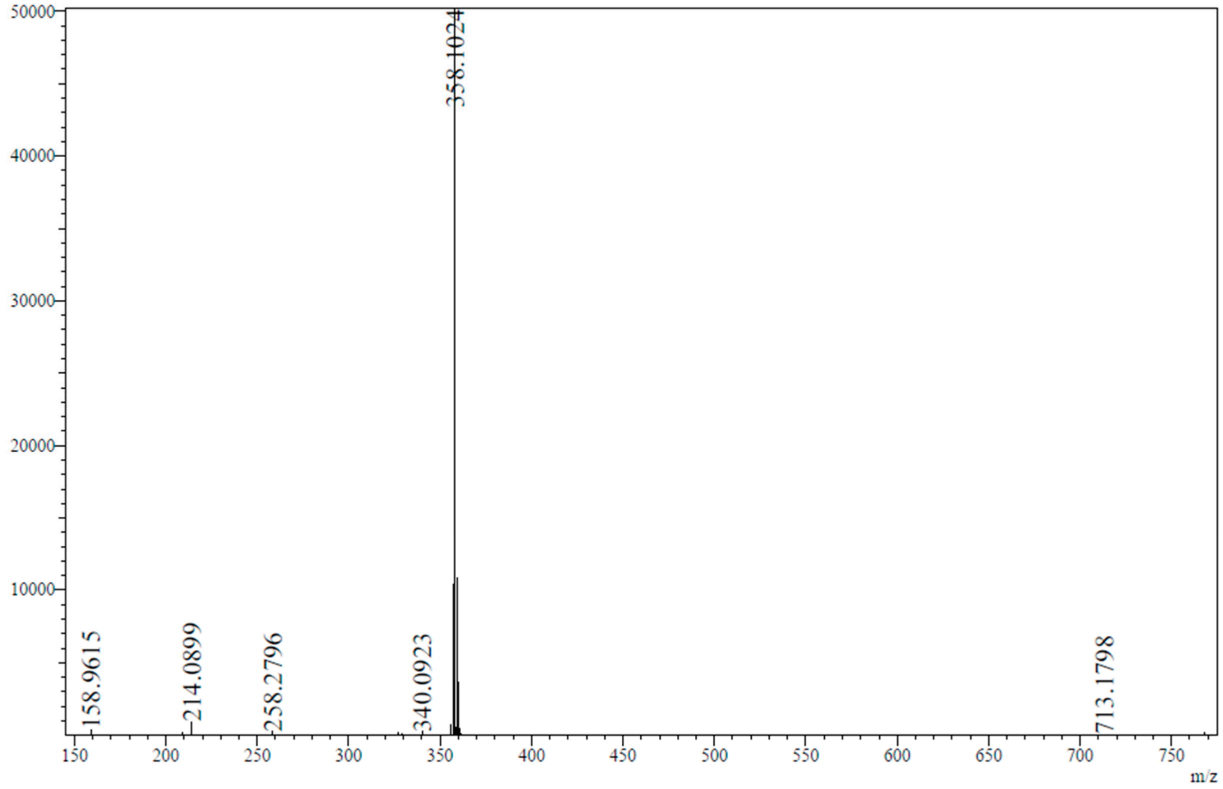

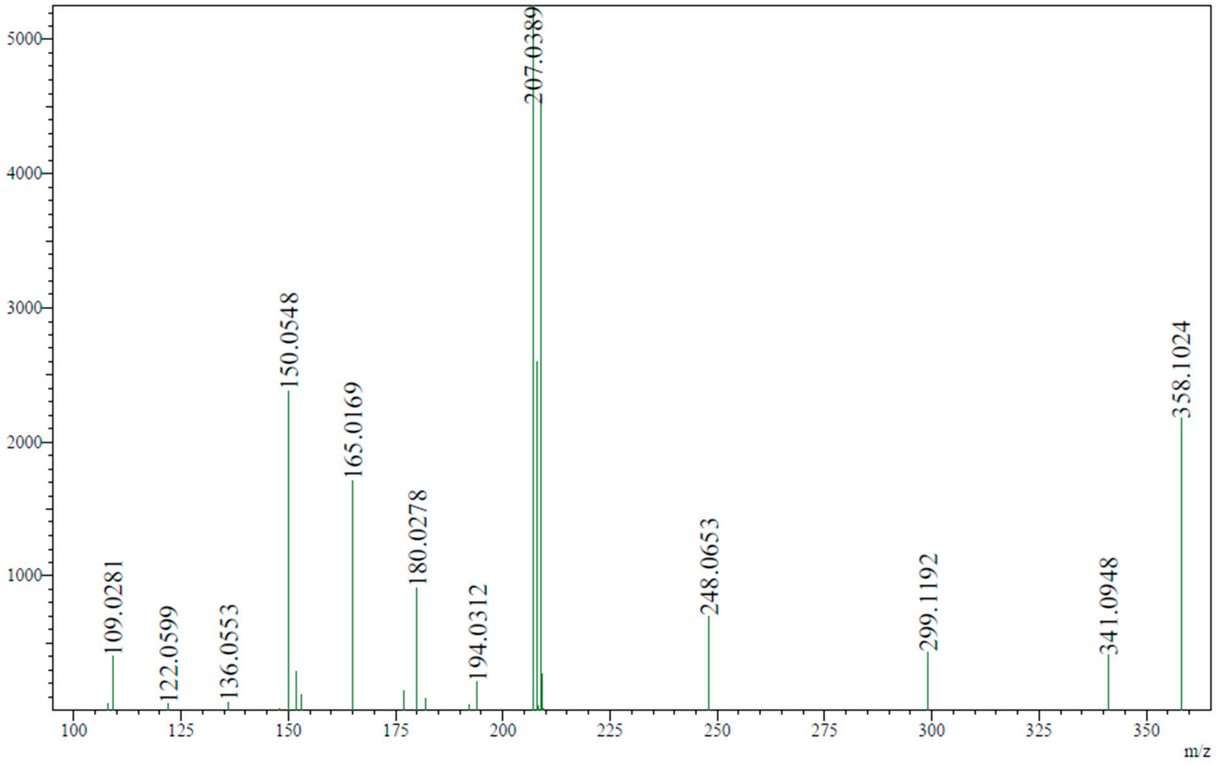

Figure S28. The HRMS spectrum of the compound **E4** (above) and MS/MS fragmentation spectrum of the corresponding molecular ion (below)

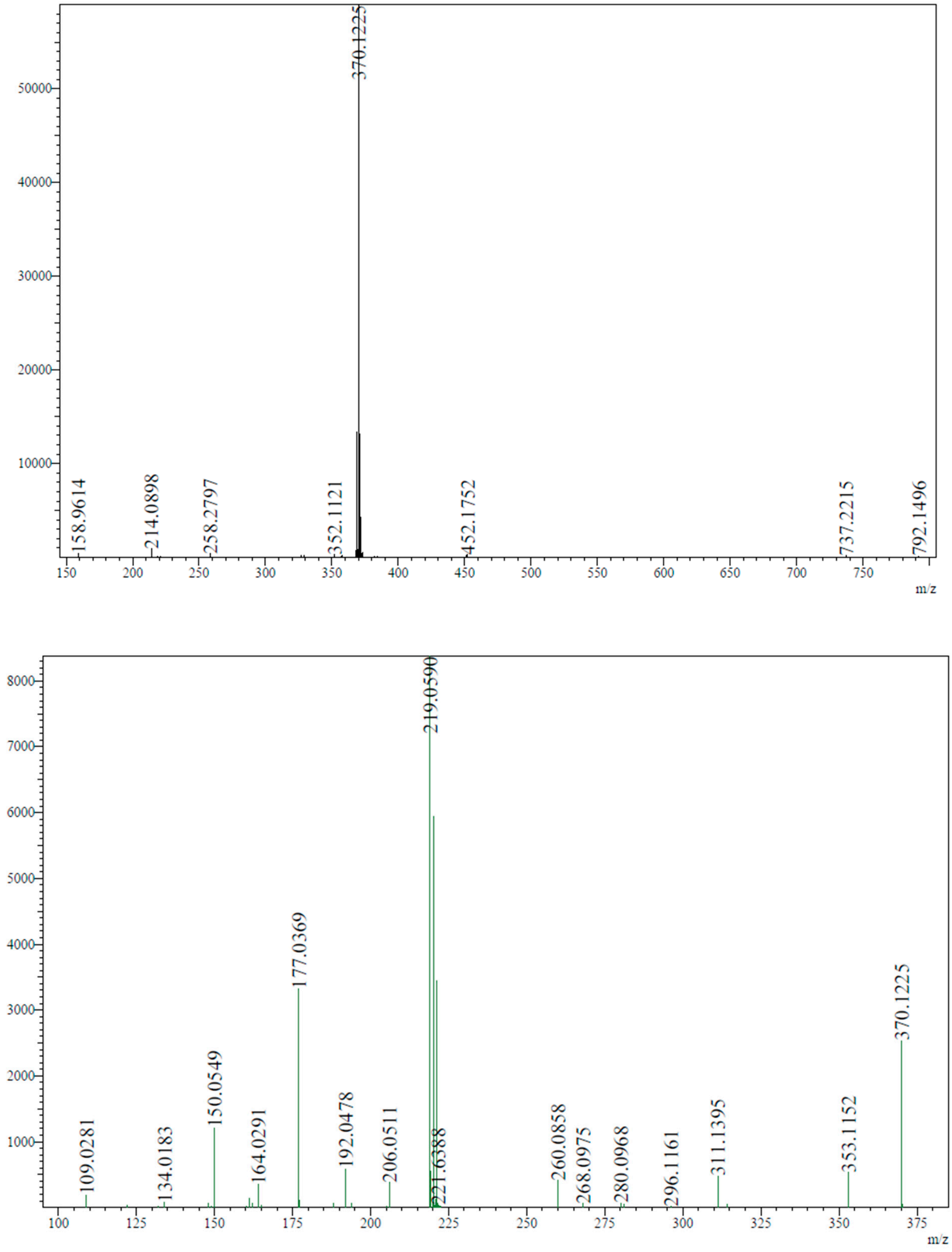

Figure S29. The HRMS spectrum of the compound **E5** (above) and MS/MS fragmentation spectrum of the corresponding molecular ion (below)

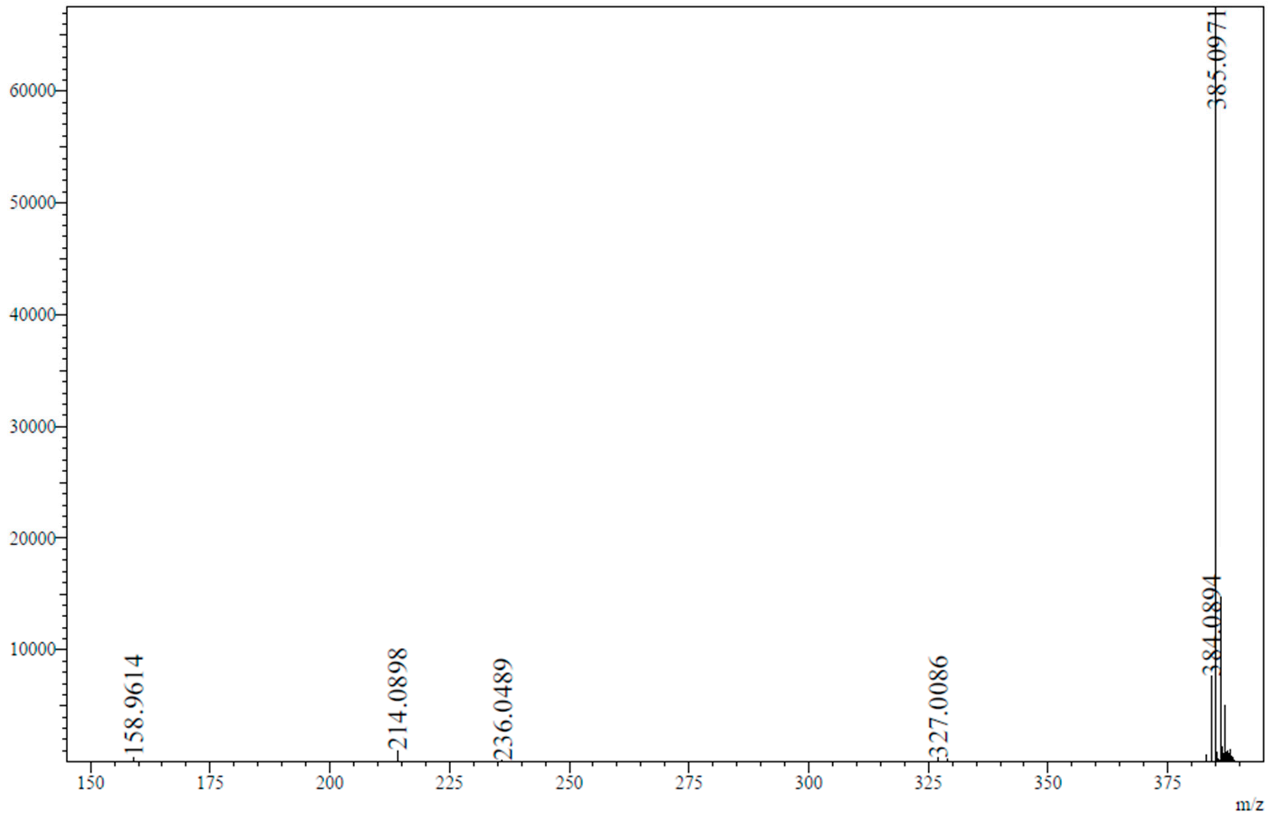

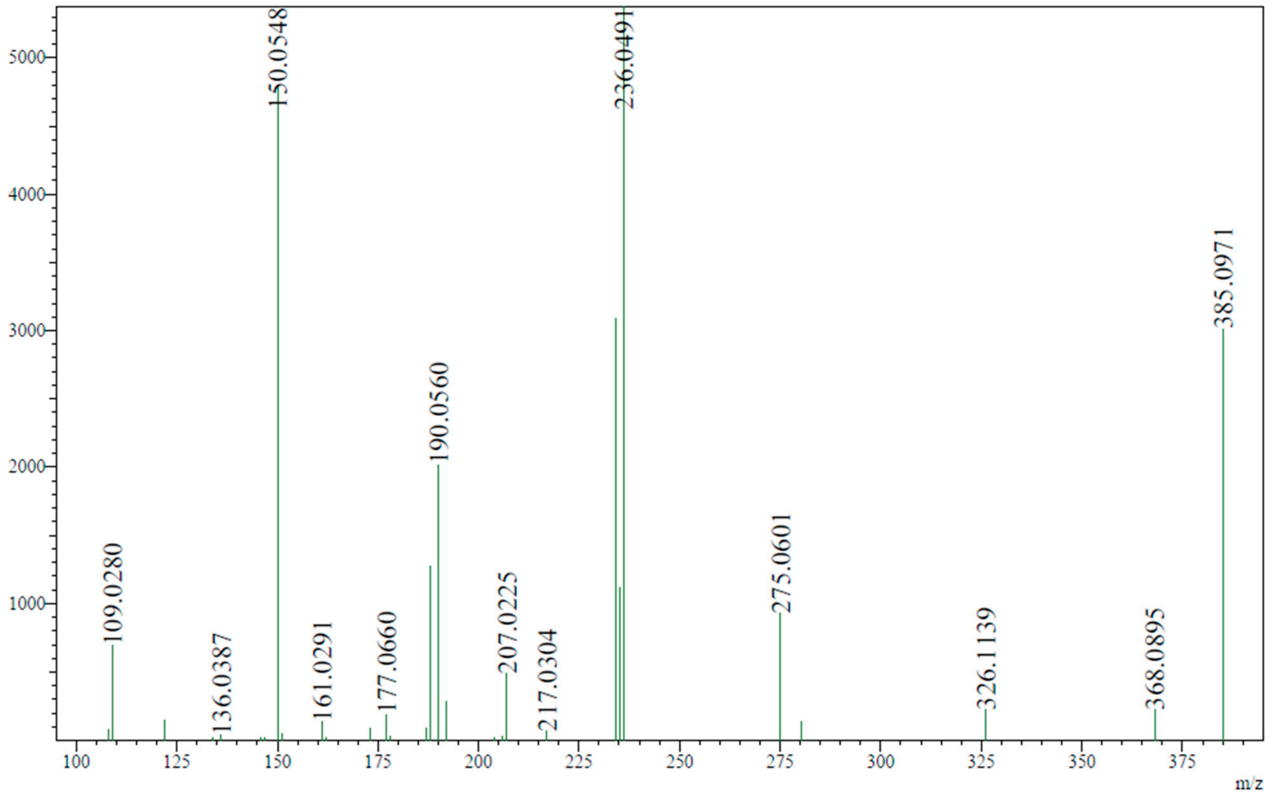

Figure S30. The HRMS spectrum of the compound **E6** (above) and MS/MS fragmentation spectrum of the corresponding molecular ion (below)

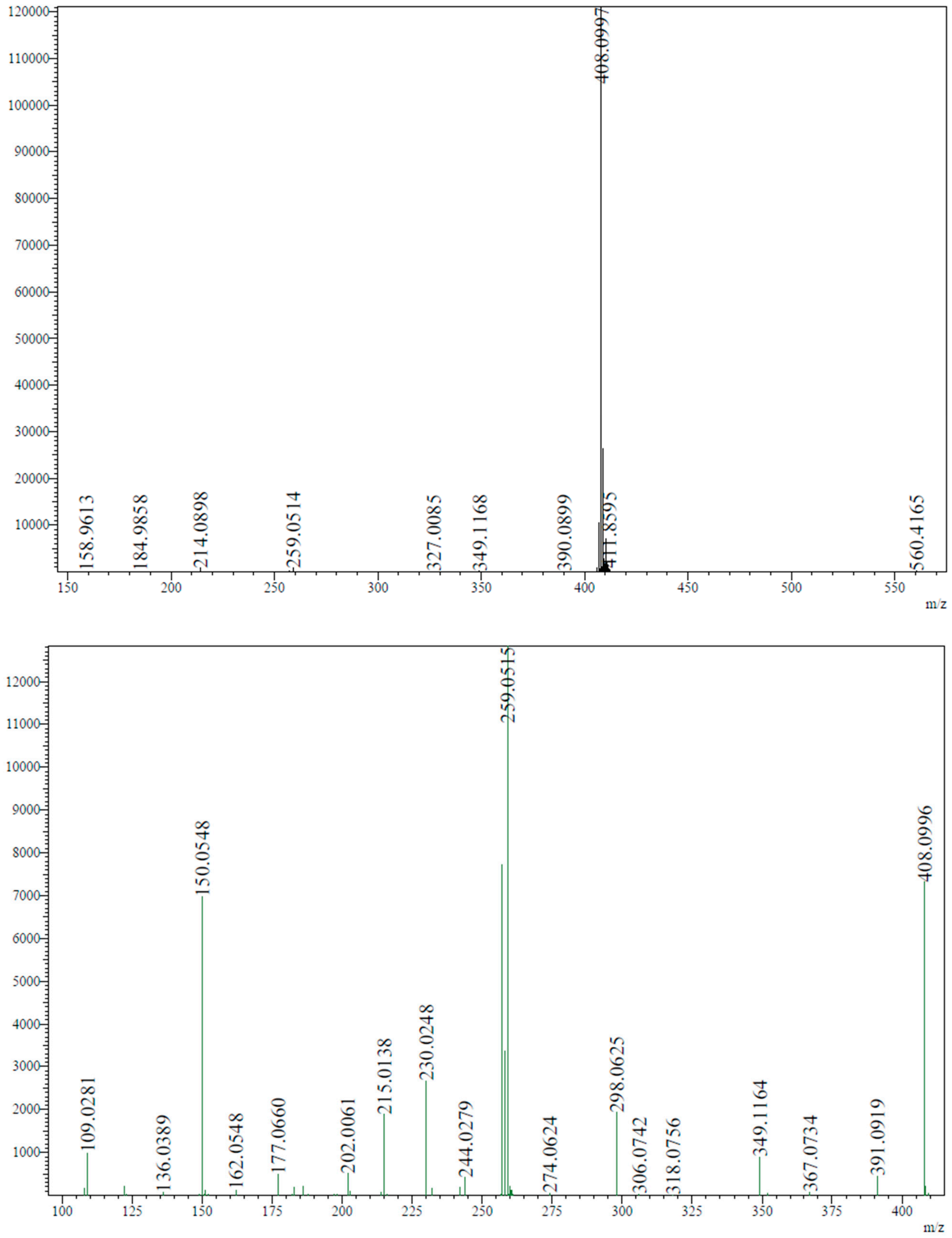

Figure S31. The HRMS spectrum of the compound **E7** (above) and MS/MS fragmentation spectrum of the corresponding molecular ion (below)

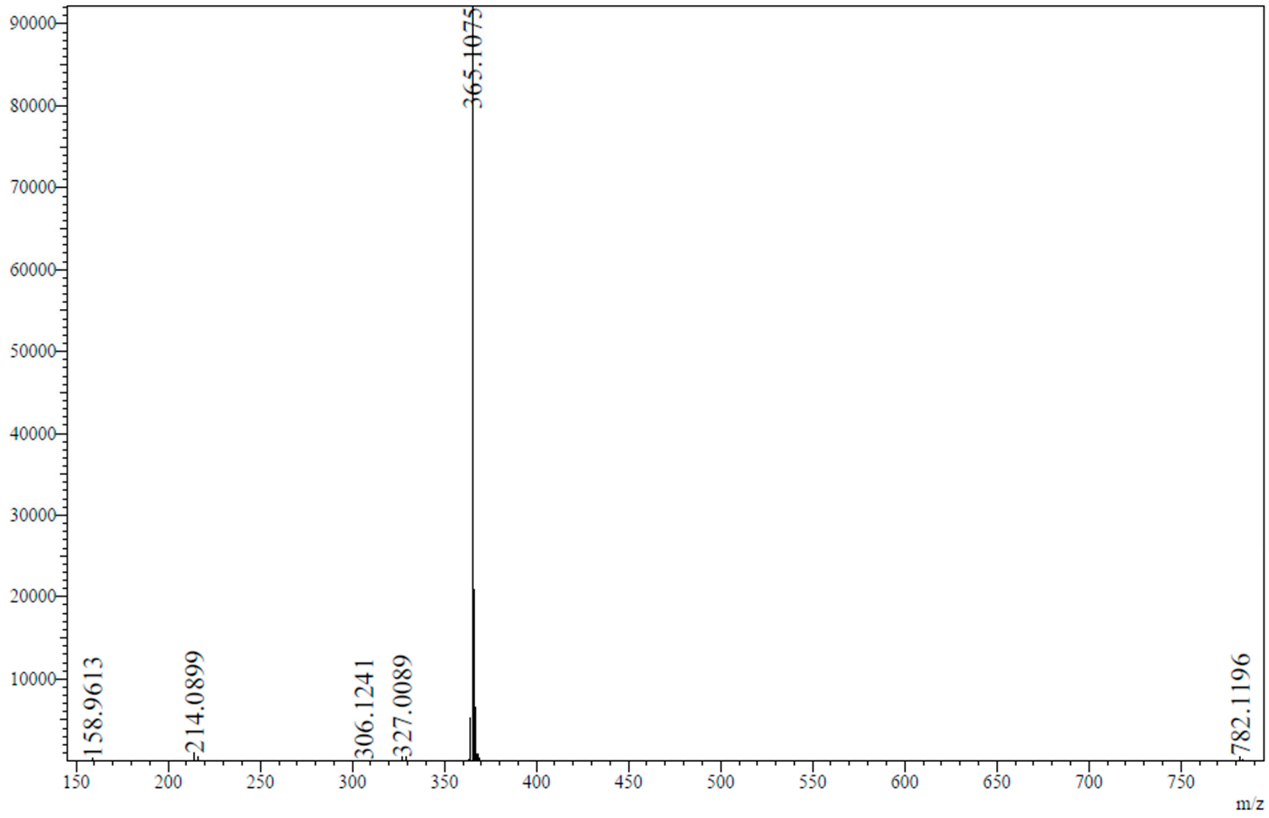

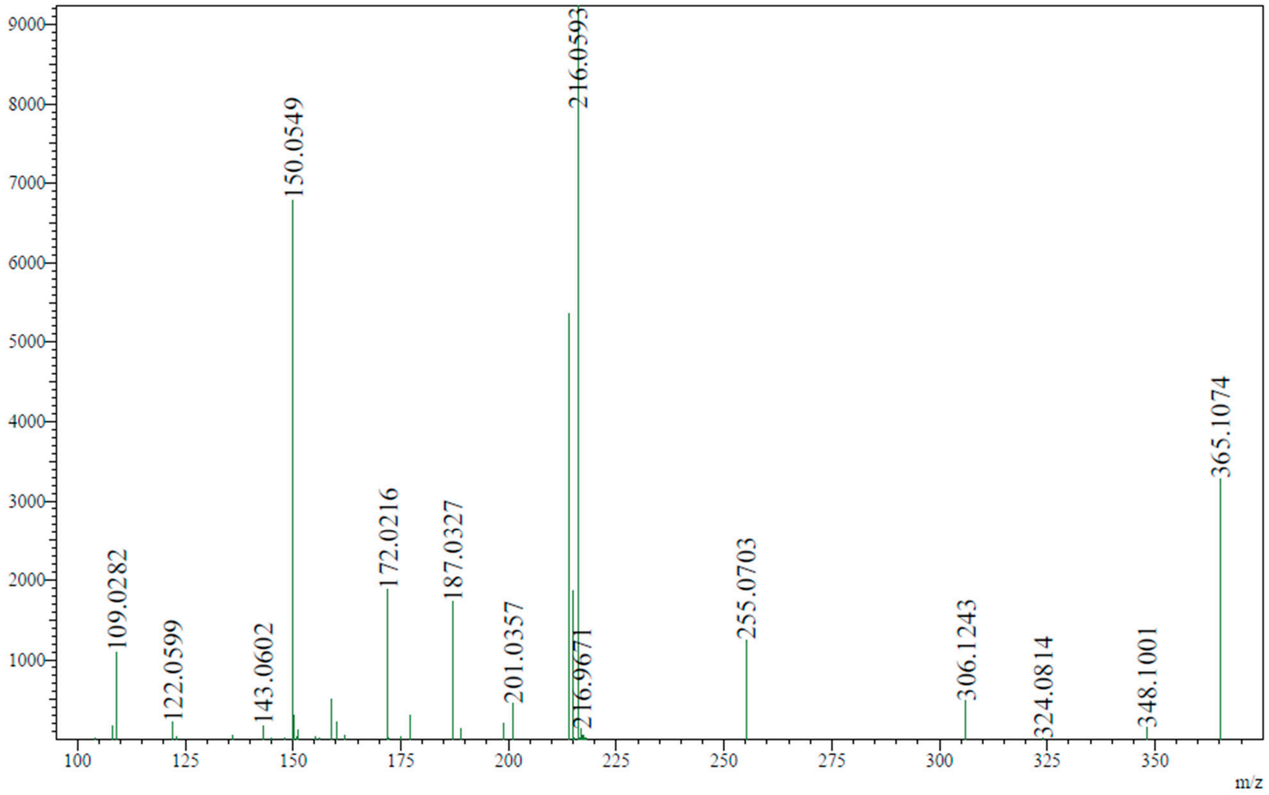

Figure S32. The HRMS spectrum of the compound **E8** (above) and MS/MS fragmentation spectrum of the corresponding molecular ion (below)

1.5 The RP-HPLC chromatograms at 254 nm of the compounds ***E1-E8***

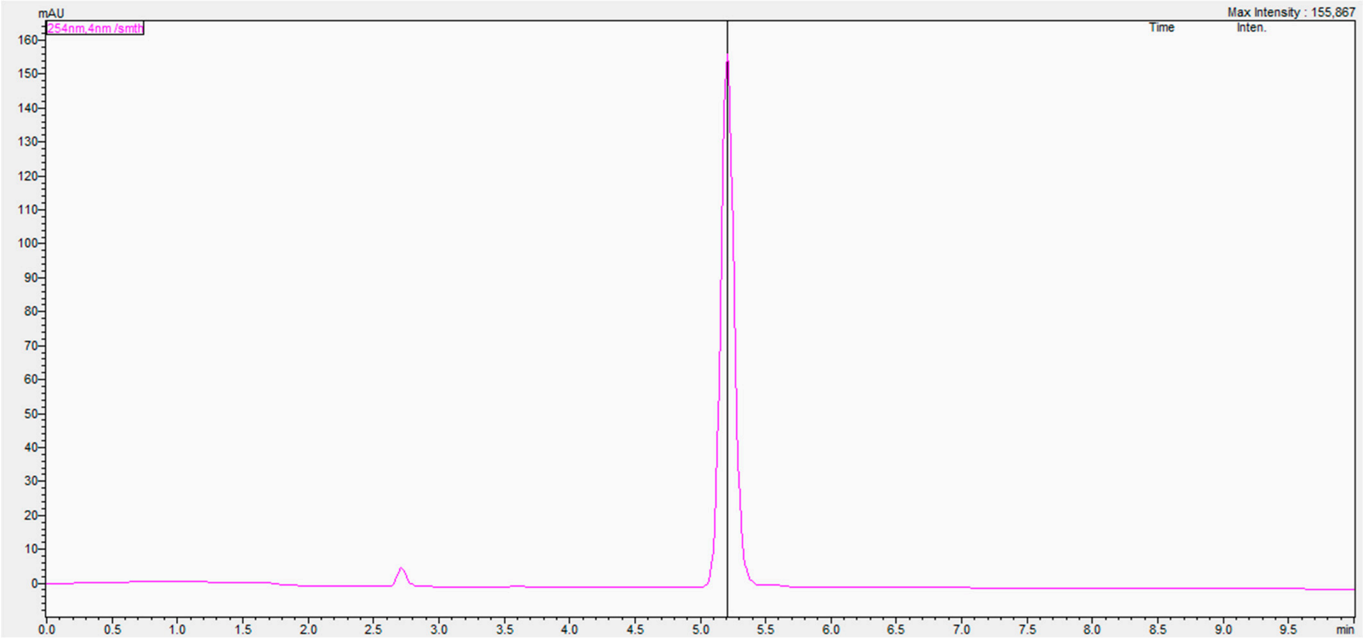

Figure S33. The RP-HPLC chromatogram of the compound **E1** recorded at 254 nm

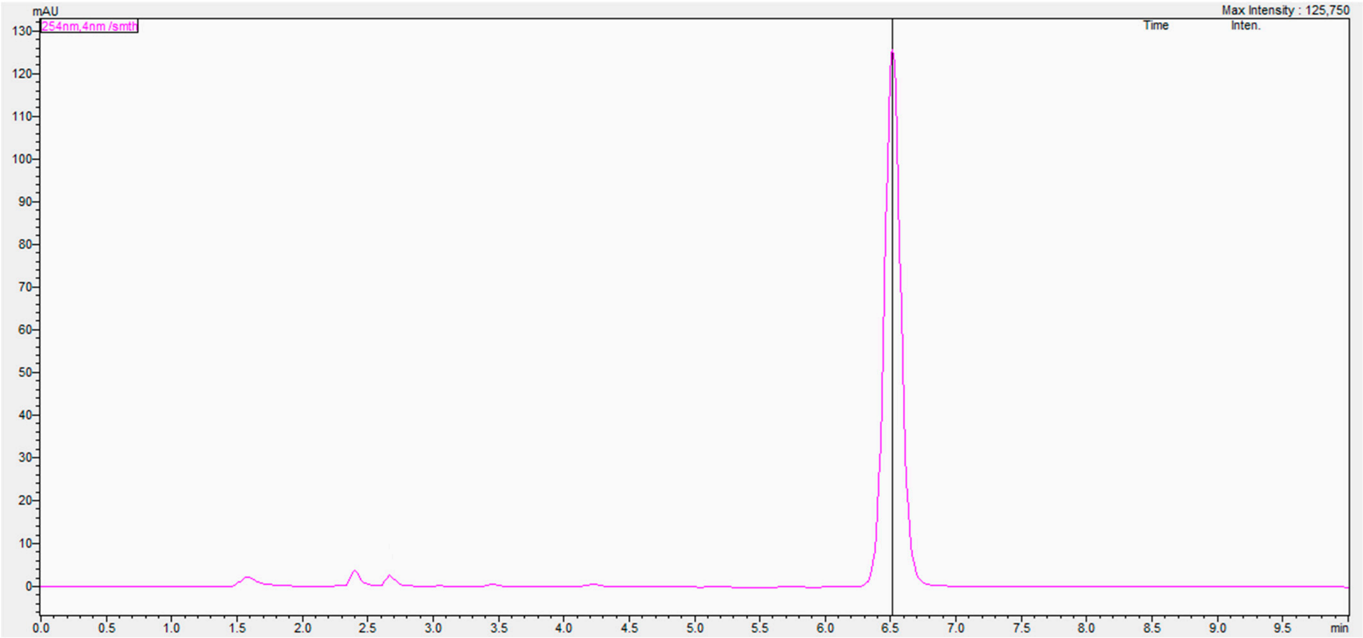

Figure S34. The RP-HPLC chromatogram of the compound **E2** recorded at 254 nm

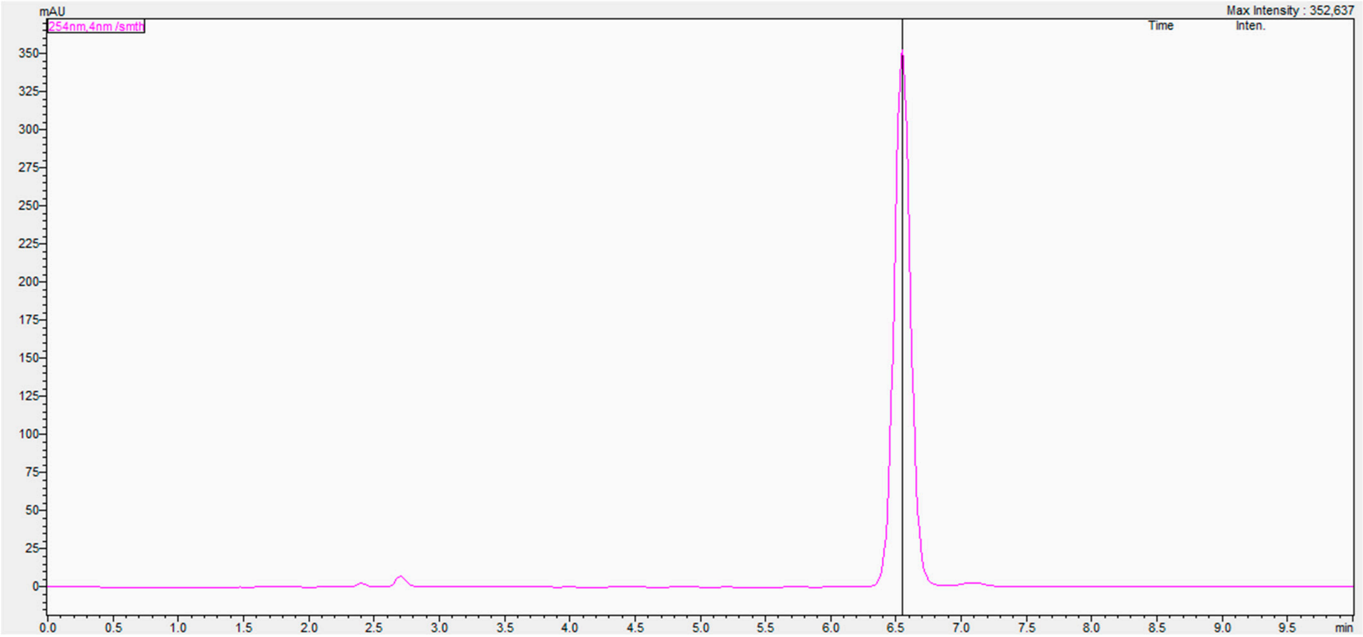

Figure S35. The RP-HPLC chromatogram of the compound **E3** recorded at 254 nm

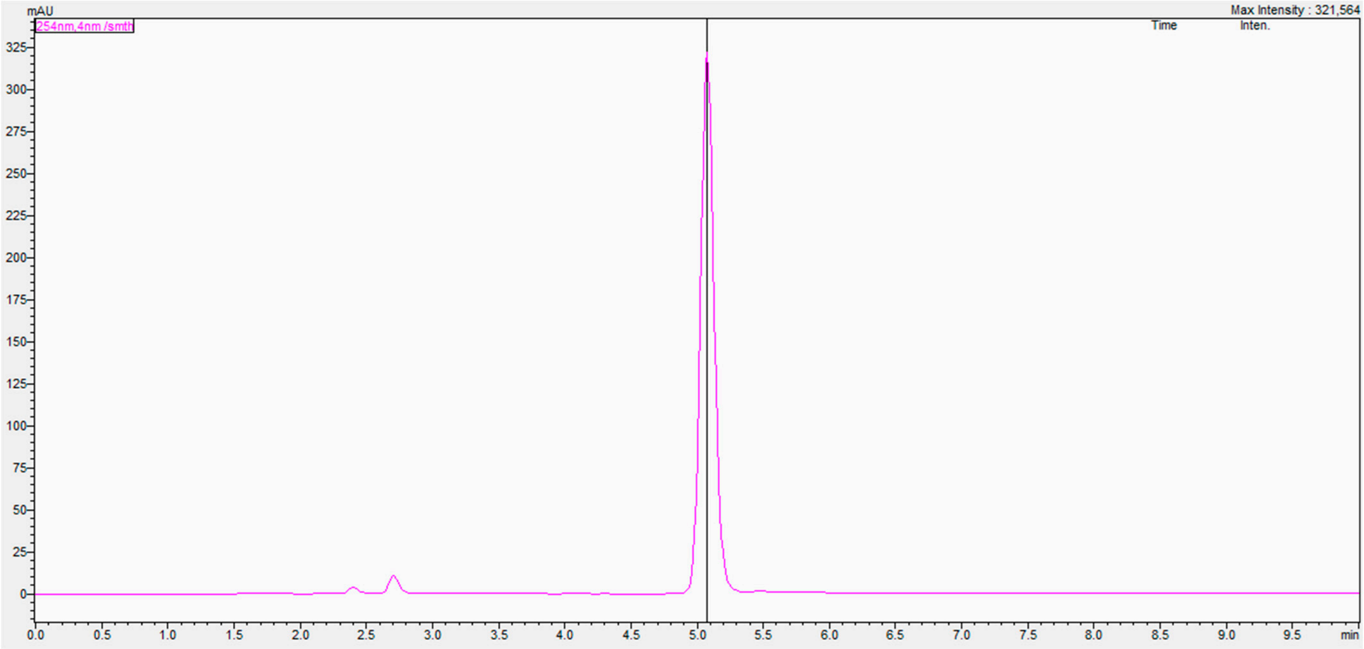

Figure S36. The RP-HPLC chromatogram of the compound **E4** recorded at 254 nm

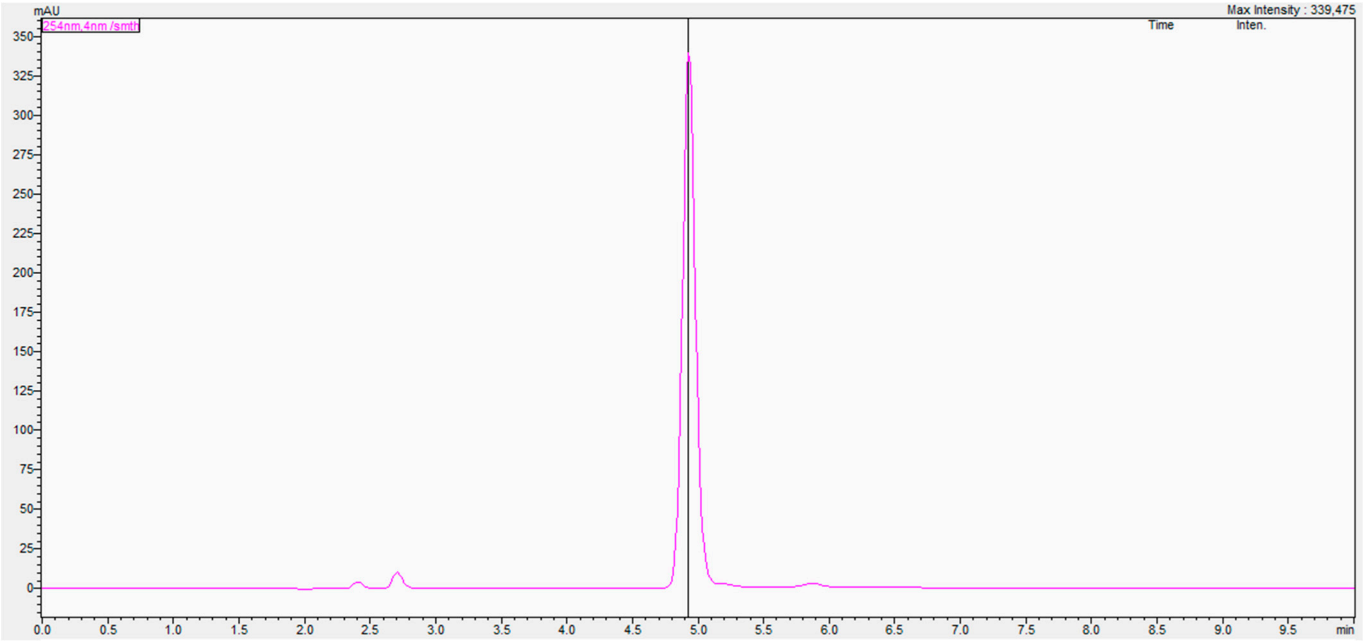

Figure S37. The RP-HPLC chromatogram of the compound **E5** recorded at 254 nm

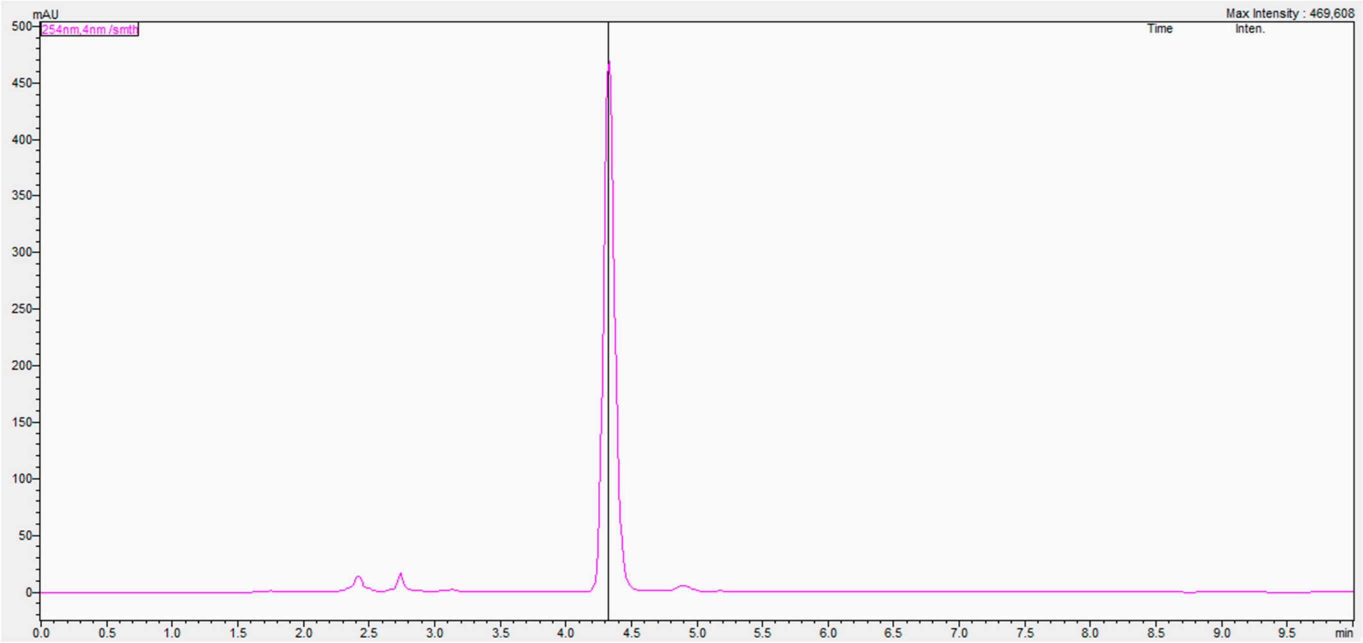

Figure S38. The RP-HPLC chromatogram of the compound **E6** recorded at 254 nm

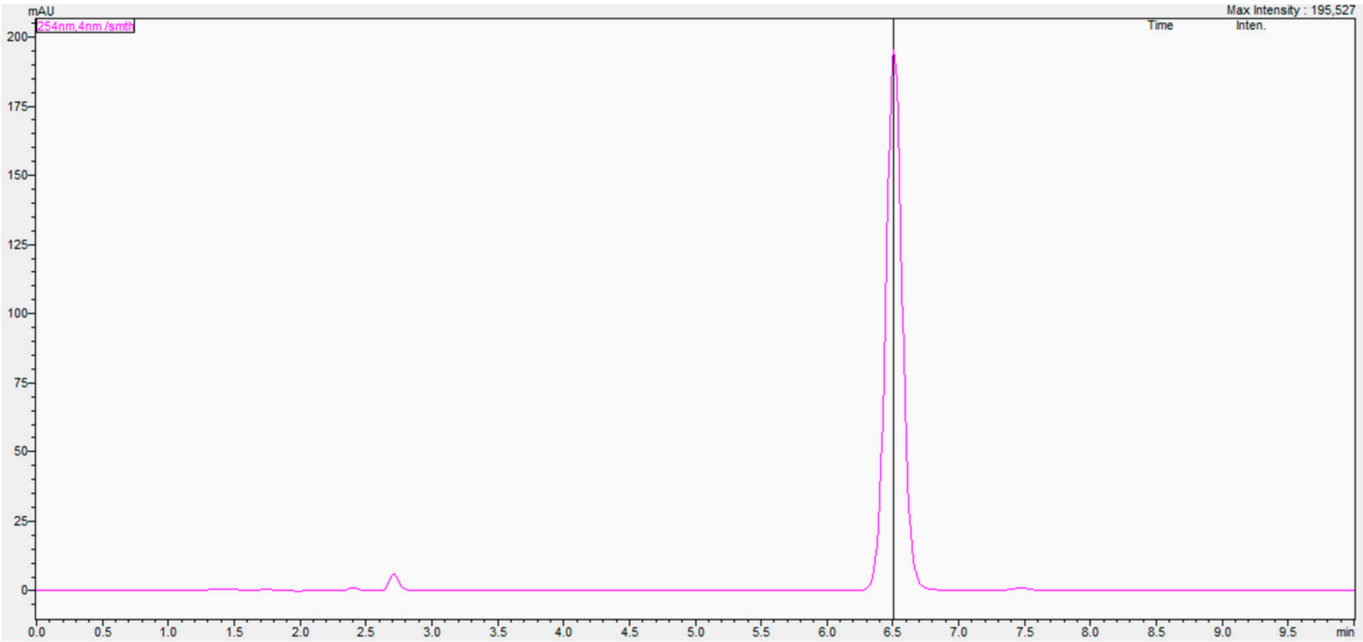

Figure S39. The RP-HPLC chromatogram of the compound **E7** recorded at 254 nm

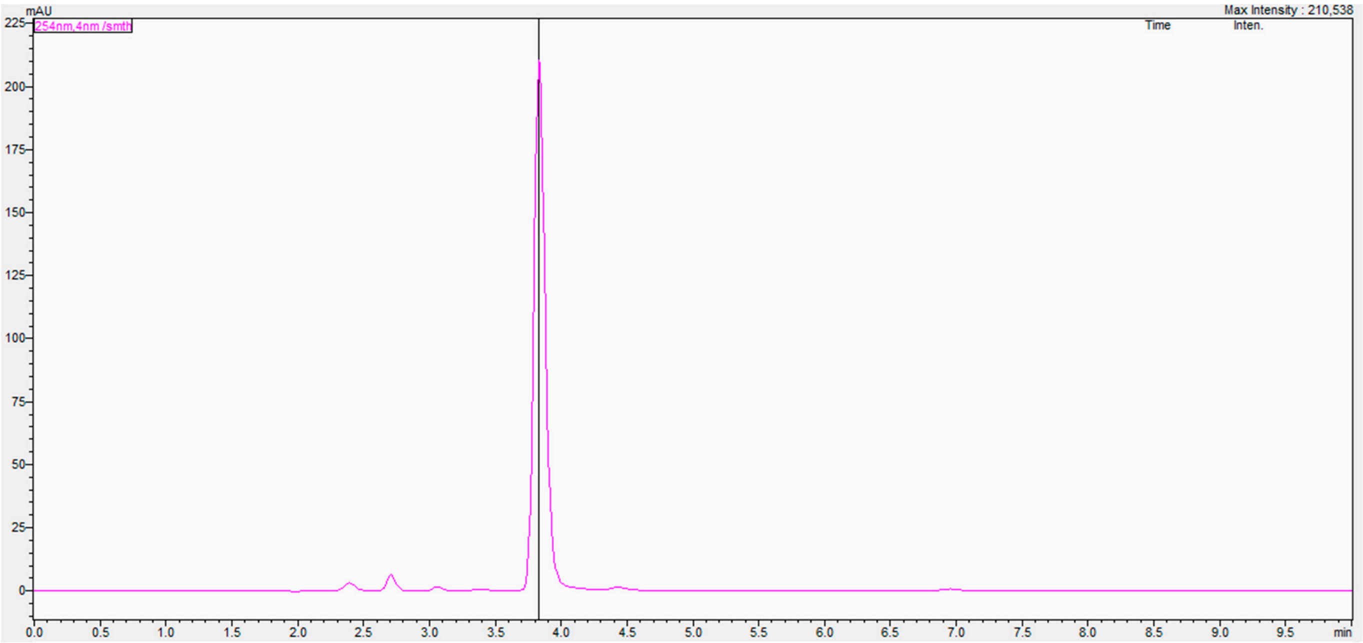

Figure S40. The RP-HPLC chromatogram of the compound **E8** recorded at 254 nm

2. Tables

*2.1. The depiction of HOMO and LUMO and the depiction of the spin density maps of compounds **E1-8***

Table S1. The depiction of HOMO and LUMO of the compounds **E1-8**

Compound	HOMO	LUMO

https://doi.org/10.3390/molecules31203620

**E1**	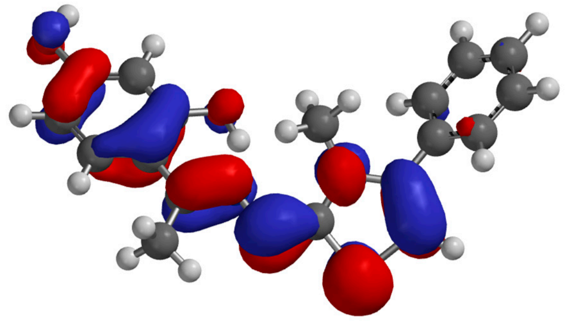	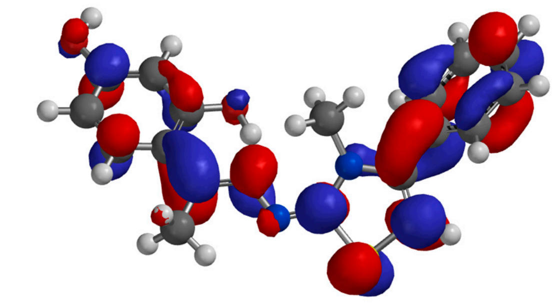
**E2**	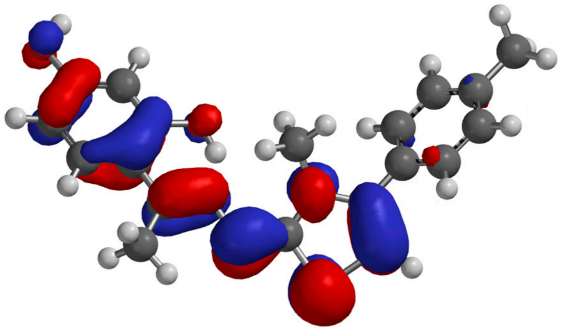	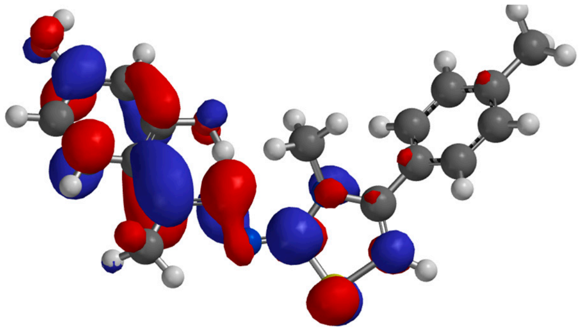
**E3**	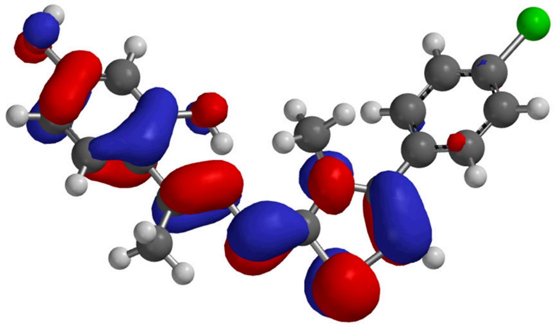	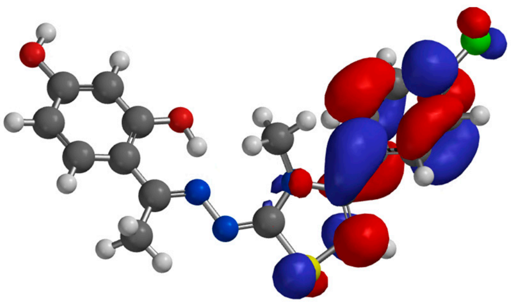
**E4**	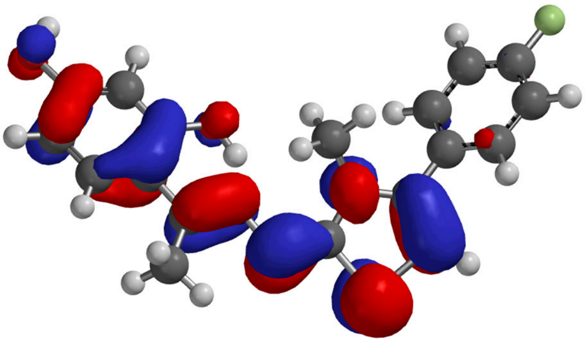	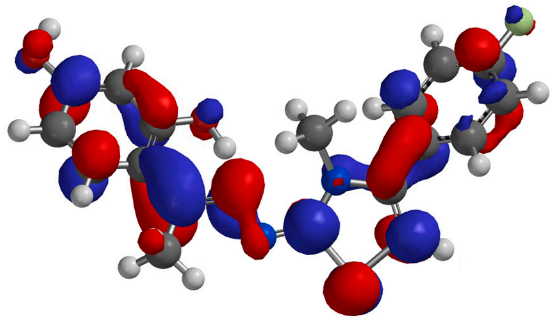
**E5**	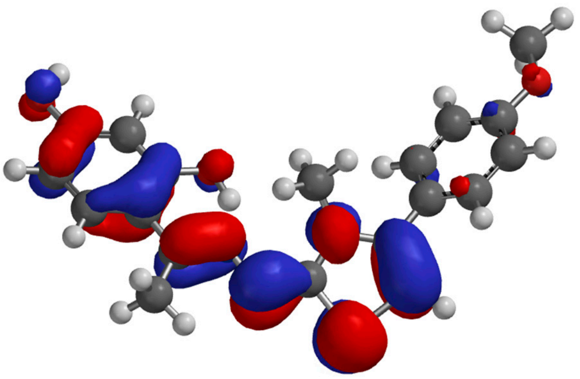	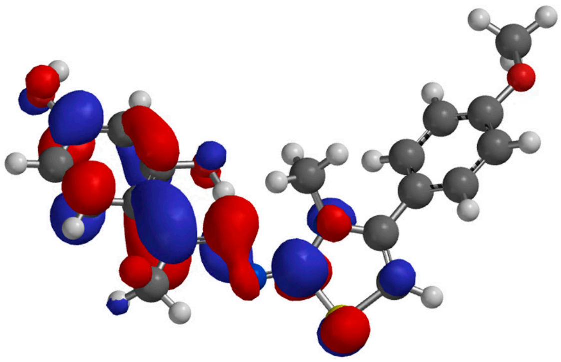

**E6**	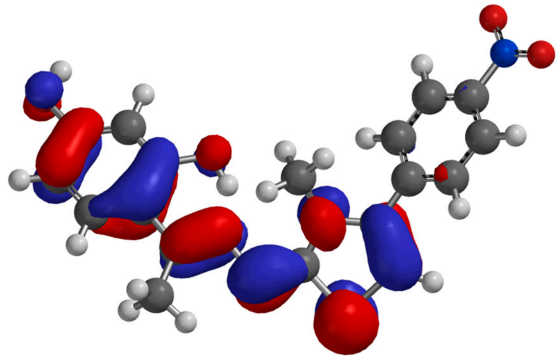	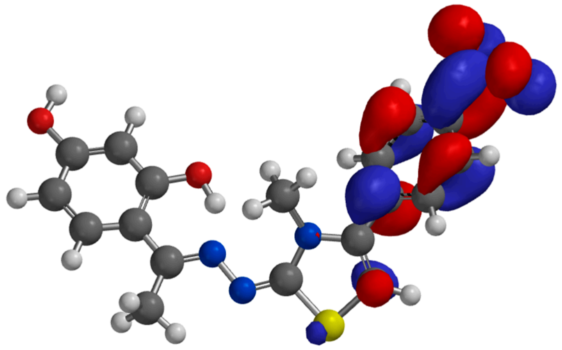
**E7**	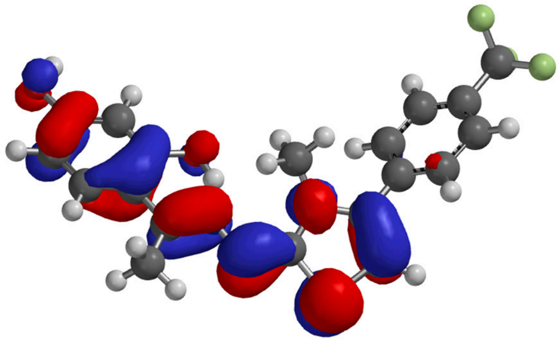	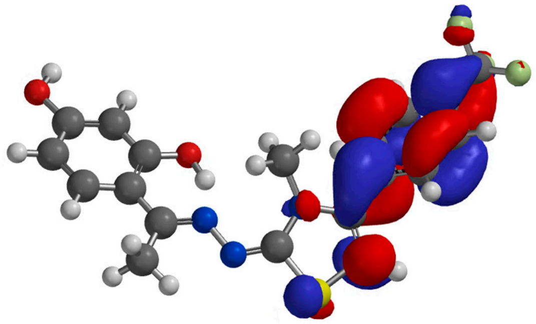
**E8**	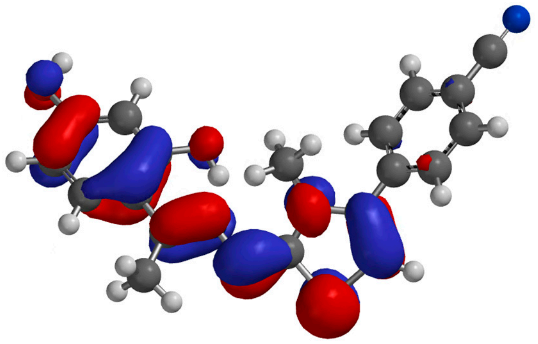	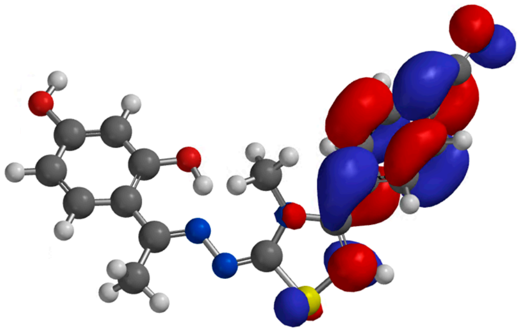

Table S2: The electrostatic potential maps of the compounds **E1-8**

Legend -180 kJ 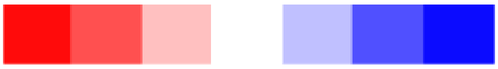 280 kJ

Compound	EPM

https://doi.org/10.3390/molecules31203620

**E1**	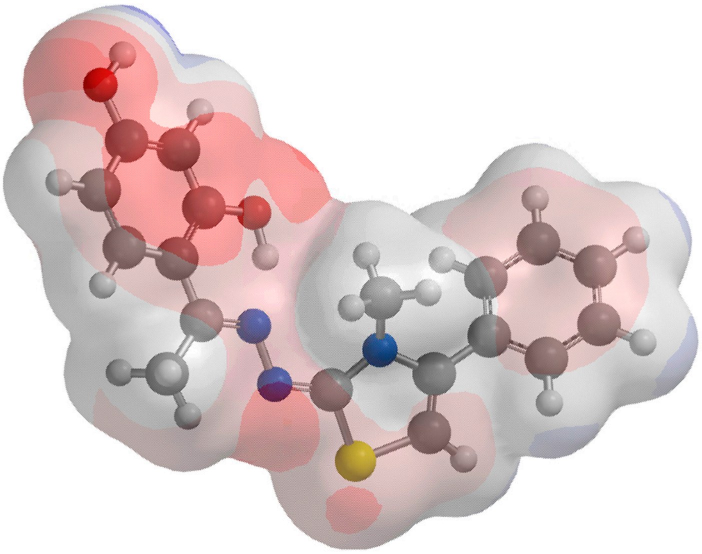
**E2**	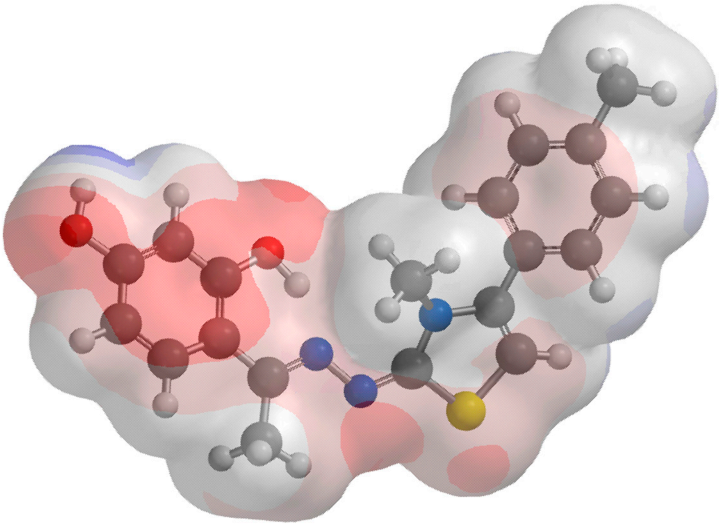
**E3**	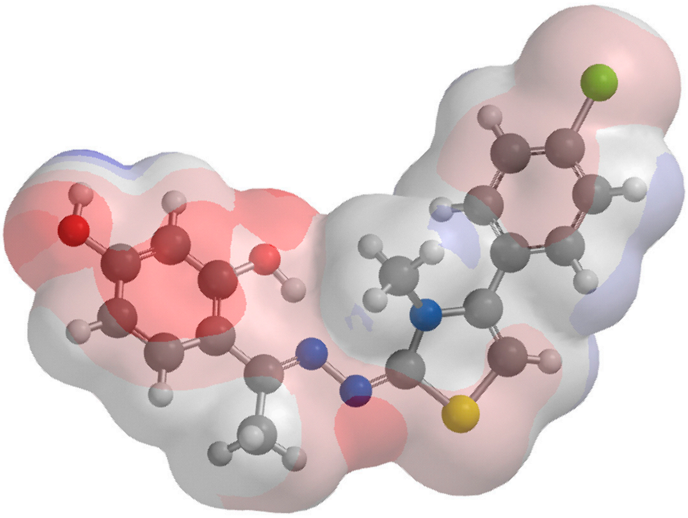

https://doi.org/10.3390/molecules31203620

**E4**	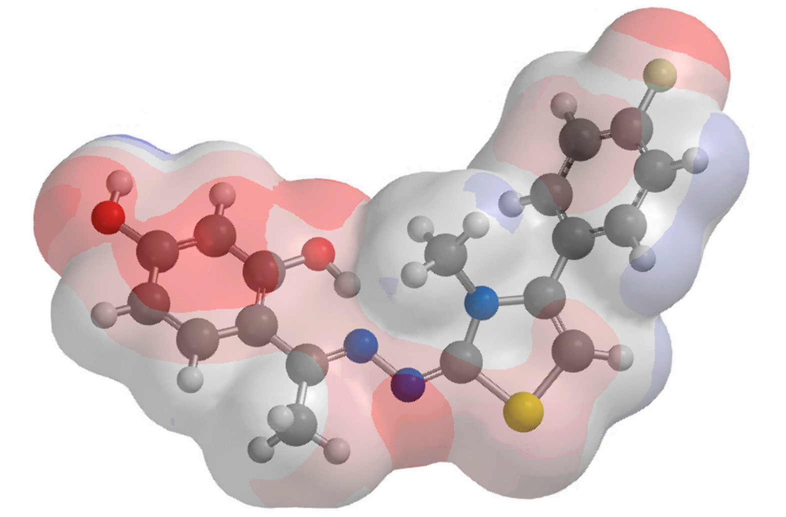
**E5**	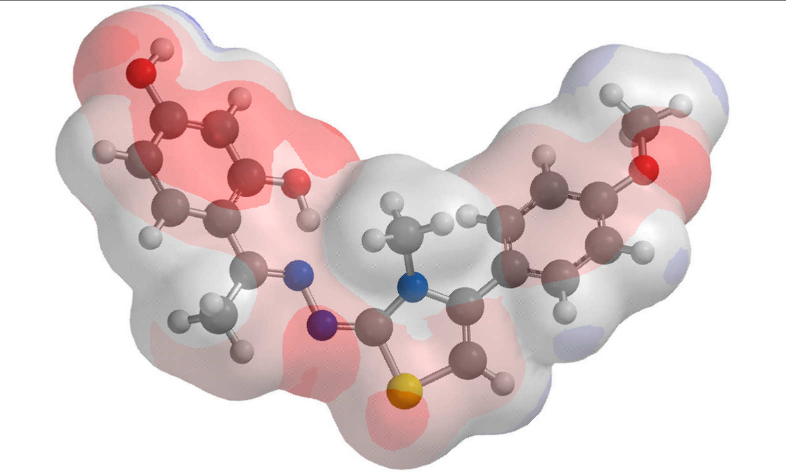
**E6**	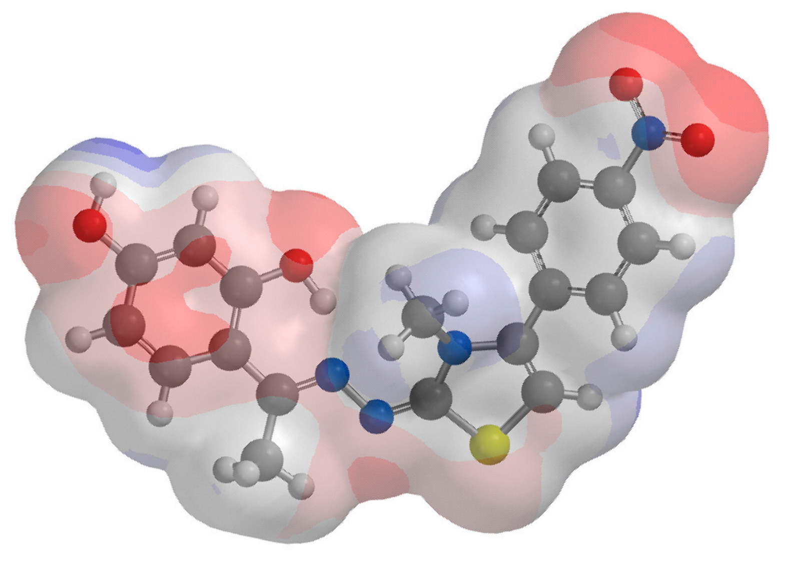

https://doi.org/10.3390/molecules31203620

**E7**	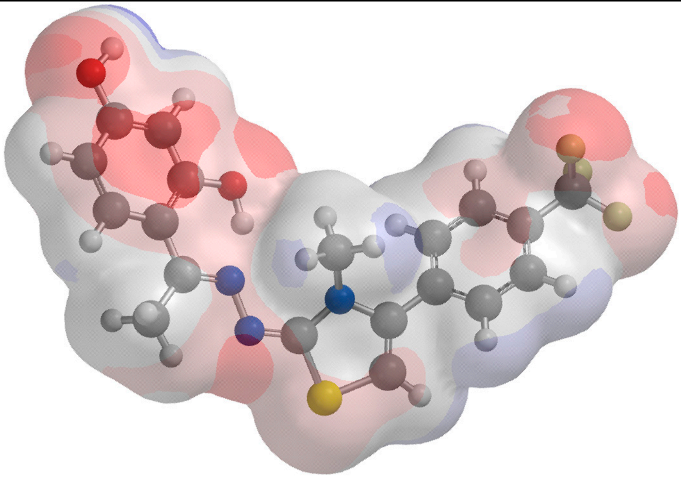
**E8**	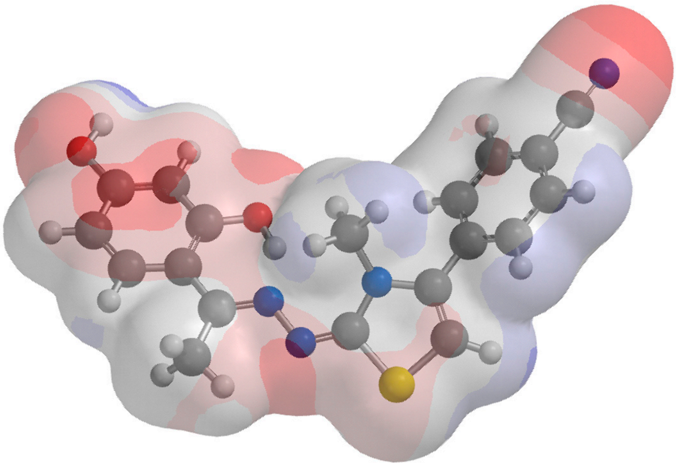

Disclaimer/Publisher's Note: The statements, opinions and data contained in all publications are solely those of the individual author(s) and contributor(s) and not of MDPI and/or the editor(s). MDPI and/or the editor(s) disclaim responsibility for any injury to people or property resulting from any ideas, methods, instructions or products referred to in the content.

https://doi.org/10.3390/molecules31203620
